# Unveiling the GT114 family: Structural characterization of A075L, a glycosyltransferase from *Paramecium bursaria* chlorella virus‐1 (PBCV‐1)

**DOI:** 10.1002/pro.5196

**Published:** 2024-11-18

**Authors:** Maria Elena Laugieri, Immacolata Speciale, Ana Gimeno, Sicheng Lin, Brock W. Byers, Ana Poveda, Reyes Núñez‐Franco, Idoia Iturrioz, María J. Moure, Gonzalo Jiménez‐Osés, Irene Russo‐Krauss, Anna Notaro, James L. Van Etten, Todd L. Lowary, Jesús Jimenez‐Barbero, Cristina De Castro, Michela Tonetti, Adriana L. Rojas

**Affiliations:** ^1^ DIMES – Biochemistry Division University of Genoa Genoa Italy; ^2^ Department of Chemical Sciences Università di Napoli Federico II Naples Italy; ^3^ Basque Research and Technology Alliance (BRTA) Center for Cooperative Research in Biosciences (CIC bioGUNE) Derio Spain; ^4^ Ikerbasque, Basque Foundation for Science Bilbao Spain; ^5^ Department of Chemistry University of Alberta Edmonton Alberta Canada; ^6^ Department of Agricultural Sciences Università di Napoli Federico II Portici Italy; ^7^ Nebraska Centre for Virology and Department of Plant Pathology University of Nebraska‐Lincoln Lincoln Nebraska USA; ^8^ Institute of Biological Chemistry Taipei Taiwan; ^9^ Institute of Biochemical Sciences National Taiwan University Taipei Taiwan

**Keywords:** A075L, β‐xylosyltransferase, giant virus, glycosyltransferases, PBCV‐1

## Abstract

Protein A075L is a β‐xylosyltransferase that participates in producing the core of the N‐glycans found in VP54, the major viral capsid protein of Paramecium bursaria chlorella virus‐1 (PBCV‐1). In this study, we present an X‐ray crystallographic analysis of the *apo* form of A075L, along with its complexes with the sugar donor and with a trisaccharide acceptor. The protein structure shows a typical GT‐B folding, with two Rossmann‐like fold domains, in which the acceptor substrate binds to the N‐terminal region, and the nucleotide‐sugar donor binds to the C‐terminal region. We propose that the catalytic mechanism follows a direct displacement S_N_2‐like reaction, where Asp73 serves as a catalytic base that deprotonates the incoming nucleophile of the acceptor, facilitating direct displacement of the UDP with the inversion of the anomeric configuration of the acceptor without metal ion dependence, while the interactions with side chains of Arg158 and Arg208 stabilize the developing negative charge. Using isothermal titration calorimetry, nuclear magnetic resonance spectroscopy, high‐performance liquid chromatography, and molecular dynamics simulations, the catalytic activity and specificity of this enzyme have been unraveled.

## 1 INTRODUCTION

Usually, viruses use the glycans present in their capsid proteins to interact with host receptors, a vital strategy for their replication and survival. Most viruses rely on host glycosyltransferases (GTs) to synthesize this post‐translational modification (Olofsson and Hans 1998). In contrast, giant viruses (Colson et al. 2013; Speciale et al. 2022a) encode some of the enzymes needed to glycosylate their glycoproteins. Remarkably, Paramecium bursaria chlorella virus‐1 (PBCV‐1) encodes most, if not all, of the glycosyltransferases (GT) involved in the glycosylation of its viral major capsid protein VP54 (Van Etten 2003). A075L is predicted to be one of these GTs. Its function has been proposed through the comparative analysis of the glycan structures that decorate the wild‐type PBCV‐1 and the selection of diverse naturally occurring PBCV‐1 mutants that are characterized by displaying truncated glycan structures (Speciale et al. 2019).

**FIGURE 1:**
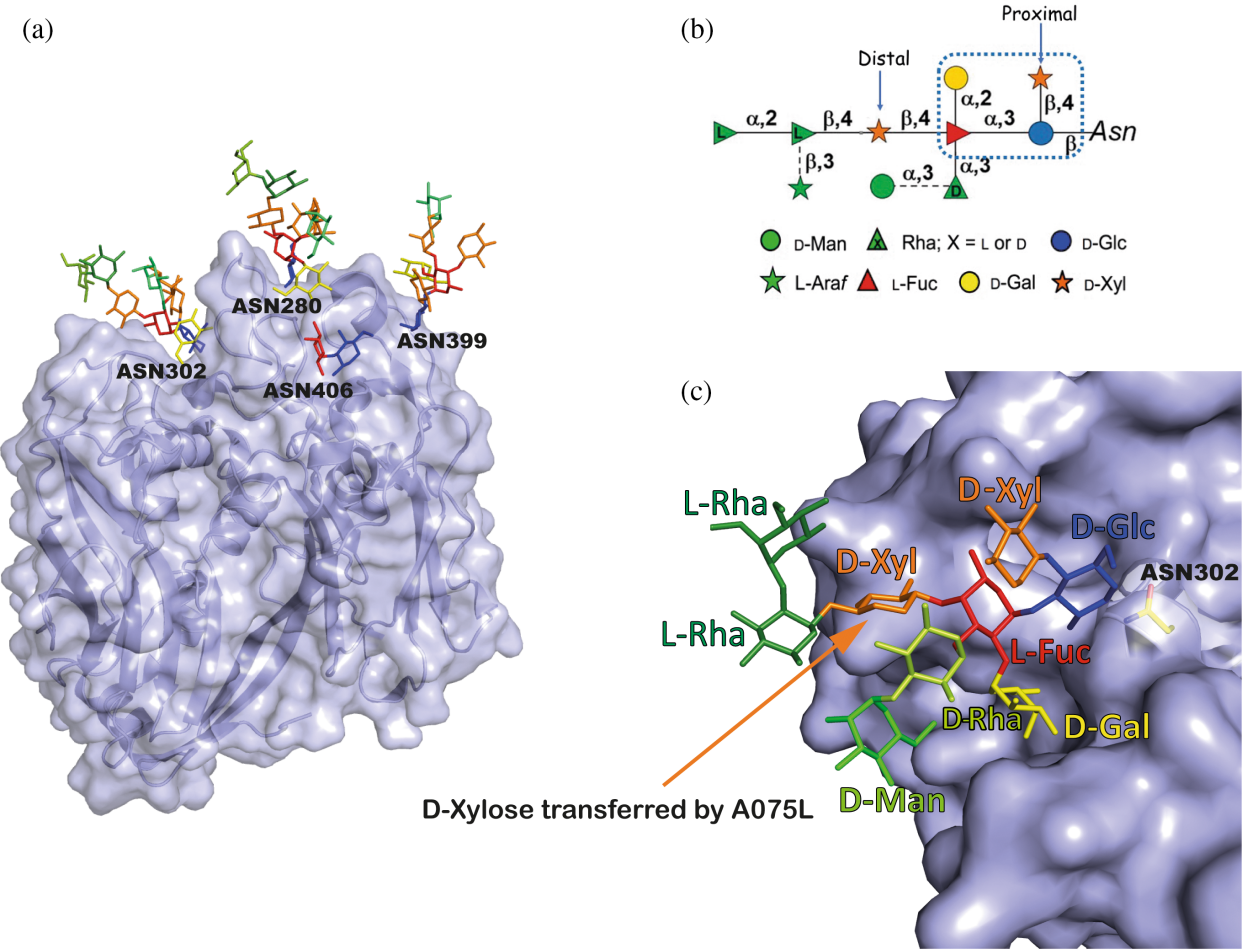
The VP54 N‐glycans. (a) VP54 is shown in cartoon and surface representation (PDB: 5TIP), while its four N‐glycans are shown in stick representation; the color code follows the one assigned in B, consistent with SNFG (symbol nomenclature for glycans) nomenclature. (b) Schematic view of the structure of the larger N‐glycan in PBCV‐1 VP54, where the residues inside the dotted box define the conserved core region. L‐arabinose (L‐Ara) and D‐mannose (D‐Man) are non‐stoichiometric substituents and generate four different glycoforms (De Castro et al. 2018; Speciale et al. 2019) (adapted from Speciale et al. 2019). (c) Representative snapshot showing the structure of the nine monosaccharides of the glycan in Asn302 (De Castro et al. 2018).

VP54 is glycosylated at four asparagine residues (Asn280, Asn302, Asn399, and Asn406) via an unusual β‐glucosyl linkage (Figure 1a). These four glycosylation sites exhibit distinct glycan structures, while sharing a core of four monosaccharides (Figure 1b). This glycan core is a shared characteristic among the majority of chloroviruses. A075L has been proposed to be a β‐xylosyltransferase, responsible for attaching the distal D‐xylose (D‐Xyl) unit to the L‐fucose (L‐Fuc) component of this conserved core (Speciale et al. 2022b; Van Etten 2003) (Figure 1b,c).

GTs are currently classified into 135 different families in the CAZY database (http://www.cazy.org/), based on their sequence features (Drula et al. 2022). Less than 50% of these GTs have been structurally characterized and classified in one of three main structural folds. GT‐A and GT‐B enzymes contain a Rossman fold variation and require nucleotide‐diphosphate activated sugars as donors. GT‐A has a single domain with short dynamic loops between two proximal Rossman folds (Taujale et al. 2020). Most GT‐A GTs are characterized by an Asp‐X‐Asp signature that coordinates a divalent ion and/or the donor ribose. GT‐B GTs have two β/α/β Rossman‐like domains separated by a large cleft containing the reaction center. The N‐terminal region is involved in the acceptor substrate recognition, whereas the C‐terminal domain mainly binds the nucleotide‐sugar donor (Lairson et al. 2008). Finally, GT‐C topology is characterized by an integral polytopic membrane domain that recognizes a lipid‐phosphosugar donor, while the acceptor binding region is located in a variable soluble domain, with the reaction center being at the interface between these two domains (Bai and Li 2021).

GTs are classified as “inverting” or “retaining” enzymes according to the anomeric configuration of reactants and products. It is accepted that inverting GTs use a direct displacement S_N_2‐like mechanism involving an enzymatic base catalyst that deprotonates the incoming nucleophile of the acceptor, facilitating the direct displacement of the phosphate‐leaving group. The departure of the leaving group in GT‐A fold enzymes is typically promoted via a coordinated divalent cation, whereas the GT‐B fold analogues use instead positively charged side chains that are strategically positioned. On the other hand, most retaining GTs that have been structurally characterized do not operate via a two‐step mechanism involving the formation of a glycosyl‐enzyme intermediate analogous to glycosidases. Instead, an internal return S_N_i‐like mechanism has been proposed, in which the leaving group departure and the nucleophilic attack occur in a concerted but asynchronous manner on the same face of the glycoside (Ardèvol et al. 2016; Lairson et al. 2008; Moremen and Haltiwanger 2019). However, recent studies have identified retaining GTs that do proceed by way of a covalent enzyme‐linked intermediate (Doyle et al. 2023; Forrester et al. 2022).

**FIGURE 2:**
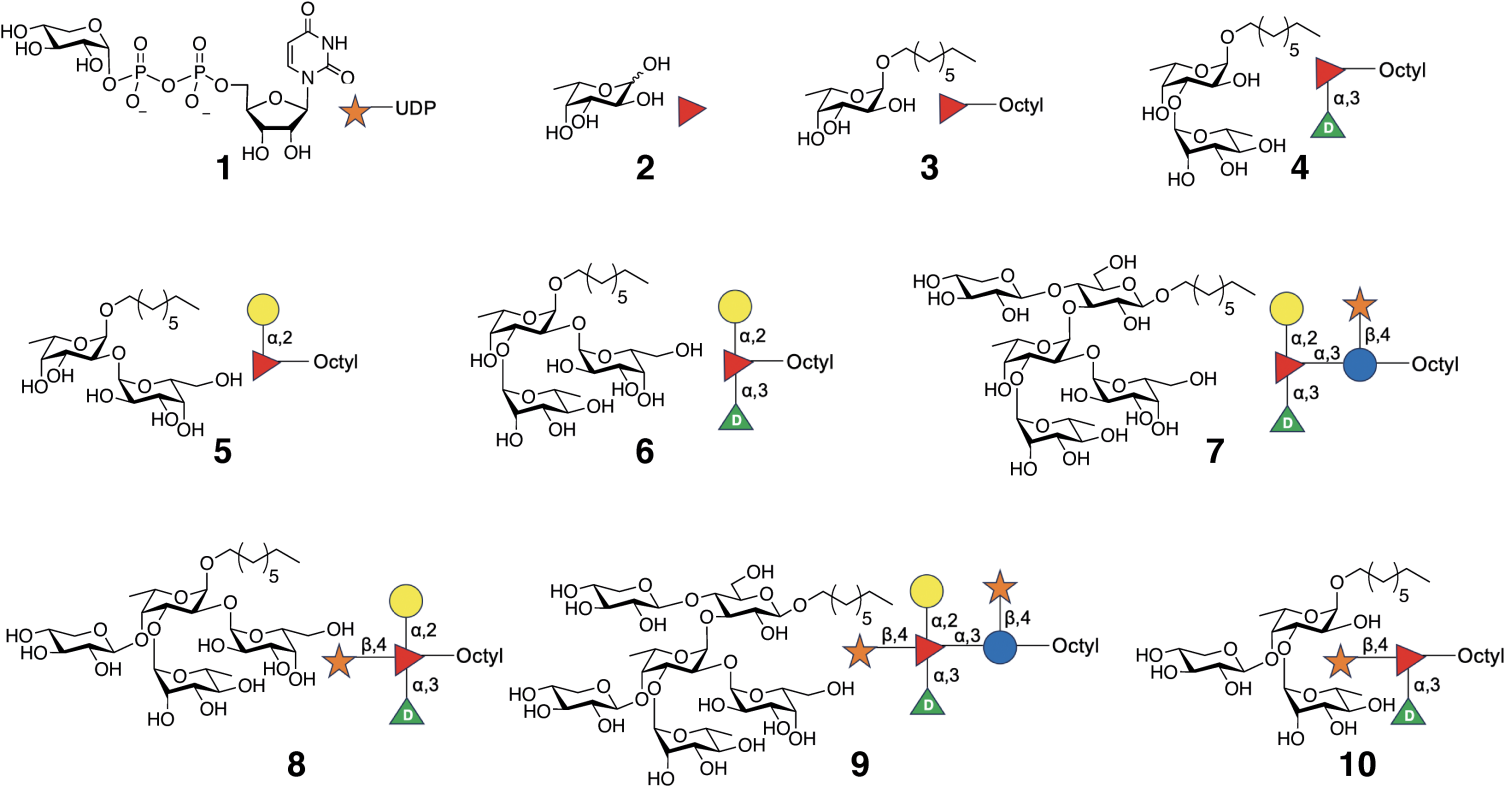
Structures of UDP‐xylose (**1**) and putative acceptor substrates **2**–**7** used in these investigations. Also shown are the products formed when acceptors **6**, **7** or **4** were incubated with **1** and A075L; tetrasaccharide **8**, hexasaccharide **9**, and trisaccharide **10**, respectively.

Most GTs are membrane‐bound enzymes and are challenging to produce for high‐resolution structural studies. As a result, relatively few structures of GT enzymes have been determined so far with their acceptors or donors bound in its active site (Moremen and Haltiwanger 2019). On the other hand, A075L is a cytosolic protein previously annotated as an exostosin (Pfam database code PF03016.8), classified within the GT47 family. Herein, we report crystallographic structures of the A075L in its *apo* form and in complex with the sugar donor, UDP‐α‐D‐Xyl (**1** for now on, Figure 2), and a minimal trisaccharide acceptor, α‐D‐Gal‐(1 → 2)‐[α‐D‐Rha‐(1 → 3)]‐α‐L‐Fuc‐Octyl (**6** for now on, Figure 2). Moreover, different features of its enzymatic activity have been scrutinized through the combination of ITC, NMR spectroscopy, HLPC, and mass spectrometry methods. The combination of these biophysical protocols with the analysis of the crystallographic structures, assisted by molecular dynamics (MD) simulations have allowed determining the critical residues involved in the catalytic activity and for us to propose the enzymatic mechanism.

## 2 RESULTS

### 2.1 Analysis of the enzyme specificity and selectivity

**FIGURE 3:**
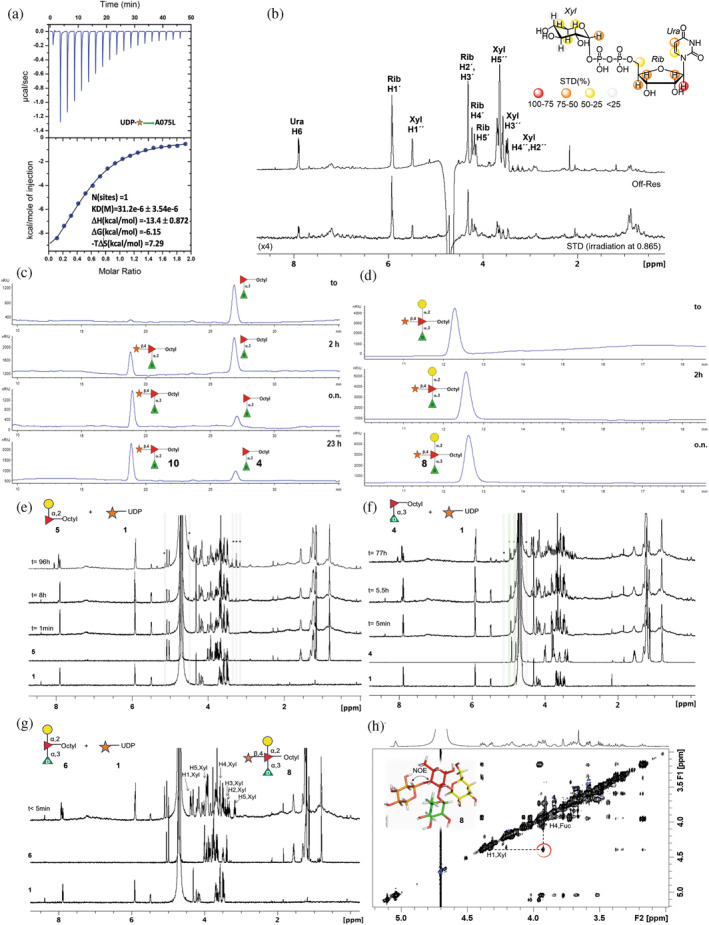
Analysis of the interaction between A075L, sugar donor **1** and acceptors **4**–**6**. (a) The interaction between A075L and **1** in the presence of MgCl_2_ was monitored by ITC. The top graph is the differential heat released during the titration of 1 mM **1** into 55 μM A075L; the bottom graph is the fitted binding isotherms, where the thermodynamic parameters are also indicated. (b) ^1^H STD‐NMR spectrum and off‐resonance spectrum recorded for **1** in the presence of A075L. Hydrogens showing up in the STD spectrum are indicated, as well as the epitope mapping (top right) derived from the ^1^H STD‐NMR data. (c) Chromatographic profiles of A075L when incubated with disaccharide **4** and donor **1**. (d) Chromatographic profiles of A075L when incubated with trisaccharide **6** and donor **1**. (e–g) 600 MHz ^1^H NMR spectra of the reaction mixtures of A075L with **1** and disaccharide **5** (e), disaccharide **4** (f), and trisaccharide **6** (g). The black and green asterisks indicate non‐overlapped NMR signals of the generated D‐Xyl and the corresponding glycosylation product, respectively. (h) ^1^H–^1^H NOESY NMR spectrum (250 ms mixing time) of the reaction mixture shown in (g), the NOE cross‐peak between H1 of D‐Xyl and H4 of L‐Fuc unequivocally indicates the formation of the corresponding product (**8**).

Previous studies suggested that A075L was a β‐xylosyltransferase (Speciale et al. 2019); thus, we first investigated its binding affinity for the sugar nucleotide donor, **1**, by ITC. The *K*_D_ was determined as 31 μM (Figure 3a) in the presence of ions (Mg^2+^, Mn^2+^), as other GTs with an Asp‐X‐Asp motif require divalent ions for activity (Breton et al. 1998). For A075L, this motif is located between residues 73 and 75 (D73–Y74–D75). ^1^H Saturation Transfer Difference NMR (STD‐NMR) (Bernd Meyer 1999; Bernd Meyer 2001) spectroscopic experiments supported the binding of **1** to A075L. Signals belonging to the D‐Xyl, ribose and uracil moieties were apparent in the STD spectrum, strongly suggesting the existence of an extended binding epitope involving the entire sugar nucleotide (Figure 3b). Regarding the mechanism, standard ^1^H NMR‐based monitoring of a sample containing A075L and the sugar donor showed that A075L indeed hydrolysed **1** over time, although the reaction was rather slow. Indeed, it took 22 h for the H1 signal of **1** to completely disappear in the NMR spectrum (Figure S1a, Supporting Information). The initial detection of the β‐anomer for the resulting D‐Xyl suggested that the reaction proceeds with inversion of the configuration.

To address the specificity of the enzyme towards possible acceptor substrates, reactions involving **1** and several synthetic acceptors were carried out. The acceptors included L‐Fuc (**2**, Figure 2), its α‐octyl glycoside (**3**), two disaccharides (**4**, **5**), a trisaccharide (**6**), and a pentasaccharide (**7**). These compounds were designed to mimic parts of the core N‐glycan found in VP54. Reactions involving the acceptors α‐D‐Rha‐(1 → 3)‐α‐L‐Fuc‐Octyl (**4**, Figure 2) or α‐D‐Gal‐ (1 → 2) [α‐D‐Rha‐(1 → 3)]‐α‐L‐Fuc‐Octyl (**6**, Figure 2) were monitored using high‐performance liquid chromatography (HPLC). The reaction proceeded smoothly with both oligosaccharides (Figure 3c,d); for the trisaccharide it was extremely fast. Indeed, the starting material peak was not detectable even at *t* = 0 (Figure 3d). In contrast, no products were seen using L‐Fuc (**2**) or α‐D‐Gal‐(1 → 2)‐α‐L‐Fuc‐Octyl (**5**) as the acceptor.

We also used NMR spectroscopy to monitor product formation and the results corroborated those obtained by HPLC. The reaction using just the monosaccharide, L‐Fuc (**2**), as acceptor in the presence of A075L, showed that no glycosylation occurred, even after 16 h of incubation (Figure S1b). Instead, analysis of the NMR spectrum indicated the presence of **2** and D‐xylose (D‐Xyl), the later coming from hydrolysis of the donor **1** (Figure S1b). Likewise, when the disaccharide **5** was used as acceptor, only the hydrolysis of **1** was detected after 96 h, indicating its poor reactivity (Figure 3e). In contrast, and consistent with the HPLC results, the corresponding glycosylation product was detected after a few minutes of incubation when disaccharide **4** was used as the acceptor. Nevertheless, D‐Xyl was also detected as a by‐product, indicating that a competition between both processes (glycosylation versus hydrolysis) was taking place (Figure 3f). When the reaction was attempted using the trisaccharide acceptor **6**, product formation took place immediately, and only the expected tetrasaccharide **8** was detected (Figure 3g), as confirmed by the detailed NMR spectroscopic analysis. Indeed, the presence of a NOE cross‐peak between H1 of D‐Xyl and H4 of L‐Fuc unambiguously confirmed the formation of **8** (Figure 3h). The product was purified by size exclusion chromatography and fully characterized by MALDI mass spectrometry and NMR spectroscopy (Figure S2a–c). These data matched those for **8** obtained using a compound obtained by chemical synthesis (see Data S1).

**FIGURE 4:**
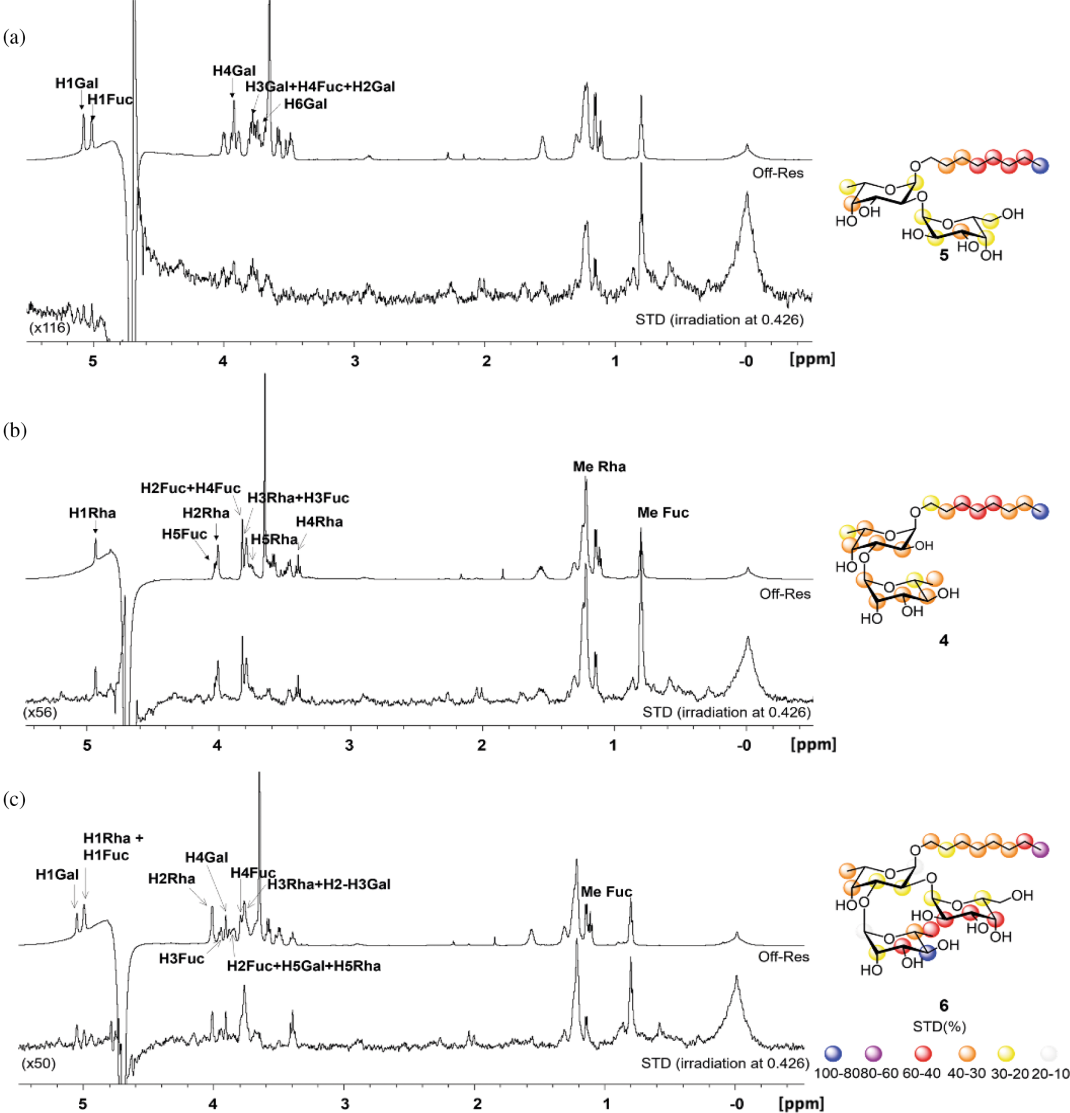
^1^H STD‐NMR spectra and off‐resonance spectra of the sugar acceptors in the presence of A075L (a 1:40 enzyme–acceptor ratio was employed). (a) STD‐NMR spectrum with disaccharide **5**, (b) STD‐NMR spectrum with disaccharide **4** and (c) STD‐NMR spectrum with trisaccharide **6**. On top, the reference off‐resonance spectrum with annotations of the key ^1^H signals; below, the STD‐NMR spectrum with protein irradiation at the aliphatic region; right, scheme displaying the epitope mapping derived from the ^1^H STD‐NMR results. Note that data from STDD spectra, where STDD = _STDA075L + ligand_ − STD_ligand_, were used to determine group epitope mapping.

To further characterize substrate recognition, the minimum glycan epitope that A075L recognizes was investigated by analyzing the interaction of the enzyme with three model acceptors using ^1^H STD‐NMR spectroscopy. Notably, in the presence of the enzyme, disaccharide **4** exhibited stronger STD‐NMR signals than disaccharide **5** consistent with the former's comparative higher reactivity in the glycosylation reaction (Figure 4a,b). Fittingly, trisaccharide **6**, with the highest reactivity, also showed substantial STD‐NMR effects (Figure 4c). Epitope mapping analysis for the three acceptor substrates revealed that, despite minor differences in intensity, most of the protons of the three model acceptors showed up in all three STD NMR spectra. These results strongly suggest the existence of a groove‐type binding mode, wherein the entire ligand, particularly the Rha and Gal units, are embedded within the enzyme's binding site, rather than interacting with a shallow region. The STD‐NMR results also indicated that despite their lower reactivity, both disaccharides can be accommodated in the binding site, but provided less contacts than the trisaccharide.

## 3 THE MECHANISM OF THE TRANSGLYCOSYLATION REACTION CATALYZED BY A075L

### 3.1 The overall 3D‐structure

**FIGURE 5:**
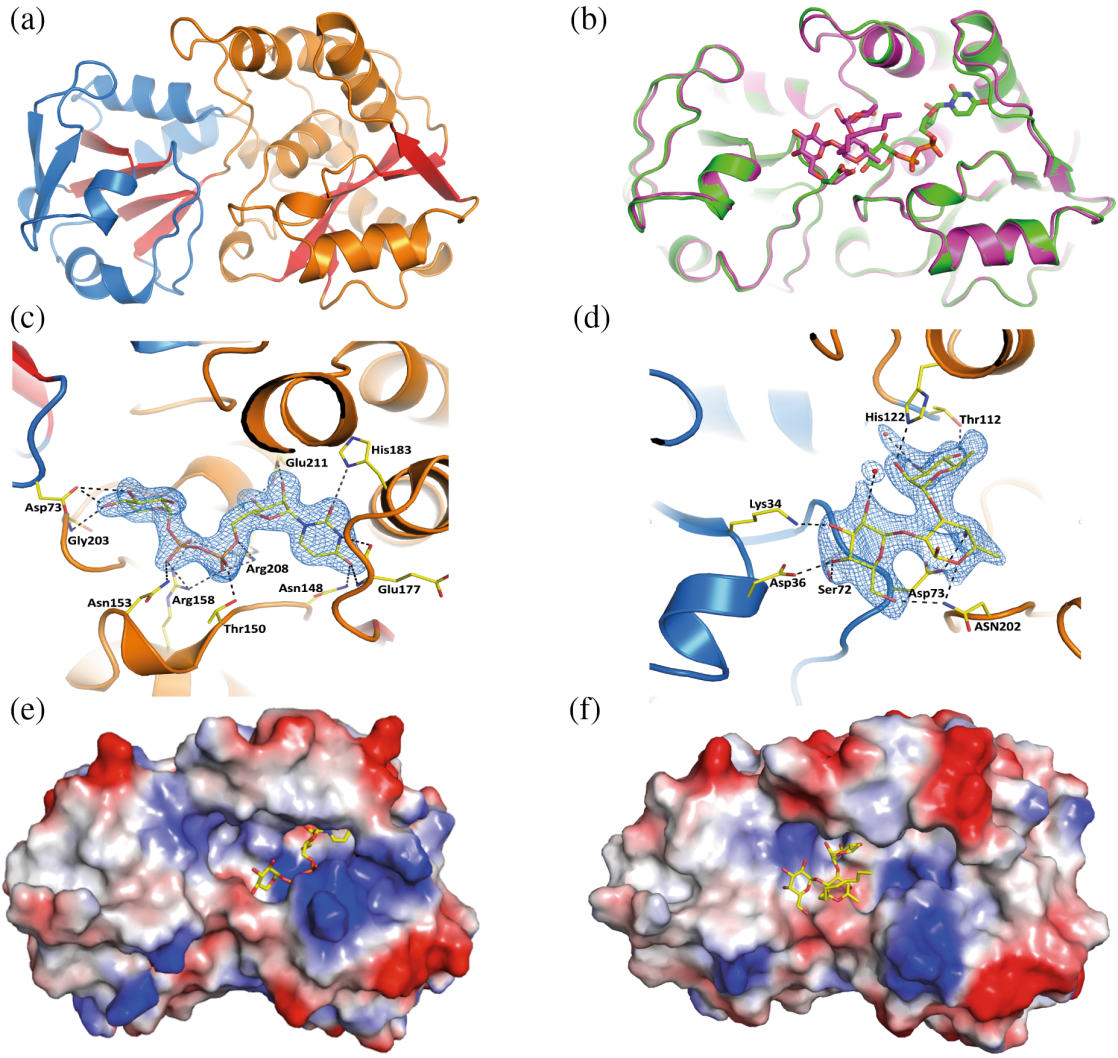
Schematic overview of the structural features of A075L. (a) Cartoon representation of the Rossmann fold‐like domains with the beta‐sheets colored in red. The N‐terminal domain is shown in blue, and the C‐terminal in orange. (b) Superposition of the crystal structures of A075L bound to the sugar donor **1** (in green, PDB: 8AVQ) and to the synthetic acceptor **6** (in magenta, PDB: 8Q8I). The ligands are shown in stick representation (c). View of the UDP‐α‐D‐Xyl donor **1** when bound to the C‐terminal domain. (d) View of the acceptor **6** when bound to the N‐terminal domain. In all cases, the ligands are shown in stick representation, the hydrogen bonds with the side chains of A075L are depicted as dashed lines, and the 2Fo‐Fc electron density maps contoured at 1.5 sigma are shown in blue. (e, f) Surface representation of A075L with the donor and acceptor molecules in stick representation. The surface is colored from red to blue, according to the electrostatic potential from −15 kBT to +15 kBT, where kB is the Boltzmann constant, and *T* is the Kelvin temperature.

To address the enzymatic mechanism, we solved the 3D structure of A075L in its *apo* form as well as in its complexes with the donor **1** and the trisaccharide **6** acceptor. Despite numerous attempts, the formation of a ternary complex between the enzyme, **1** and **6** was inaccessible. The structural analysis of these structures revealed that A075L adopts a GT‐B fold, comprising two Rossman‐like domains. The active site is located between these two domains; the N‐terminal domain is responsible for the glycan acceptor coordination, whereas the C‐terminal domain binds the UDP‐sugar donor (Figure 5a,b). The density map of UDP‐α‐D‐Xyl identified the sugar donor in a well‐defined pocket where Asn148 coordinates the uracil moiety, and Arg208 and Arg158 coordinate the phosphate groups. Among the 17 well‐defined direct hydrogen bonds with **1** (Figure 5c and Table 1), there are four H‐bonds between A075L and the nucleoside moiety and three specific H‐bonds with the Xyl moiety. Thus, A075L recognizes **1** with very high specificity.

**TABLE 1:** Summary of the H‐bonds found in the crystallographic structures between donor **1** and the trisaccharide **6** acceptor with the partner residues in A075L.

A075L residues	H‐bond distance to 1	
	Atom	Distance (Å)
**Asn148 ND2**	Base O4	3.03
Glu 177 N	Base O4	3.23
Glu 177 O	Base N3	3.06
His 183 ND1	Base O2	3.28
Glu211 OE2	Ribose O2	2.61
Glu211 OE1	Ribose O3	2.61
**Arg208 NH2**	Ribose O5	3.14
Thr150 OG1	Phosphate O2A	2.63
**Arg208NH2**	Phosphate O3A	2.85
**Arg208NH1**	Phosphate O1A	3.30
Arg158NH2	Phosphate O1A	2.89
Arg158NH1	Phosphate O1B	3.03
Arg158NH1	Phosphate O2B	3.06
Asn153ND2	Phosphate O2B	2.80
**Asp73OD1**	Xylose O3	3.29
**Asp73OD1**	Xylose O4	2.66
Gly203N	Xylose O4	3.02
	**H‐bond distance to 6**	
His122 NE2	Rhamnose O2	2.86
Thr112 OG1	Rhamnose O4	2.97
**Asp 73 OD1**	Fucose O4	2.64
Asn 202 ND2	Fucose O5	2.81
Asn 202 ND2	Galactose O6	2.89
Ser 72 OG	Galactose O4	2.71
Asp 36 OD2	Galactose O4	2.47
Lys 34 NZ	Galactose O3	3.01

*Note*: Residues that were mutated are in bold. O2 = OBF, O4 = OBB, O4 = OAT, O5 = OAP (labels in the PDB: **8Q8I**).

The crystal structure of A075L in complex with trisaccharide **6** shows a clear density map for the acceptor, including the octyl group (Figure S3a,b). The Rha moiety is located within a deep pocket and establishes hydrogen bonds with His122 and Thr112. The Gal ring engages in four hydrogen bonds with residues Asn202, Ser72, Asp36, and Lys34, and is also somewhat exposed to the solvent (Figure 5d and Table 1). Given its close proximity to the acceptor, Asp73 appears to be the best‐positioned residue to act as a catalytic base, facilitating the deprotonation of Fuc O4.

The key roles of the residues identified above were addressed by preparing specific site‐directed mutants of A075L and analyzing their binding features by STD‐NMR spectroscopy. First, a double mutant (N148A, R208A), targeting those enzyme residues involved in the interaction with the ribose, the bases, and the phosphate moieties of the donor, was analyzed. Only very weak STD signals were detected for the interaction with **1**, which exclusively involved protons of the aromatic uracil region. In contrast, significant STD signals were observed for the acceptor **6** (Figure S4a). These results provide support for the proposal that the side chains of Asn148 and Arg208 are key for the binding of the donor, but do not affect the binding of the trisaccharide acceptor.

Then, to test the possible function of Asp73 as the catalytic base, the D73A mutant was produced. Unfortunately, the expression test showed that the mutated protein was unstable, and its expression in *Escherichia coli* produced very low yield. Thus, we kept this construct fused to Glutathione S‐Transferase (GST) (GST–A075L_D73A_). STD‐NMR spectroscopic experiments showed that this mutant still bound both the acceptor and the donor, but the glycosylation reaction did not proceed (Figure S4b). Moreover, the observed STD NMR signals for **6** were significantly stronger in the presence than in the absence of **1**, but the reaction product was never detected. Furthermore, no hydrolysis or trans‐glycosylation activity was detected, even after 4 days.

As mentioned above, diverse crystallization experiments were performed in the presence of both the donor and the synthetic acceptors simultaneously to trap the product or an intermediate state. Despite extensive screening, only crystals with an empty active site or no crystals at all were obtained. To overcome the problem of the rapid reaction speed, the mutant A075L_D73N_ was cloned and produced. While the reaction was slowed, it took 2 days for the product to appear when this mutant was incubated with the trisaccharide acceptor **6** and donor **1** (Figure S5b,d,e). It was still insufficient to obtain the target complex in the crystal. In the other hand, a pentasaccharide acceptor α‐D‐Gal‐ (1 → 2) [α‐D‐Rha‐(1 → 3)]‐α‐L‐Fuc‐(1 → 3)‐[β‐D‐Xyl‐(1 → 4)]‐β‐D‐Glc‐Octyl (**7**), corresponding to the proposed natural substrate for the enzyme was also employed. MALDI mass spectrometry analysis showed that the reaction proceeded smoothly to give hexasaccharide **9** (Figure S5a,c). However, we did not obtain any crystals containing the pentasaccharide under the same or similar experimental conditions or by using a wider screening.

### 3.2 Molecular dynamics simulations

**FIGURE 6:**
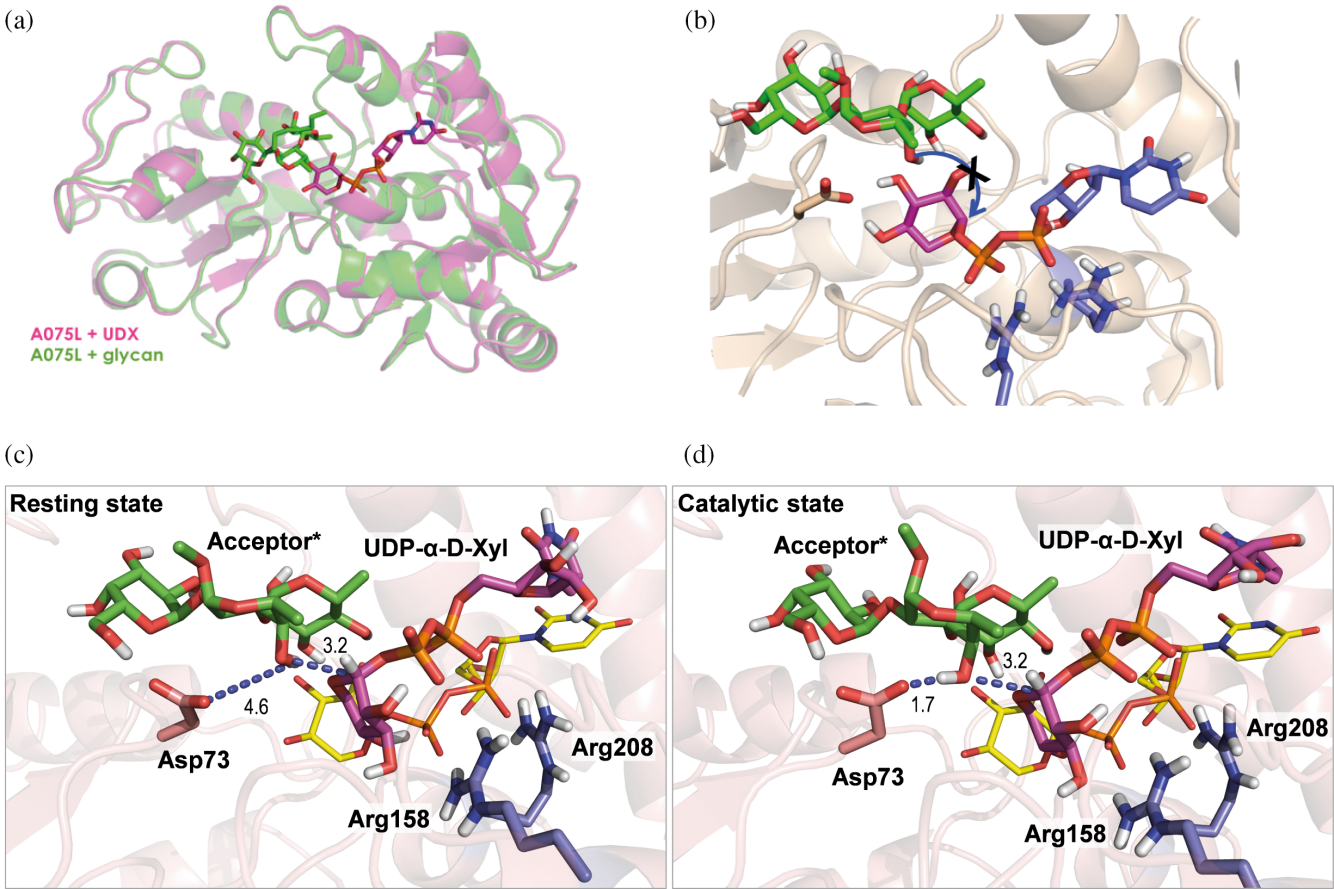
Towards deciphering the catalysis event: Snapshots from the MD simulations. (a) Cartoon representation of the superposition of the complexes A075L: donor (PDB: 8AVQ in pink) and A075L: acceptor (PDB: 8Q8I in green). (b) The blue arrow highlights the orientation required for the S_N_2‐like nucleophilic attack to take place. (c, d) Representative non‐catalytic and catalytic poses, respectively, derived from an unrestricted MD simulation of the A075L: **1**: **6** ternary complex. The catalytic residue Asp73 (in pale red), phosphate‐binding Arg158 and Arg208 (in blue), sugar donor (in magenta) and acceptor (in green) are shown as sticks. Non‐polar hydrogens have been omitted for clarity. The crystallographic binding pose of **1** (PDB ID 8AVQ) is also shown as yellow sticks as a reference.

The NMR results described above have shown that the transfer of the distal D‐Xyl proceeds with inversion at the anomeric carbon. Thus, the nucleophilic attack of the L‐Fuc C4 hydroxyl group should occur from the backside of the pyrophosphate leaving group (Figure 6b, blue arrow). Inspection of the X‐ray crystallographic structures revealed that such a geometric arrangement cannot be easily achieved from the observed conformation of the donor and acceptor in the complexes (Figure 6a). Hence, we hypothesized that a conformational rearrangement takes place for catalysis to occur.

Molecular dynamics (MD) simulations were then performed on a putative 3D‐structure of the ternary complex formed by A075L, donor **1** and the trisaccharide acceptor **6** (see Methods in Data S1 for further details). The starting coordinates for the simulations were obtained from the experimental crystallographic structures of the complex with the donor A075L:**1** (PD:8AVQ) and with the acceptor A075L:**6** (PDB:8Q8I) (Figure 6a). The solvent‐exposed octyl chain was truncated to a methyl group to simplify the MD calculations. Giving the large flexibility predicted for the substrates in preliminary MD simulations, we restrained key distances between: (i) the OD2 atom of Asp73 and H4O atom of L‐Fuc to 1.9–2.5 Å and, (ii) O4 of L‐Fuc and C1 of D‐Xyl to 3.3–3.9 Å (Figure S7). In this way, both the sugar donor and acceptor were maintained in a potentially reactive pose. Pleasingly, very early in this restrained MD simulation, and in agreement with the initial hypothesis, a flip of the donor was observed, which now displayed an adequate pre‐catalytic presentation for a S_N_2‐like attack from the acceptor with inversion of configuration. (Figure 6d). A snapshot of this trajectory (*t* = 382 ns) was then subjected to four independent unrestricted MD simulations allowing both the glycan donor and acceptor molecules to adopt fully relaxed geometries. This optimal catalytic disposition was maintained throughout most of the simulations (4 × 500 ns; Figure S8), although other transient poses in which the catalytic base did not interact with the reacting hydroxyl group of the sugar acceptor were also detected. Of note, in the “catalytic” pose, the C‐4 hydroxyl group of L‐Fuc in the trisaccharide acceptor is hydrogen‐bonded to the catalytic base Asp73, and perfectly oriented for its nucleophilic attack to C1 of donor **1** in a S_N_2‐like manner.

## 4 DISCUSSION

Analysis of the X‐ray crystallographic structure of A075L reveals that it possesses two Rossmann‐like fold domains. In contrast to its earlier classification as an exostosin (PF03016) within the GT47 family (Speciale et al. 2019), we show that the region spanning from V66 to K222 does not constitute an independent domain. Instead, this region comprises the β‐sheet of the N‐terminal Rossmann domain and several helices at the C‐terminal domain, as shown in Figure S3e. Additionally, the structure of A075L in complex with **6** (PDB:8Q8I) shows that the acceptor binds to the N‐terminal region, while **1** (PDB:8AVQ) is bound in the C‐terminal domain, categorizing this enzyme within the GT‐B fold class. ^1^H‐NMR spectroscopic analysis of the reaction of A075L with the sugar donor alone (resulting in its hydrolysis) showed the formation of the β‐xylose at early stages, suggesting the reaction proceeds with inversion of configuration. Indeed, in reactions in which the acceptor was added, the formation of the corresponding β‐linked tetrasaccharide was confirmed by the presence of a NOE cross‐peak between H1 Xyl and H4 Fuc, confirming the inversion mechanism of the reaction.

In light of these results, A075L has defined a new GT family, GT114 (www.cazy.org). This family has 178 homologs identified to date, primarily in viruses and bacteria, but also in a few eukaryotes, including fungi (*Chytriomyces* and *Rozella*), phytoplankton (*Chrysochromulina*), algae (*Chlamydomonas*), and diatoms (*Thalassiosira* and *Nitzschia*). The protein is widespread in other PBCV‐1 like viruses and Acanthocystis turfacea chlorella virus‐like genomes, which is not surprising as many of these have been shown to produce similar N‐glycans as PBCV‐1. The presence of homologous enzymes in these organisms suggest that N‐glycans with structures similar to those produced by PBCV‐1 may be present outside of giant viruses.

Further analysis by STD‐NMR spectroscopy demonstrated that the synthetic acceptors used in this study all interact with A075L through a groove‐type binding mode, where the entire ligand is embedded within the binding site. Nonetheless, a minimum acceptor size is required for the D‐Xyl transfer to take place. The enzyme cannot function as GT when only the L‐Fuc monosaccharide (**2**) or its octyl glycoside (**3**) are used as an acceptor substrate. In addition, the reaction only proceeds slowly with disaccharide **4** and not at all with disaccharide **5**, while it advances fast with a trisaccharide (**6**) or pentasaccharide (**7**) acceptors. Thus, it appears that the motif present in **4** (α‐D‐Rha‐(1 → 3)‐α‐L‐Fuc), is the minimum acceptor for the enzyme.

Analysis of the X‐Ray crystallographic structure of A075L bound to the trisaccharide provides an explanation for the observed specificity. The D‐Rha moiety is deeply inserted in the enzyme pocket, which, in turn, places the C4 hydroxyl group of the L‐Fuc moiety near Asp73. Deprotonation of this hydroxyl group by Asp73 facilitates nucleophilic attack on the donor. This exquisite arrangement highlights the specificity of the enzyme for a minimal acceptor motif. Moreover, the reaction is much slower with acceptor disaccharide **4** than with trisaccharide **6**. Indeed, the later reaction proceeds so fast that the acceptor cannot be detected at time zero, suggesting that the trisaccharide is likely the minimal motif required for its activity.

Through site‐directed mutagenesis, we demonstrated that the binding of the donor and the acceptor are independently regulated. Introduction of point mutations such as N148A and R208A affect the binding of the UDP‐xylose donor, but not the recognition of the acceptors. Nevertheless, the increase of the STD‐NMR signal of the acceptor in the presence of the donor suggests the existence of a synergistic effect for their binding to the enzyme. The fact that the A075L_D73N_ mutant slows the reaction, while the A075L_D73A_ mutant completely inhibits the reaction, strongly indicates that Asp73 functions as the catalytic base for deprotonating the Fuc moiety of the acceptor. The low yield expression of the A075L_D73A_ mutant was unexpected, but the structural analysis revealed that this residue connects the two domains through a hydrogen bond with Asn202 (Figure S3c,d). Mutation of Asp73 to Ala abolishes this interaction, likely leading to protein instability. The retention of the GST tag during purification became necessary to conduct activity assays with A075L_D73A_ mutant. Conversely, A075L_D73N_ was soluble, albeit less active than the wild type (Figure S5).

Despite several attempts to co‐crystalize A075L with both the acceptor and the donor molecules simultaneously, none were successful. The reaction occurred so rapidly that we consistently obtained the *apo* form without the bound product or intermediate, even when using the A075L_D73N_ mutant. To understand the details of the glycosyl transfer mechanism, the geometries of A075L in complex with the donor and acceptor were initially superimposed. However, inspection of the superimposed structures showed that the distance between O4 Fuc and C1 Xyl exceeded 3.0 Å, too far for the nucleophilic attack. Additionally, it was deduced that the orientation of the UDP‐α‐D‐Xyl donor with respect to the Fuc moiety in the acceptor was not the proper one for the nucleophilic attack to take place. Because the X‐ray crystallographic structures represent only static snapshots of each separate reactant in stable binding modes, MD simulations, of the ternary complex, were carried out to explore a wider range of the available conformational space. After a fast conformational change of the donor, a stable catalytic disposition was maintained, where the axial Fuc O4 group in the trisaccharide formed a hydrogen bond with catalytic base Asp73, positioning the acceptor for nucleophilic attack on C1 of UDP‐α‐D‐Xyl in a S_N_2‐like manner.

Numerous glycosyltransferases found in both eukaryotes and prokaryotes face challenges for large‐scale production as they are often single‐pass transmembrane proteins. Common hurdles in their production have been addressed using different biotechnological and molecular biology methods (Forrester et al. 2019; Jaroentomeechai et al. 2022; Liang et al. 2015; McArthur and Chen 2016), yet limitations persist. In this context, we herein present the structural and biochemical characterization of A075L, a soluble chlorovirus xylosyltransferase easily produced in *E. coli* and amenable to large‐scale purification. This approach overcomes key challenges in GT production. Beyond its relevance in chlorovirus biology, A075L may serve as valuable model for structure‐based rational design and structure‐guided directed evolution approaches in GTs, potentially advancing applications in glycan biosynthesis (Chen et al. 2023; McArthur and Chen 2016). In addition, this structural work provides a basis for additional investigations of homologous proteins in bacteria and eukaryotes.

## 5 MATERIALS AND METHODS

### 5.1 Protein production and purification

Recombinant A075L WT and A075L mutants were produced in *E. coli* BL21 Gold cells, whereas proteins labeled with Seleno‐l‐methionine (SeMet) were isolated from *E. coli* B834 (DE3). Growth occurred at 37°C until OD600 reached 0.6, followed by protein expression overnight at 18°C with 0.5 mM IPTG. Cells were lysed at 4°C using high‐pressure homogenization and GST‐tagged proteins were purified via affinity chromatography. Proteins were eluted with 40 mM GSH, dialysed in cleavage buffer with PreScission protease, and further purified using a Q HP anion exchange column. Eluted proteins were pooled, concentrated, and stored at −80°C for future NMR, ITC, and crystallization experiments.

### 5.2 Isothermal titration calorimetry

Isothermal titration calorimetry (ITC) experiments were performed on a VP‐ITC microcalorimeter (MicroCal/GE Healthcare) at 25°C. Purified A075L WT protein was dialyzed overnight at 4°C in 20 mM HEPES pH 7.5, 150 mM NaCl, and 0.5 mM TCEP, then degassed before titration. ITC measurements used 55 μM protein and 1 mM UDP‐α‐D‐Xyl, with 17 injections of 10 μL and a 360 s interval between injections. Experiments were done in triplicate, data were fitted to a one‐site model, and corrections for heat of dilution were applied. Results were averaged, with standard errors calculated.

### 5.3 Nuclear magnetic resonance

All nuclear magnetic resonance (NMR) experiments were conducted at 25°C using a Bruker AVANCE 2600 MHz spectrometer with a triple‐channel probe. Compound 1H‐NMR resonances were assigned through standard TOCSY, NOESY, and HSQC experiments. Samples were prepared in D2O for analysis. Enzyme reactivity was monitored by 1H‐NMR spectroscopy with reaction mixtures containing acceptor substrate, UDP‐D‐Xyl, and A075L in phosphate‐buffered saline. STD‐NMR spectroscopy was performed for samples with varying ratios of UDP‐D‐Xyl/A075L. Protein saturation was achieved with a train of 50 ms Gaussian‐shaped pulses with a total saturation time of 3 s. Blank STD experiments were performed to rule out direct irradiation effects. NOESY experiments used a 250 ms mixing time and were performed with UDP‐Xyl, trisaccharide 6, and A075L in phosphate‐buffered saline.

### 5.4 Crystallization, data collection, and structure determination

A075L WT and SeMet derivatives were crystallized using vapor diffusion methods, with rectangular box‐shaped crystals forming after 3–4 days at 18°C in MORPHEUS condition 1–12. This condition was refined, yielding good crystals by mixing 1 μL of 9 mg/mL protein with a precipitant solution. Complex crystals were obtained by co‐crystallization with UDP‐α‐D‐Xyl or acceptor **6**. Crystals were cryoprotected with 20% glycerol and flash‐frozen. Diffraction data were collected at the I24 at the Diamond light source (UK) and XALOC at the ALBA light source (Spain). The A075L WT structure was solved by SAD at 2.18 Å resolution and refined using PHENIX (Liebschner et al. 2019) and COOT (Emsley et al. 2010), resulting in a final structure with a Rfac of 22.4% and a Rfree of 27.8%. Data collection statistics for each dataset are shown in Table S1.

## AUTHOR CONTRIBUTIONS

**Maria Elena Laugieri:** Investigation; writing – original draft; writing – review and editing. **Immacolata Speciale:** Investigation. **Ana Gimeno:** Investigation; writing – original draft; writing – review and editing. **Sicheng Lin:** Investigation. **Brock W. Byers:** Investigation. **Ana Poveda:** Investigation. **Reyes Núñez‐Franco:** Investigation. **Idoia Iturrioz:** Investigation. **María J. Moure:** Investigation. **Gonzalo Jiménez‐Osés:** Investigation; supervision. **Irene Russo‐Krauss:** Investigation. **Anna Notaro:** Investigation. **James L. Van Etten:** Writing – review and editing. **Todd L. Lowary:** Investigation; writing – review and editing; supervision; funding acquisition. **Jesús Jimenez‐Barbero:** Investigation; funding acquisition; writing – review and editing. **Cristina De Castro:** Conceptualization; investigation; writing – review and editing; supervision. **Michela Tonetti:** Conceptualization; investigation; writing – original draft; writing – review and editing; supervision; funding acquisition. **Adriana L. Rojas:** Conceptualization; investigation; writing – original draft; writing – review and editing; supervision.

## Supporting information


**Movie S1.** MD simulation movie. 360‐degree panoramic zoom of the active site in the catalytic sate derived from an unrestricted MD simulation of the A075L: **1**: **6** ternary complex. The catalytic residue Asp73 (in pale red), phosphate‐binding Arg158 and Arg208 (in blue), sugar donor (in magenta) and acceptor (in green) are shown as sticks. Relevant distances between the nucleophilic sugar acceptor, the electrophilic sugar donor, and the catalytic base are shown as slate gray and yellow dashed lines, respectively. Non‐polar hydrogens have been omitted for clarity.

**Movie S2.** A075L_Movie. Overview of the structural features of A075L. The N‐terminal domain is shown in blue, and the C‐terminal in orange. Superposition of the crystal structures of A075L bound to the sugar donor **1** (in green, PDB: 8AVQ) and to the synthetic acceptor **6** (in magenta, PDB: 8Q8I). The ligands are shown in stick representation, and the hydrogen bonds with the side chains of A075L are depicted as dashed lines.

**Data S1.** Supporting Information.
