# Supplementary material for: Unveiling the GT114 family: Structural characterization of A075L, a glycosyltransferase from Paramecium bursaria chlorella virus‐1 (PBCV‐1)
Source: Protein Sci. 2024 Nov 18;33(12):e5196. doi: 10.1002/pro.5196 (PMC11571054; doi:10.1002/pro.5196)
Supplement: Supplementary file 3 — Data S1. Supporting Information. [file PRO-33-e5196-s001.docx]

**Extended Material and Methods**

**Cloning**

A075L WT gene was amplified from PBCV-1 cDNA. Then, it was cloned in pGEX-6p1 expression vector using restriction enzymes (BamHI and XhoI). The A075L mutants; A075L N148A-R208A and A075L D73A were obtained using the Quikchange site-directed mutagenesis method (Agilent Technologies), following the manufacturer protocol, using the A075L WT vector as a template. The A075L D73N single mutant construct was synthesized by GenScript, using theA075L WT pGEX-6p1 vector as template. Primers used are listed in Supplementary Table 2:

**Protein production and purification.**

Recombinant A075L WT and A075L single mutant (D73N), were produced in *E. coli* (BL21 Gold cells). The cells grew at 37 °C until the OD600 was 0.6, and then protein expression was induced overnight at 18 °C with 0.5 mM IPTG (Isopropyl-β-D-1-thiogalactopyranoside). Cells were harvested by centrifugation, resuspended in PBS and lysed at 4 °C by high-pressure homogenisation (27 Kpsi). GST-tagged proteins were purified by affinity chromatography using GSH-Sepharose 4B resin (GE Healthcare) packed in a gravity column. After extensive washing with the same loading buffer, proteins were eluted by adding 40 mM GSH in PBS. The resin was incubated at room temperature for 20 min, mixing the resin every 5 min. Then, the flow-through was collected and dialysed in the cleavage buffer (50 mM Tris-HCl, 150 mM NaCl, 1mM DTT) overnight with a 2U/100 ug protease/protein to cleave the GST-tag. The day after, recombinant proteins released from GST were incubated in batch with GSH Sepharose beads for 2 h at 4 °C, using gentle agitation, to capture the cleaved GST and the PreScission protease. The beads were then transferred in a gravity column (BioRad), and the flow-through containing the protein was collected. This procedure was performed twice. Then the protein solution was diluted to 50mM Nacl and then loaded in a 5ml Q HP (GE Healtcare) anion exchange column using as buffer A; 50 mM Tris-HCl, pH 7.5, 1 mM DTT in the AKTA FPLC system. Elution was carried out with a gradient from 0% to 50% of Q buffer B (1M NaCl, 50 mM Tris-HCl pH 7.5, 1 mM DTT) in 10 CV (column volumes). The protein was eluted at 200 mS, and positive fractions were pooled and concentrated using a centrifugal Millipore filter device (cut-off 10 kDa). Concentrated A075L WT and A075L D73N were stored at –80 °C for later use in NMR spectroscopic, ITC and crystallisation experiments.

SeMet A075L.

Selenomethionine (SeMet)-substituted A075L was produced in *E. coli* B834 (Strata-gene), a methionine auxotroph strain. Cells were initially grown in LB media, then recovered by centrifugation (at OD600 = 0.6) and washed twice with ice-cold PBS. Then, the pellet was resuspended in the Base medium plus nutrient mix from Molecular Dimensions at 37 °C for 1 h. Protein expression was induced with 0.5 mM IPTG overnight at 18 °C with the addition of 40 µg/ml Se-Met (Sigma). SeMet A075L purification was carried out under the same conditions as the native protein. Using MALDI-TOF mass spectrometry, the efficiency of SeMet incorporation was evaluated by mass differences between native (unlabeled) and SeMet-labeled protein samples. The observed mass differences confirmed 100% selenomethionine incorporation.

A075L double mutant (N148A, R208A)

The Q-positive fractions of the mutants display less purity than the WT protein. Thus, they were pooled and concentrated to1 ml and applied to a gel filtration column (Superdex 200 10/30). After gel filtration, some GST contaminant was present. The protein was incubated again with the GSH Sepharose resin twice, for 1 h at 4 °C and checked by SDS PAGE. The last flow-through was collected and concentrated to 7.2 mg/ml and stored at –80 °C for later use in the experiments.

A075L single mutant (D73A).

This construct presented solubility problems after GST cleavage. Thus, we decided to keep it as a fusion protein with GST. After the affinity column, GST–A075L_D73A_ was dialysed overnight against Tris-HCl 50 mM, NaCl 150 mM pH 7.5. The next day, the protein was diluted to 50 mM NaCl with Q buffer A (50 mM Tris-HCl, pH 7.5, 1 mM DTT) and then loaded on a 5 ml Q HP (GE Healthcare) anion exchange column using the AKTA FPLC system. Elution was carried out with a gradient of Q buffer B (1M NaCl, 50 mM Tris-HCl pH 7.5, 1 mM DTT). The protein was eluted at 30 mS, and positive fractions were pooled together, checked by SDS PAGE, and concentrated and applied to a gel filtration column (Superdex 200 10/30) in 50 mM Tris-HCl, 300 mM NaCl pH 7.5. The protein eluted in a broad peak from 7.7 ml to 15 ml. SDS PAGE checked fractions were incubated overnight at room temperature with a molar ratio of 1:1 UDP-α-D-Xyl, to stabilise the protein. The day after, insoluble material was removed by centrifugation (10 min at 4500 rpm). Then, the protein solution was diluted 50mM NaCl to inject it again into the Q column. Protein elutes at 31.8 mS/cm in a single peak. SDS PAGE positive fractions were concentrated until 0.3 mg/ml and checked by MALDI TOF. Concentrated GST–A075L_D73A_ was used for STD experiments.

**ITC.**

Isothermal titration calorimetry (ITC) experiments were carried out on a VP-ITC titration microcalorimeter (MicroCal/GE Healthcare) at 25 °C. A075L WT purified protein was dialysed overnight at 4 °C against 20 mM HEPES pH 7.5, 150 mM NaCl and 0.5 mM TCEP and degassed for 5 min in a ThermoVac sample degasser before titration. ITC measurements of A075L WT with UDP-α-D-Xyl were collected using 55µM of protein in the cell and 1mM of UDP-α-D-Xyl in the syringe. Each experiment involved one initial 2 µL injection (not used in data fitting), followed by 17 injections of 10 µl aliquots with 360 s between injections. The cell temperature was set to 25 °C with a stirring speed of 750 r.p.m. This experiment was repeated in triplicate, and the resulting titration data were integrated and fitted to a one-site model using the Origin ITC software package supplied by MicroCal. Values were averaged, and standard errors were calculated based on a near 1:1 restricted binding stoichiometry. Similar injections of UDP-α-D-Xyl in the syringe and buffer in the cell were performed to determine the heat of dilution used to correct the experimental data.

**Enzymatic reactions**

All reactions were performed at 25 °C in PBS buffer (phosphate buffer saline, Sigma Aldrich product number P4417) using the soluble enzyme solution (17 mg/ml), UDP-α-D-Xylose (**1**), purchased from Carbosynth, and three acceptor substrates as detailed in Supplementary Table 3. Reactions were monitored via HPLC, as described previously^4^. Briefly, a C18 resin column eluted with a 70 % of methanol, at a flow of 0.5 mL/min, was used, injecting 10 μl of the crude reaction solution. Four reactions were set up as listed in the Supplementary Table 3. The crude reactions were purified via Sep-Pak C18 cartridge^1^, to isolate the octyl glycosides, which were then analysed via NMR spectroscopy.

**Product purification for crystallization experiments**

For the co-crystallization experiments of A075L with the product, the reaction of 0.74mM of acceptor **6** and 1.25uM of donor **1** in presence of 30uM of A075L was set up at 25C. Then, product **8** was purified by Gel filtration chromatography using a column (75 x 1.5 cm) packed with Bio-Gel P-2 media (BIO-RAD) and characterized by MALDI and NMR (SFigure 2A-C). Unfortunately, the crystals did not grow.

**NMR Experiments.**

*General Remarks.* All NMR experiments were performed at 25 ºC on a Bruker AVANCE 2 600 MHz spectrometer equipped with standard triple-channel probe (600 MHz). The ^1^H-NMR resonances of the compounds were assigned through standard TOCSY (60 and 90 ms mixing times), NOESY (50–500 ms mixing times), and HSQC experiments. 500 µL samples were prepared by dissolving the purified compound in D_2_O.

*^1^H-NMR spectroscopic analysis of enzyme reactivity.* Stock samples of donor and/or acceptor substrates were prepared in phosphate-buffered saline (10 mM sodium phosphate, 150 mM NaCl, pH = 7.4). A final mixture of acceptor substrate (0.75 mM), UDP-D-Xyl (1.25mM), and A075L (1 mg/ml) was prepared in phosphate-buffered saline (10 mM sodium phosphate, 150 mM NaCl, pH = 7.4). The reaction mixture was incubated at room temperature and monitored by ^1^H NMR spectroscopy.

*STD-NMR Spectroscopy.* Samples were prepared in phosphate-buffered saline (10 mM sodium phosphate, 150 mM NaCl, pH = 7.4) using UDP-D-Xyl/A075L or sugar/A075L ratios varying from 1:20 to 1:40 with A075L concentrations ranging from 20 to 45 μM. Representative experiments with significant STD responses are presented in the figures. In all cases, the on-resonance frequency was set at the aliphatic region (0.42 ppm) and the off-resonance frequency at 100 ppm. Protein saturation was achieved by using a train of 50 ms Gaussian-shaped pulses with a total saturation time of the protein of 3s in a 600 MHz spectrometer. A spin-lock filter (100 ms) was used to remove the background NMR signals of the macromolecule. Proton signals with the strongest STD effect were used as reference and relative STD intensities were calculated for clearly identified non-overlapped protons. To rule out direct irradiation effects, blank STD experiments in the absence of the protein were acquired for the different compounds and were subtracted from the STD spectra obtained in the presence of the enzyme.

*NOESY Experiments.* Samples were prepared in phosphate-buffered saline (10 mM sodium phosphate, 150 mM NaCl, pH = 7.4) using UDP-Xyl (1.25 mM), trisaccharide **6** (0.75 mM) and A075L (1 mg/ml). NOESY experiments were acquired using a phase-sensitive pulse program with gradient pulses in the mixing time. A mixing time of 250 ms was used for acquiring NOESY spectra.

**Crystallisation, data collection and structure determination**

A075L WT and SeMet derivatives were crystallized using vapour diffusion methods. Upon evaluation of numerous crystallization screens from various commercial sources, rectangular box shape crystals were obtained after 3–4 days at 18 ºC in the MORPHEUS condition 1–12 (Molecular Dimensions). This crystallization condition was further refined by employing the sitting-drop method and bigger volumes. Good crystals were obtained by mixing 1 µl of the protein at 9 mg/ml with 1 µl of the precipitant solution containing 0.06 M MgCl_2_, 0.06 M CaCl_2_, 37.5% MPD, P1K, PEG 3350, 0.1 M TRIS-Bicine pH 8.9. The crystals of the complexes of A075L with the donor and the acceptor were obtained in the same conditions by co-crystallization. For the complexes, the protein at 0.2 mM was incubated with 4 mM of UDP-α-D-Xyl (**1**) and/or 2 mM of the acceptor **6**, for 1 h. The crystals were cryoprotected by quick-soaking into mother liquor supplemented with 20%(v/v) glycerol before being flash-frozen in liquid nitrogen.

Diffraction data were collected at the I24 beamline at the Diamond light source (UK) and XALOC at the ALBA light source (Spain) using a Pilatus 6M detector. Diffraction images were indexed, integrated and scaled using XDS^2^. The A075L WT structure was solved by Single Anomalous Dispersion (SAD) in P1 space group at 2.18 Å resolution. Se atoms positions were identified using Hyss as implemented in Phenix and phasing was carried out with AutoSol, which gave a starting map into which 14 chains with 1901 residues were automatically placed with AutoBuild^24^. Next, iterative refinement with PHENIX and manual building in COOT^25^ yields a final model with eight molecules in the asymmetric unit (eight chains with 2240 residues). This model was used as a template for molecular replacement with the native dataset that diffracted to 2.0 Å resolution. The final structure has a R_fac_ and a R_free_ of 22.4% and 27.8%, respectively. Data collection statistics for each dataset are shown in Supplementary Table 1.

**Molecular Dynamics Simulations**

Simulations were carried out with AMBER 20 package^3^ implemented with ff14SB^4^, general Amber force field (GAFF2)^5^ and GLYCAM 06j-1^6^ force fields for the protein, the cofactor and the carbohydrate ligand, respectively. Parameters for the donor UDP-α-D-Xyl were generated by combining GLYCAM 06j-1 and GAFF2 force fields. Binding-site histidine residues (H74, H125 and H210) were modelled in their Nδ1-H tautomeric state (residue name HID in Amber). Initial structures were neutralised with either Na^+^ or Cl^–^ ions and set at the centre of a cubic TIP3P^7^ water box with a buffering distance between solute and box of 10 Å. For unrestricted simulations, a two-stage geometry optimisation approach was performed. The first stage minimises only the positions of solvent molecules and ions, and the second stage is an unrestrained minimization of all the atoms in the simulation cell. The system was then heated by incrementing the temperature from 0 to 300 K under the constant pressure of 1 atm and periodic boundary conditions. Harmonic restraints of 10 kcal mol^-1^ were applied to the solute, and the Andersen temperature coupling scheme^8^ was used to control and equalise the temperature. The time step was kept at 1 fs during the heating stages, allowing potential inhomogeneities to self-adjust. Water molecules were treated with the SHAKE algorithm^9^ such that the angle between the hydrogen atoms is kept fixed through the simulations. Long-range electrostatic effects were modelled using the particle mesh Ewald method^10^. An 8 Å cut-off was applied to Lennard-Jones interactions. The system was equilibrated for 2 ns with a 2 fs time step at a constant volume and temperature of 300 K.

For restricted simulations, a very similar protocol was used, but starting with a one-step geometry optimisation. All atoms were allowed to move except those directly involved in the glycosylation reaction. Thus, the distances between atoms OD2(Asp73)–H4O(Fuc) and atoms O4(Fuc)–C1(Xyl) were restricted to 1.9–2.5 Å and 3.3–3.9 Å, respectively, by imposing harmonic restraints of 10 kcal mol^-1^. Then the system was heated and equilibrated under the same conditions described above while maintaining the distance restraints. After equilibration, production trajectories were run under the same simulation conditions (500 ns in the case of the restricted simulation, and four independent replicas of 500 ns each for the final unrestricted simulation).

**
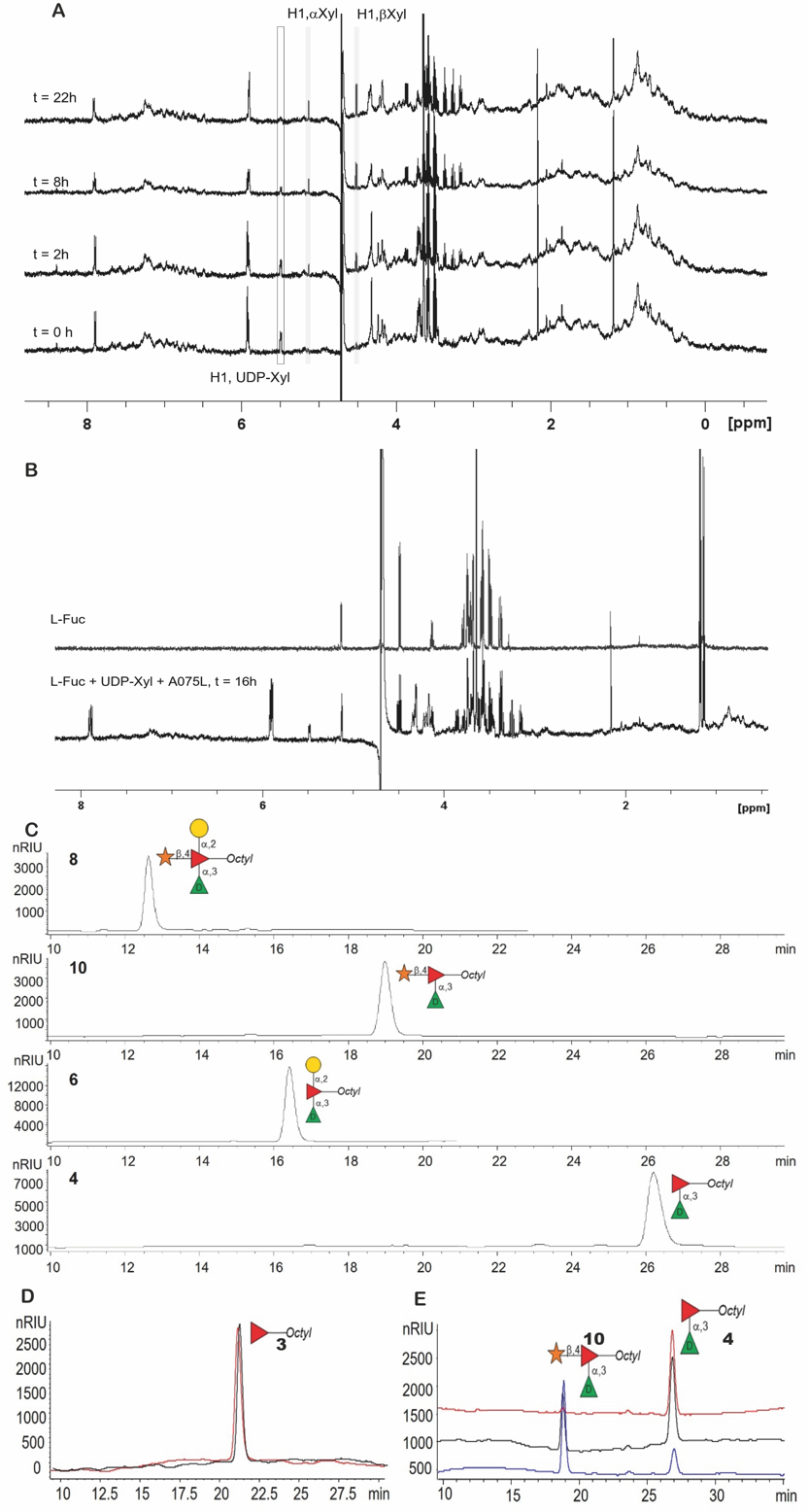
**

**SFigure 1**.^1^ Specificity of A075L as determined by NMR spectroscopy and HPLC. **A**. 600 MHz ^1^H NMR spectra of the reaction between A075L and **1** recorded at t = 0, 2 h, 8 h and 22 h. The H1 resonances protons for UDP-α-D-Xyl (**1**), α-D-Xyl and β-D-Xyl are highlighted. **B.** 600 MHz ^1^H NMR spectra of the enzymatic reaction product when L-Fuc (**2**) was used as a possible acceptor. The spectra show UDP-α-D-Xyl (**1**), L-Fuc (**2**) and D-Xyl-OH (both α and β-anomers), the latter coming from the hydrolysis of **1**. **C**. Chromatographic profiles of the substrates used as standards. **D.** Chromatographic profile of octyl α-L-fucoside (**3**) with A075L and **1** at the start of the reaction (red trace) and after overnight (~ 18 hrs) incubation (black trace), demonstrating no product had formed and that **3** is not an acceptor substrate for the enzyme **E.** Chromatographic profile of the reaction between disaccharide **4** and **1** in the presence of A075L at time = 2, 16 and 24 hrs (red, black and blue traces, respectively). The structure of the product, trisaccharide **10,** was confirmed by purifying and analyzing it via NMR.


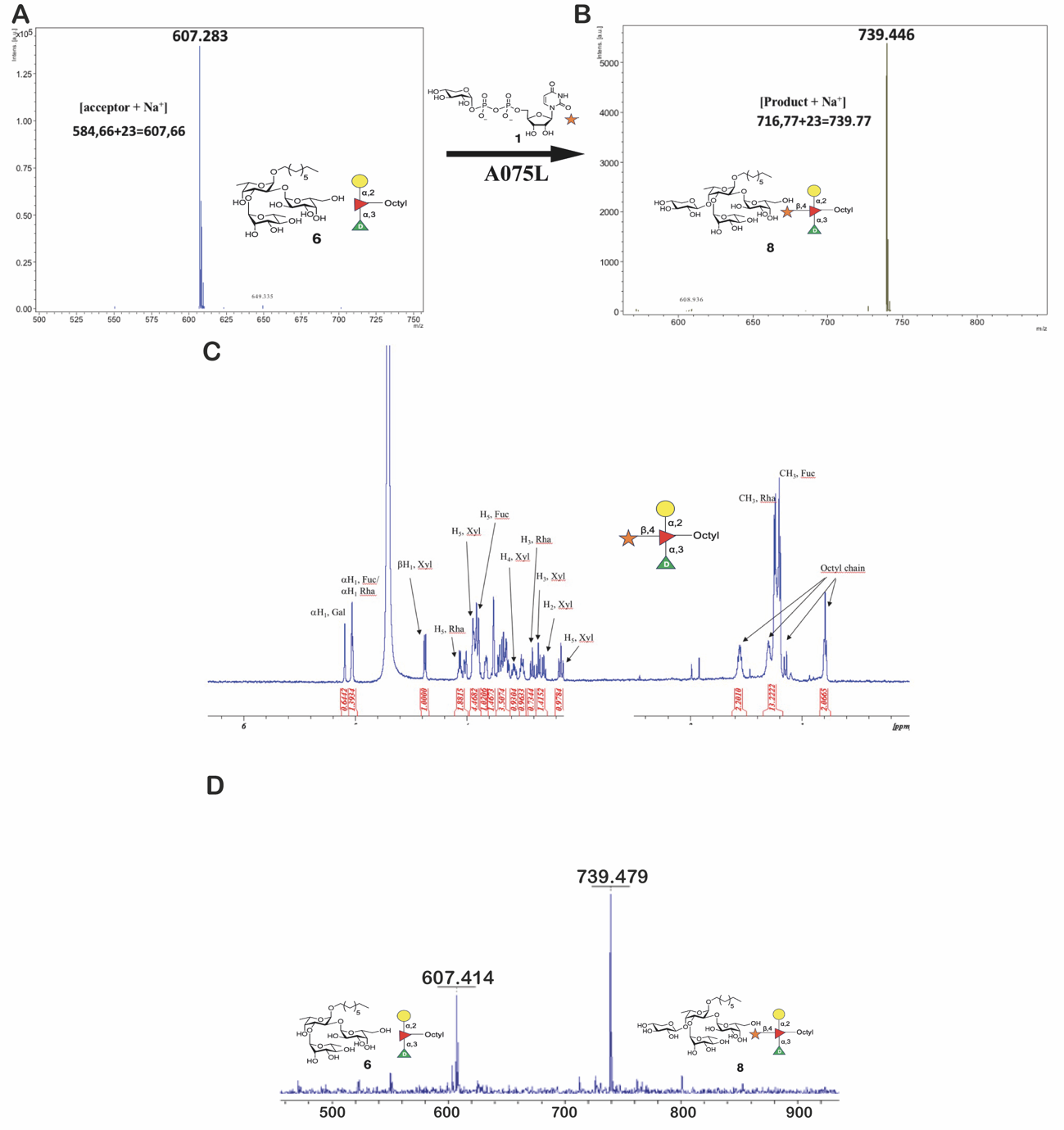


**SFigure 2.** Product characterization of A075L-catalyzed xylosylation of **6** using **1**. **A.** MALDI mass spectrum of **6**. **B**. MALDI mass spectrum the product **8** after the enzymatic reaction. **C**. ^1^H NMR spectrum of purified **8**. **D.**


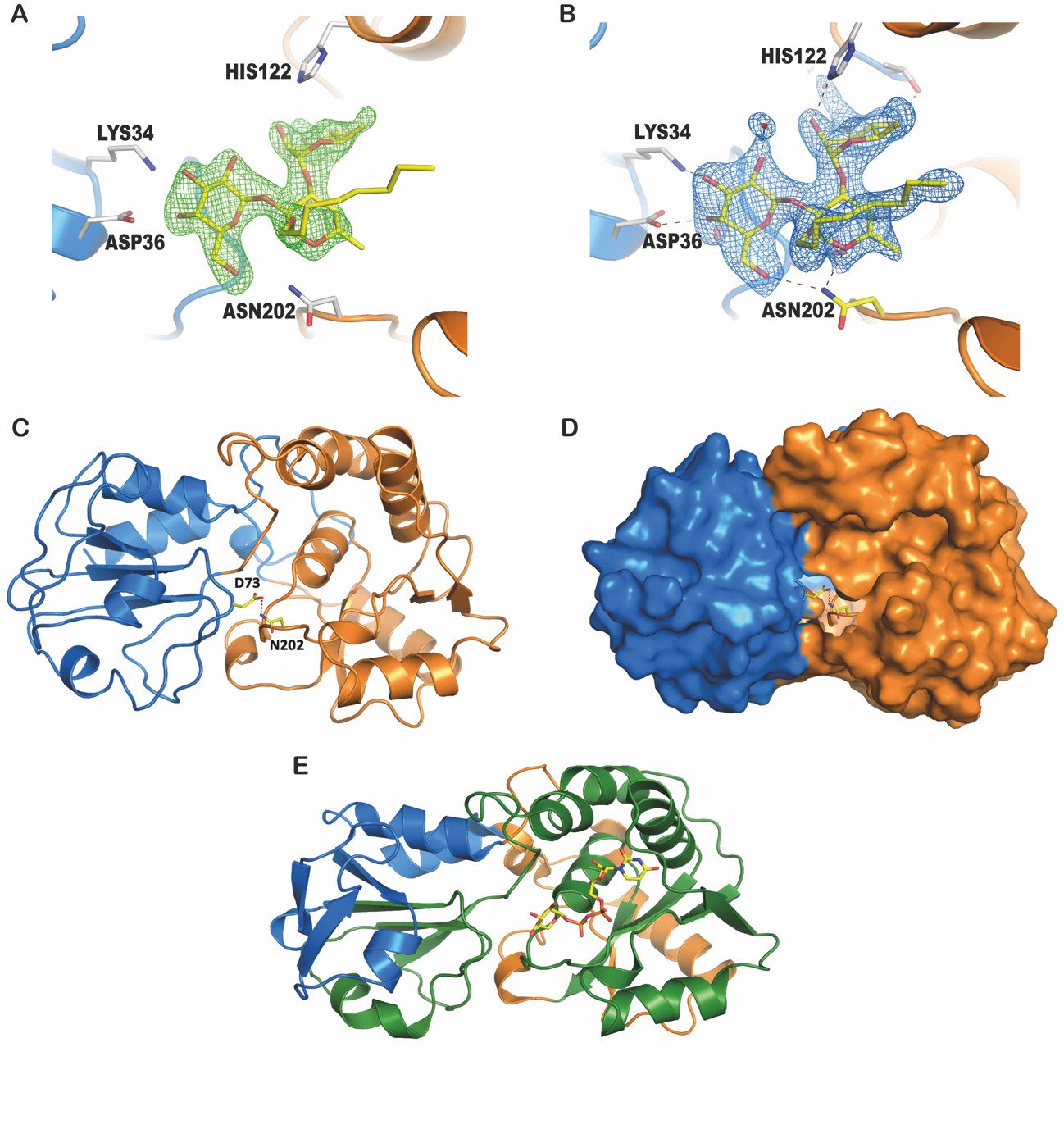


**SFigure 3.** Density map of the acceptor and connection of the two domains. **A**. Acceptor **6** bound to the N-terminal domain is shown in stick representation, the hydrogen bonds with the side chains of A075L are depicted as dashed lines and the Fo-Fc electron density maps contoured at 3.0 sigma are shown in green. **B**. The 2Fo-Fc electron density map contoured at 1.5 sigma is shown in blue, and the hydrogen bonds of the acceptor **6** with the side chains of A075L are depicted as dashed lines **C.** The hydrogen bond between Asp73 and Asn202, which serves as a link between these two domains, shown as black dashed lines. **D**. The two domains are presented in a surface representation. **E.** GT47 classification. Cartoon representation of the A075L structure with the N-terminal domain in blue, the C-terminal in orange and the region that was considered as exostosin domain in green.


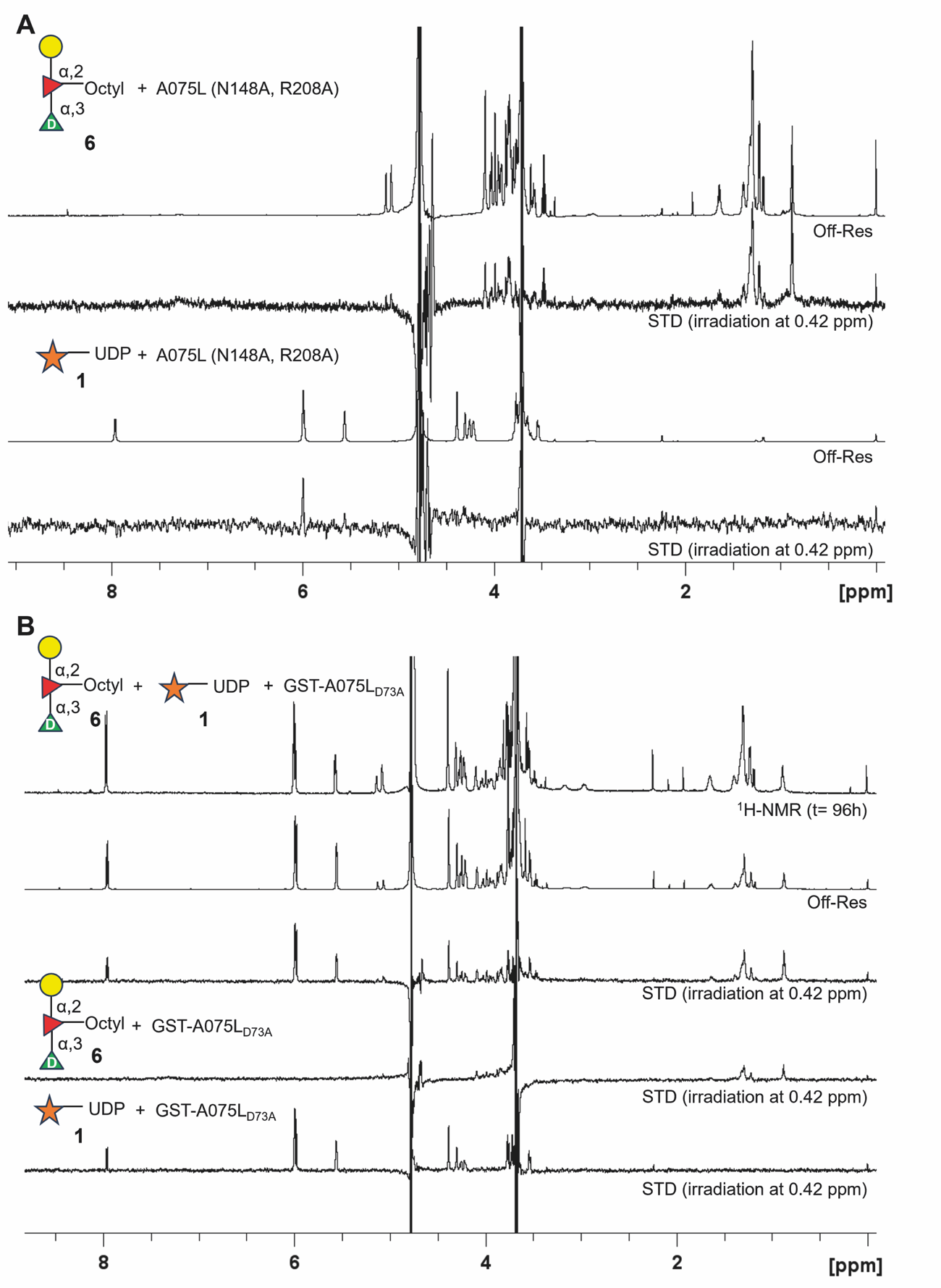


**SFigure 4.** STD NMR spectra for donor **1** and acceptor trisaccharide **6** with A075L mutants **A**. The mixture of **1** and **6** in presence of the A075L double mutant (N148A, R208A). **B.** The mixture of **1** and **6** in the presence of the GST–A075L_D73A_ mutant.

**
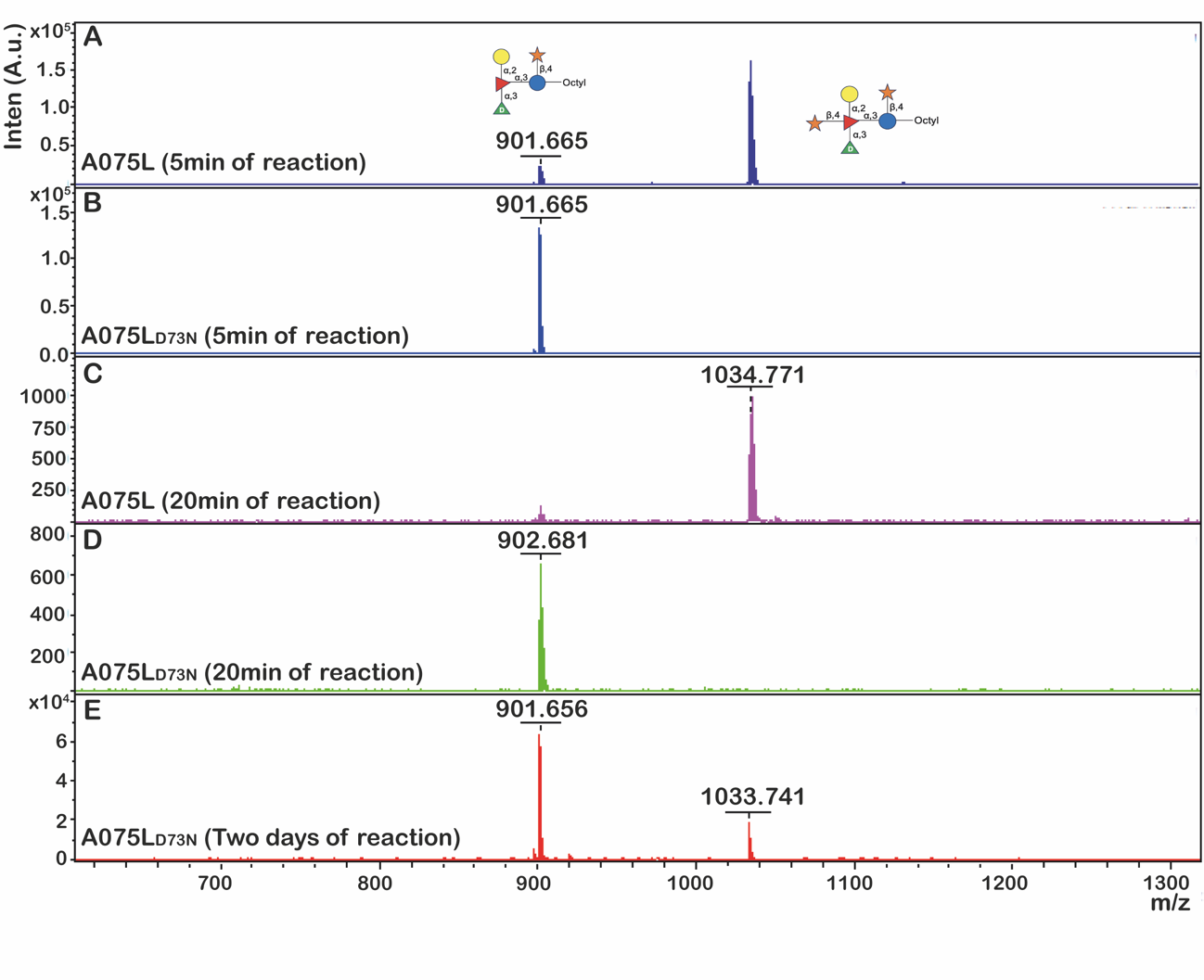
**

**Sfigure 5**. A075L and A075L_D73N_ enzymatic reactions. MALDI mass spectrum of **A. B** the product after 5 minutes of the enzymatic reaction of the acceptor **7** and donor **1** in the presence of A075L and A075L_D73N_, respectively. **C. D.** The same in A and B after 20 minutes. **E.** The reaction of the acceptor **7** and donor **1** in presence of the A075L_D73N_ after

two days.

**The dependence of the enzymatic activity on the presence of metal ions**

A075L enzymatic activity's dependence on metal ions was investigated by monitoring the reaction of acceptor **6** and donor **1** in the presence of EDTA by HPLC. The aim was to determine if the presence of the DYD motif (D73Y74D75) on A075L (SFigure 6A), implies the necessity of divalent metal ions for enzymatic activity. The reaction progressed similarly to its absence (SFigure 6B), suggesting the enzyme operates independently of metal ions.


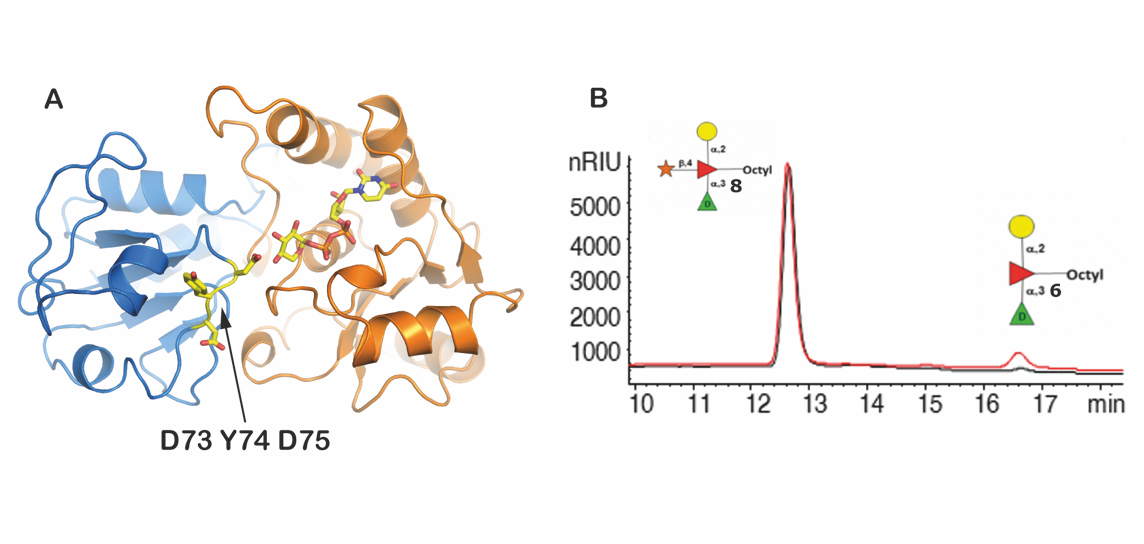


**SFigure 6.** Role of metal binding on catalysis. **A**. Cartoon representation of A075L with the DYD motif and the sugar donor in stick representation. **B.** HPLC chromatographic profiles of the reaction of A075L with acceptor **6** and donor **1**. Red: minutes after start, black: after two hours. The experiments were carried out in the presence of both Mn^2+^ and Mg^2+^cations after incubation with EDTA.


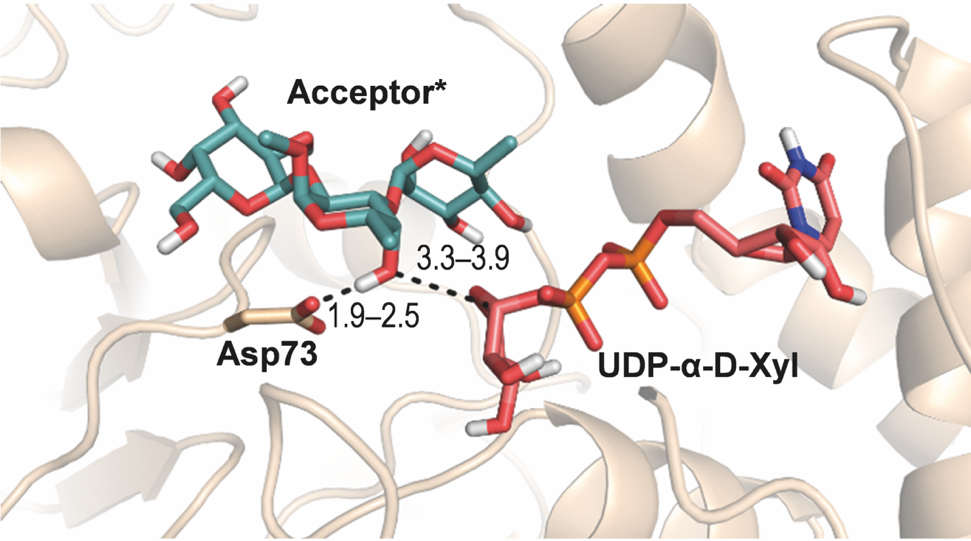


**Sfigure 7.** Distance restrictions (in angstrom) imposed in the initial MD simulation of the A075L: UDP-α-D-Xyl:α-D-Gal-(1→2)[α-D-Rha-(1→3)]-α-L-Fuc-(1→Me) ternary complex.


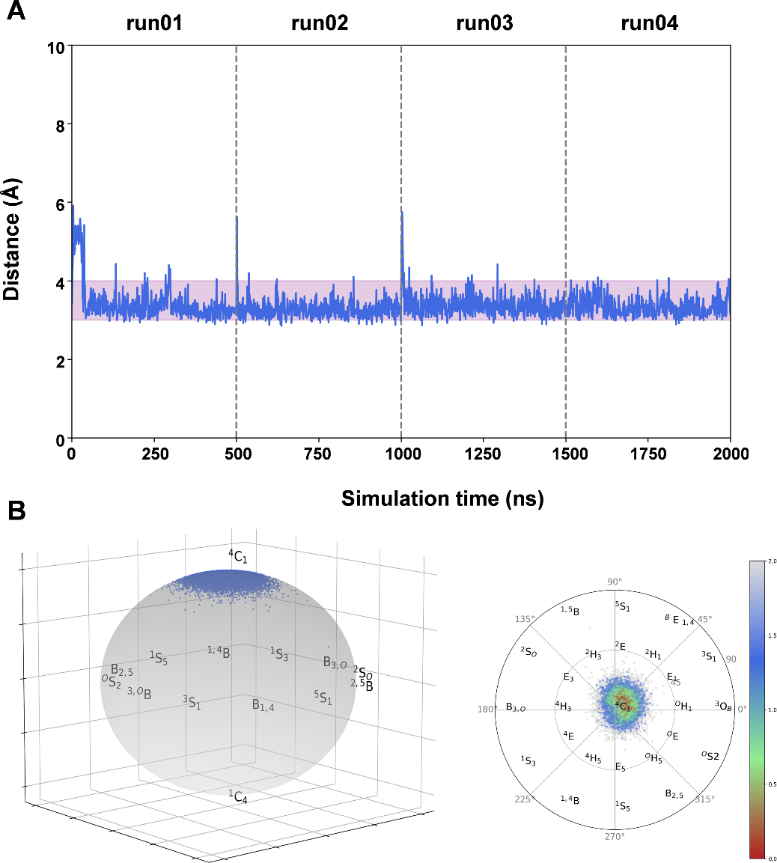


**SFigure 8.** Unrestricted MD simulation of the A075L: **1**: **6** ternary complex. **A**. Distance between the reactive hydroxyl group of the sugar acceptor and the anomeric carbon of the sugar donor along four independent 500 ns trajectories (aggregated time is shown in the X axis for clarity). The highly conserved value of this distance between 3-4 Å indicates a preorganized near-attack conformation between the two reaction partners. **B**. Aggregated Cremer-Pople parameters representing the puckering coordinate system of the six-membered pyranose ring in UDP-α-D-Xyl (left: spherical polar representation; right: projection of the sphere from the North Pole). The canonical ^4^C_1_ chair is maintained throughout the simulations.

**Supplementary Table 1**. Data collection and refinement statistics

|  | **A075L**  **SeMet**** | **A075L in complex with 1** | | **A075L in complex with 6** | |
| --- | --- | --- | --- | --- | --- |
| **Data Collection** |  |  | |  | |
| Wavelength [Å] | 0.9193 | 0.9795 | | 0.9791 | |
| Space group | P1 | C2 | | C2 | |
| Resolution [Å] | 50-2.18(2.31-2.18)* | 50-2.0(2.12-2.0)* | | 62.9-1.77(2.11-1.77)* | |
| Cell dimensions  *a, b, c* [Å]  α, β, γ [º] | 86.90 91.40 91.90  90.4 108.6 109.8 | 129.29 130.43 87.14  90.0 118.0 90.0 | | 126.579 50.456 51.543  90.0 96.32 90.0 | |
| CC_1/2_ (%) | 99.6(77.3) | 99.7(42.5) | | 98.6(62.6) | |
| Completeness (%) | 92.1(86.1) | 99.4(98.4) | | 85.2(43.7) | |
| I/σ | 9.84(2.36) | 10.6(1.23) | | 5.4(1.8) | |
| Number of unique reflexions | 237952 | 169521 | | 12052 | |
| Redundancy | 1.79(1.77) | 3.53(3.36) | | 4.9(2.0) | |
|  |  |  | |  | |
| **Refinement** |  | |  | |  |
| R-factor (%) | 19.37 | 19.60 | | 22.16 | |
| R-free (%) | 24.09 | 21.85 | | 27.06 | |
| No. atoms |  |  | |  | |
| Waters | 1144 | 782 | | 95 | |
| Ions: Mg/Ca | 25/26 | 39/13 | | 5/ 2 | |
| Ligand  Gol/EDO/BCN | 14/30/9 | 4  1/16/4 | | 1  1/8/1 | |
| R.m.s deviations |  |  | |  | |
| Bond lengths (Å) | 0.003 | 0.003 | | 0.002 | |
| Bond angles (º) | 1.244 | 1.172 | | 0.468 | |
| **PDB CODE** | **8ASA** | **8AVQ** | | **8Q8I** | |

*Highest resolution shell is shown in parenthesis.

** SeMet correspond to Selenomethionine labeled protein.

**Supplementary Table 2.** Primers used in this study.

| **Construct** | Primer 5´to 3´ | Primer 3´to 5´ |
| --- | --- | --- |
| **A075L_WT** | AATTGGATCCATGAAGCTCGCCGAACTTAC | AATTCTCGAGTTACTGACTATATTCGAGAA |
| **A075L_R148A** | CCGGGAAATTCTTGACTGTTATGGCCATGTACACGAGATTCTTGAT | ATCAAGAATCTCGTGTACATGGCCATAACAGTCAAGAATTTCCCGG |
| **A075L_N208A** | ATACAACGATTCCCAGAGTGCATGAGTGTCAATGCCATTTCC | GGAAATGGCATTGACACTCATGCACTCTGGGAATCGTTGTAT |
| **A075L_D73A** | GTTCTGATATGTCGTAGGCCGAGTGACCAGTCACC | GGTGACTGGTCACTCGGCCTACGACATATCAGAAC |

**Supplementary Table 3.** Concentrations of the acceptors and donor in the enzymatic reactions monitored by HPLC.

|  | **A075L (μM)** | **Αcceptor Substrate**  **(mM)** | **UDP-Xyl (1)**  **(mM)** | **Mn^2+^**  **(mM)** | **Mg^2+^**  **(mM)** | **EDTA**  **(mM)** | **Reaction volume (μL)** |
| --- | --- | --- | --- | --- | --- | --- | --- |
| **a.** | 38.3 | **2**: 0.750 | 1.25 | 2 | 2 | – | 150 |
| **b.** | 38.3 | **4**: 0.750 | 1.25 | 2 | 2 | – | 150 |
| **c.*** | 25.6 | **6**: 0.375 | 0.625 | 2 | 2 | – | 150 |
| **d.*** | 25.6 | **6**: 0.375 | 0.625 | – | – | 0.5 | 150 |

*The donor and of the acceptor concentrations were purposely decreased because otherwise the reaction was too fast to be followed. Regarding to reaction **d**, A075L was first incubated with EDTA (ethylenediaminetetraacetic acid; Sigma-Aldrich product number E9884) in PBS for 30 min at 25 °C and then other reagents were added.

**Acceptor and Product synthesis**

General Synthetic Methods

All reagents were purchased from commercial sources and were used without further purification unless noted. Reaction solvents were purified by successive passage through columns of alumina and copper under argon. Unless stated otherwise, all reactions were carried out at room temperature and under a positive pressure of argon and were monitored by TLC on Silica Gel G-25 F254 (0.25 mm). Visualization of the reaction components was achieved using UV fluorescence (254 nm) and/or by charring with acidified anisaldehyde solution in ethanol, acetic acid and sulfuric acid. Organic solvents were evaporated under reduced pressure, and the products were purified by column chromatography on silica gel (230−400 mesh) or size exclusion column chromatography (Sephadex-LH20). Optical rotations were measured in a microcell (1 cm, 1 mL) at ambient temperature and are in units of degrees·mL/(g·dm). ^1^H NMR spectra were recorded at 400 MHz, 500 MHz, 600 MHz or 700 MHz and chemical shifts are referenced to residual CHCl_3_ (7.26 ppm, CDCl_3_), CHD_2_OD (3.30 ppm, CD_3_OD), HDO (4.78 ppm, D_2_O). ^13^C NMR spectra were recorded at 125 MHz or 175 MHz and chemical shifts are referenced to CDCl_3_ (77.0 ppm) or CD_3_OD (49.3 ppm). Reported splitting patterns are abbreviated as s = singlet, d = doublet, t = triplet, m = multiplet, br = broad, app = apparent. Assignments of NMR spectra were based on two-dimensional experiments (^1^H−^1^H COSY, HSQC and HMBC. High-resolution ESI-MS spectra (time-of-flight analyzer) were recorded on samples suspended in THF or CH_3_OH and with added NaCl. Compounds **3** and **8** were prepared as previously reported^26^.

***Synthesis of 4***

**Octyl 4-*O*-benzyl-α-D-rhamnopyranosyl-(1→3)-4-*O*-acetyl-α-L-fucopyranoside (S2):** To a stirred solution of **S1**^1^ (148 mg, 249 μmol) in CH_3_CN–CH_3_OH (5.5 mL, 10:1) was added *p*-toluenesulfonic acid monohydrate (142 mg, 748 μmol) at room temperature. The reaction mixture was stirred for 3.5 h at room temperature. Excess triethylamine was added to quench the acid, the mixture was filtered, and the filtrate was concentrated. The crude residue was purified by flash chromatography (1:2 hexane–EtOAc) to afford **S2** (110 mg, 79%) as a syrup. *R*_f_ 0.29 (1:2 hexane–EtOAc); [α]_D_ –58.3 (*c* 0.50, CHCl_3_); ^1^H NMR (600 MHz; CDCl_3_): *δ* 7.39–7.36 (m, 4H, Ar), 7.34–7.31 (m, 1H, Ar), 5.20 (dd, 1H, *J* = 3.3, 0.8 Hz, Fuc-H-4), 5.17 (d, 1H, *J* = 1.5 Hz, Rha-H-1), 4.87 (d, 1H, *J* = 3.9 Hz, Fuc-H-1), 4.78 (d, 1H, *J* = 11.6 Hz, PhCH_2_), 4.68 (d, 1H, *J* = 11.6 Hz, PhCH_2_), 4.07–4.03 (m, 2H, Fuc-H-5, Rha-H-2), 3.94 (dd, 1H, *J* = 9.9, 3.4 Hz, Fuc-H-3), 3.90–3.84 (m, 2H, Fuc-H-2, Rha-H-5), 3.81 (dd, 1H, *J* = 9.2, 3.4 Hz, Rha-H-3), 3.72 (dt, 1H, *J* = 9.7, 6.8 Hz, OCH_2_CH_2_(CH_2_)_5_CH_3_), 3.47 (dt, 1H, *J* = 9.7, 6.7 Hz, OCH_2_CH_2_(CH_2_)_5_CH_3_), 3.37 (t, 1H, *J* = 9.3 Hz, Rha-H-4), 2.15 (s, 3H, COCH_3_), 1.66–1.61 (m, 2H, OCH_2_CH_2_(CH_2_)_5_CH_3_), 1.37–1.27 (m, 13H, Rha-H-6, OCH_2_CH_2_(CH_2_)_5_CH_3_), 1.14 (d, 3H, *J* = 6.6 Hz, Fuc-H-6), 0.91 (t, 3H, *J* = 7.0 Hz, OCH_2_CH_2_(CH_2_)_5_CH_3_); ^13^C NMR (125 MHz CDCl_3_): *δ* 170.5 (C=O), 138.4 (Ar), 128.6 (Ar), 128.0 (2 × Ar), 101.0 (Rha-C-1), 98.6 (Fuc-C-1), 81.2 (Rha-C-4), 74.8 (Fuc-C-3), 74.2 (PhCH_2_), 73.0 (Fuc-C-4), 71.0 (Rha-C-2), 70.6 (Rha-C-3), 69.5 (Fuc-C-2), 68.6 (OCH_2_(CH_2_)_6_CH_3_), 67.8 (Rha-C-5), 65.3 (Fuc-C-5), 31.8 (OCH_2_(CH_2_)_6_CH_3_), 29.5 (OCH_2_(CH_2_)_6_CH_3_), 29.4 (OCH_2_(CH_2_)_6_CH_3_), 29.2 (OCH_2_(CH_2_)_6_CH_3_), 26.2 (OCH_2_(CH_2_)_6_CH_3_), 22.7 (OCH_2_(CH_2_)_6_CH_3_), 20.8 (COCH_3_), 18.0 (Rha-C-6), 16.1 (Fuc-C-6), 14.1 (OCH_2_(CH_2_)_6_CH_3_); HRMS (ESI) Calc. for [M + NH_4_]^+^ C_29_H_50­_­NO_10_: 572.3429; Found 572.3431.

**Octyl α-D-rhamnopyranosyl-(1→3)-α-L-fucopyranoside (4):** To a stirred solution of **S2** (104 mg, 188 μmol) in CH_3_OH (4.0 mL) was added a solution of NaOCH_3_ in CH_3_OH (0.4 mL, 0.5 M). The reaction mixture was stirred overnight at room temperature, then neutralized by addition of Amberlite® IR-120 (H^+^) cation exchange resin, filtered and the filtrate was concentrated. The residue was then dissolved in THF–CH_3_OH (1:1, 3.0 mL) and 20% palladium hydroxide on carbon (10.0 mg) was added. After stirring for 24 h under an H_2_ atmosphere (1 atm), the reaction mixture was filtered through Celite and concentrated. The residue was dissolved in water and then lyophilized to afford **4** (79.3 mg, quant.) as a white solid. *R*_f_ 0.45 (5:1 CH_2_Cl_2_–CH_3_OH); [α]_D_ –16.7 (*c* 0.40, CH_3_OH); ^1^H NMR (600 MHz; CD_3_OD): *δ* 5.01 (d, 1H, *J* = 1.5 Hz, Rha-H-1), 4.72 (d, 1H, *J* = 3.8 Hz, Fuc-H-1), 3.97–3.93 (m, 2H, Rha-H-2, Fuc-H-5), 3.86 (dd, 1H, *J* = 10.2, 3.9 Hz, Fuc-H-2), 3.80–3.73 (m, 3H, Fuc-H-3, Rha-H-3, Rha-H-5), 3.71 (d, 1H, *J* = 2.8 Hz, Fuc-H-4), 3.65 (dt, 1H, *J* = 9.7, 7.0 Hz, OCH_2_CH_2_(CH_2_)_5_CH_3_), 3.45 (dt, 1H, *J* = 9.7, 6.4 Hz, OCH_2_CH_2_(CH_2_)_5_CH_3_), 3.38 (app t, 1H, *J* = 9.5 Hz, Rha-H-4), 1.66–1.59 (m, 2H, OCH_2_CH_2_(CH_2_)_5_CH_3_), 1.35–1.28 (m, 10H, OCH_2_CH_2_(CH_2_)_5_CH_3_), 1.24 (d, 3H, *J* = 6.3 Hz, Rha-H-6), 1.19 (d, 3H, *J* = 6.6 Hz, Fuc-H-6), 0.89 (app t, 3H, *J* = 7.0 Hz, OCH_2_CH_2_(CH_2_)_5_CH_3_); ^13^C NMR (125 MHz CD_3_OD): *δ* 103.8 (Rha-C-1), 100.5 (Fuc-C-1), 78.9 (Fuc-C-3), 74.1 (Rha-C-4), 73.5 (Fuc-C-4), 72.2 (Rha-C-2), 72.1 (Rha-C-3), 70.1 (Rha-C-5), 69.4 (2 × C, Fuc-C-2, OCH_2_(CH_2_)_6_CH_3_), 67.6 (Fuc-C-5), 33.0 (OCH_2_(CH_2_)_6_CH_3_), 30.6 (OCH_2_(CH_2_)_6_CH_3_), 30.5 (OCH_2_(CH_2_)_6_CH_3_), 30.4 (OCH_2_(CH_2_)_6_CH_3_), 27.4 (OCH_2_(CH_2_)_6_CH_3_), 23.7 (OCH_2_(CH_2_)_6_CH_3_), 18.0 (Rha-C-5), 16.6 (Fuc-C-5), 14.4 (OCH_2_(CH_2_)_6_CH_3_); HRMS (ESI) Calc. for [M + Na]^+^ C_20_H_38­_­NaO_9_: 445.2408; Found 445.2406.

***Synthesis of 5***

**Octyl 2,3-di-*O*-benzyl-4,6-*O*-di-*tert*-butylsilylene-α-D-galactopyranosyl-(1→2)-3,4-*O*-isopropylidene-α-L-fucopyranoside (S5):** To a stirred solution of acceptor **S3**^1^ (152 mg, 480 μmol) and 2,3-di-*O*-benzyl-4,6-*O*-di-*tert*-butylsilylene-α-D-galactopyranoside **S4**^11^ (565 mg, 932 μmol) in dry CH_2_Cl_2_ (5.0 mL) was added molecular sieves (500 mg, 4Å, powder). After stirring for 30 min at room temperature, the reaction mixture was cooled to –10 °C, and then *N*-iodosuccinimide (252 mg, 1.12 mmol) and silver trifluoromethanesulfonate (23.1 mg, 89.0 μmol) were added successively. The resulting solution was stirred for 1h at 0°C under an Ar atmosphere. Excess triethylamine was added to quench the acid, the mixture was filtered, and the filtrate was washed with saturated Na_2_S_2_O_3_ (aq.) and saturated NaHCO_3_ (aq.), the aqueous layer was extracted with CH_2_Cl_2_ (50 mL × 3), dried over Na_2_SO_4_, filtered and the filtrate was concentrated. The crude residue was purified by flash chromatography (8:1 hexane–EtOAc) to afford **S5** (316 mg, 82%) as a syrup. *R*_f_ 0.24 (8:1 hexane–EtOAc); [α]_D_ +17.6 (*c* 0.26, CHCl_3_); ^1^H NMR (600 MHz; CDCl_3_): *δ* 7.46–7.44 (m, 4H, Ar), 7.36–7.30 (m, 6H, Ar), 5.10 (d, 1H, *J* = 3.8 Hz, Gal-H-1), 4.84–4.82 (m, 2H, PhCH_2_, Fuc-H-1), 4.76 (d, 1H, *J* = 12.6 Hz, PhCH_2_), 4.75 (s, 2H, PhCH_2_) 4.55 (d, 1H, *J* = 2.7 Hz, Gal-H-4), 4.36 (dd, 1H, *J* = 7.8, 5.6 Hz, Fuc-H-3), 4.22 (dd, 1H, *J* = 12.3, 2.1 Hz, Gal-H-6a), 4.10–4.05 (m, 3H, Fuc-H-5, Gal-H-6b, Fuc-H-4), 4.01 (dd, 1H, *J* = 10.1, 3.8 Hz, Gal-H-2), 3.88 (dd, 1H, *J* = 10.1, 3.0 Hz, Gal-H-3), 3.82 (br s, 1H, Gal-H-5), 3.71 (dd, 1H, *J* = 7.8, 3.5 Hz, Fuc-H-2), 3.65 (dt, 1H, *J* = 9.6, 6.8 Hz, OCH_2_CH_2_(CH_2_)_5_CH_3_), 3.25 (dt, 1H, *J* = 9.6, 6.7 Hz, OCH_2_CH_2_(CH_2_)_5_CH_3_), 1.56 (s, 3H, C(CH_3_)_2_), 1.55–1.51 (m, 2H, OCH_2_CH_2_(CH_2_)_5_CH_3_), 1.39 (s, 3H, C(CH_3_)_2_), 1.35 (d, 3H, *J* = 6.6 Hz, Fuc-H-6), 1.32–1.27 (m, 10H, OCH_2_CH_2_(CH_2_)_5_CH_3_), 1.08 (s, 9H, C(CH_3_)_3_), 1.00 (s, 9H, C(CH_3_)_3_), 0.90 (app t, 3H, *J* = 7.0 Hz, OCH_2_CH_2_(CH_2_)_5_CH_3_); ^13^C NMR (125 MHz CDCl_3_): *δ* 139.2 (Ar), 138.9 (Ar), 128.3 (Ar), 128.1 (2 × Ar), 127.5 (Ar), 127.4 (Ar), 127.3 (Ar), 108.8 (C(CH_3_)_2_), 99.9 (Gal-H-1, ^1^*J*_C-H_ = 170.6 Hz), 98.1 (Fuc-C-1), 78.4 (Fuc-C-2), 77.5 (Gal-C-3), 76.3 (Fuc-C-4), 74.6 (Fuc-C-3), 74.3 (Gal-C-2), 72.5 (PhCH_2_), 71.2 (Gal-C-4), 71.1 (PhCH_2_), 68.1 (OCH_2_(CH_2_)_6_CH_3_), 67.5 (Gal-C-5), 67.4 (Gal-C-6), 63.3 (Fuc-C-5), 31.9 (OCH_2_(CH_2_)_6_CH_3_), 29.6 (OCH_2_(CH_2_)_6_CH_3_), 29.4 (OCH_2_(CH_2_)_6_CH_3_), 29.3 (OCH_2_(CH_2_)_6_CH_3_), 28.4 (C(CH_3_)_2_), 27.6 (C(CH_3_)_3_), 27.3 (C(CH_3_)_3_), 26.4 (C(CH_3_)_2_), 26.2 (OCH_2_(CH_2_)_6_CH_3_), 23.4 (C(CH_3_)_3_), 22.7 (OCH_2_(CH_2_)_6_CH_3_), 20.7 (C(CH_3_)_3_), 16.4 (Fuc-C-6), 14.1 (OCH_2_(CH_2_)_6_CH_3_); HRMS (ESI) Calc. for [M + Na]^+^ C_45_H_70­_­NaO_10_Si: 821.4630; Found 821.4632.

**Octyl 2,3-di-*O*-benzyl-α-D-galactopyranosyl-(1→2)-α-L-fucopyranoside (S6):** To a stirred solution of **S5** (316 mg 395 μmol) in THF–pyridine (6.0 mL, 1:1) was added HF∙pyridine (0.6 mL, pyridine ∼30%, hydrogen fluoride ∼70%) at 0 °C under an Ar atmosphere. The reaction mixture was stirred for 1 h at 0 °C, before being poured into saturated NaHCO_3_ (aq.). The aqueous layer was extracted with EtOAc (40 mL × 3), dried over Na_2_SO_4_, filtered and the filtrate was concentrated. The crude residue was then dissolved in CH_3_CN–CH_3_OH (5.5 mL, 10:1) and *p*-toluenesulfonic acid monohydrate (223 mg, 1.17 mmol) was added at room temperature. The reaction mixture was stirred for 3.5 h at room temperature. Excess triethylamine was added to quench the acid, the mixture was filtered and the filtrate was concentrated. The crude residue was purified by flash chromatography (1:2 hexane–EtOAc) to afford **S6** (183 mg, 75%) as a syrup. *R*_f_ 0.15 (2:3 hexane–EtOAc); [α]_D_ –1.5 (*c* 0.45, CHCl_3_); ^1^H NMR (600 MHz; CDCl_3_): *δ* 7.41–7.31 (m, 10H, Ar), 4.95 (d, 1H, *J* = 3.7 Hz, Gal-H-1), 4.93 (d, 1H, *J* = 3.7 Hz, Fuc-H-1), 4.87 (d, 1H, *J* = 11.6 Hz, PhCH_2_), 4.79 (d, 1H, *J* = 11.4 Hz, PhCH_2_), 4.73 (d, 1H, *J* = 11.4 Hz, PhCH_2_), 4.69 (d, 1H, *J* = 11.6 Hz, PhCH_2_), 4.15 (dd, 1H, *J* = 3.2, 1.0 Hz, Gal-H-4), 4.04–3.99 (m, 3H, Fuc-H-3, Gal-H-5, Gal-H-3), 3.97–3.92 (m, 2H, Fuc-H-5, Gal-H-6a), 3.90 (app t, 1H, *J* = 4.9 Hz, Gal-H-2), 3.85 (d, 1H, *J* = 2.4 Hz, Fuc-H-4), 3.78 (dd, 1H, *J* = 11.6, 4.0 Hz, Gal-H-6b), 3.74 (dd, 1H, *J* = 9.9, 3.7 Hz, Fuc-H-2), 3.68 (dt, 1H, *J* = 9.7, 6.8 Hz, OCH_2_CH_2_(CH_2_)_5_CH_3_), 3.42 (dt, 1H, *J* = 9.7, 6.7 Hz, OCH_2_CH_2_(CH_2_)_5_CH_3_), 1.61–1.56 (m, 2H, OCH_2_CH_2_(CH_2_)_5_CH_3_), 1.35–1.28 (m, 10H, OCH_2_CH_2_(CH_2_)_5_CH_3_), 0.91 (app t, 3H, *J* = 7.0 Hz, OCH_2_CH_2_(CH_2_)_5_CH_3_); ^13^C NMR (175 MHz CDCl_3_): *δ* 137.8 (Ar), 137.6 (Ar), 128.6 (2 × Ar), 128.2 (Ar), 128.1 (Ar), 128.0 (Ar), 127.7 (Ar), 101.5 (Gal-C-1), 98.0 (Fuc-C-1), 80.5 (Fuc-C-2), 78.0 (Gal-C-3), 75.9 (Gal-C-2), 74.3 (PhCH_2_), 72.4 (PhCH_2_), 71.3 (Fuc-C-4), 69.7 (Fuc-C-3), 69.1 (Gal-C-5), 68.8 (Gal-C-4), 68.2 (OCH_2_(CH_2_)_6_CH_3_), 65.3 (Fuc-C-5), 63.2 (Gal-C-6), 31.8 (OCH_2_(CH_2_)_6_CH_3_), 29.6 (OCH_2_(CH_2_)_6_CH_3_), 29.4 (OCH_2_(CH_2_)_6_CH_3_), 29.3 (OCH_2_(CH_2_)_6_CH_3_), 26.3 (OCH_2_(CH_2_)_6_CH_3_), 22.6 (OCH_2_(CH_2_)_6_CH_3_), 16.1 (Fuc-C-6), 14.1 (OCH_2_(CH_2_)_6_CH_3_); HRMS (ESI) Calc. for [M + Na]^+^ C_34_H_50­_­NaO_10_: 641.3296; Found 641.3297.

**Octyl α-D-galactopyranosyl-(1→2)-α-L-fucopyranoside (5):** To a stirred solution of **S6** (129 mg, 208 μmol) in THF–CH_3_OH (1:1, 4.0 mL) and 20% palladium hydroxide on carbon (15.0 mg) was added. After stirring for 24 h under an H_2_ atmosphere (1 atm), the reaction mixture was filtered through Celite and concentrated. The residue was dissolved in water and then lyophilized to afford **5** (91.3 mg, quant.) as a white solid. *R*_f_ 0.29 (4:1 CH_2_Cl_2_–CH_3_OH); [α]_D_ –5.4 (*c* 0.52, CH_3_OH); ^1^H NMR (600 MHz; CD_3_OD): *δ* 5.00 (d, 1H, *J* = 3.7 Hz, Fuc-H-1), 4.99 (d, 1H, *J* = 3.0 Hz, Gal-H-1), 3.99–3.97 (m, 1H), 3.95–3.91 (m, 2H), 3.88 (app t, 1H, *J* = 1.3 Hz), 3.76–3.75 (m, 2H), 3.74–3.69 (m, 3H), 3.68–3.63 (m, 2H), 3.48 (dt, 1H, *J* = 9.6, 6.5 Hz), 1.59 (quintet, 2H, *J* = 7.0 Hz), 1.40–1.28 (m, 10H), 1.20 (d, 3H, *J* = 6.6 Hz), 0.90 (app t, 3H, *J* = 7.1 Hz); ^13^C NMR (125 MHz CD_3_OD): *δ* 103.7 (Gal-C-1), 99.8 (Fuc-C-1), 80.0, 73.6, 72.8, 71.6, 71.2, 70.8, 70.4, 68.6, 67.2, 62.7, 33.1, 30.8, 30.6, 30.5, 27.6, 23.7, 16.6, 14.4; HRMS (ESI) Calc. for [M + Na]^+^ C_20_H_38­_NaO_10_: 461.2357; Found 461.2356.

***Synthesis of 6***

**Octyl 4-*O*-benzyl-α-D-rhamnopyranosyl-(1→3)-[2,3-di-*O*-benzyl-*O*-α-D-galactopyranosyl-(1→2)]-4-*O*-acetyl-α-L-fucopyranoside (S8):** To a stirred solution of **S7**^1^ (145 mg 135 μmol) in THF–pyridine (3.0 mL, 1:1) was added HF∙pyridine (0.3 mL, pyridine ∼30%, hydrogen fluoride ∼70%) at 0 °C under an Ar atmosphere. The reaction mixture was stirred for 1h at 0 °C, before being poured into saturated NaHCO_3_ (aq.). The aqueous layer was extracted with EtOAc (25 mL × 3), dried over Na_2_SO_4_, filtered and the filtrate was concentrated. The crude residue was then dissolved in CH_3_CN–CH_3_OH (3.3 mL, 10:1) and *p*-toluenesulfonic acid monohydrate (74.1 mg, 390 μmol) was added at room temperature. The reaction mixture was stirred for 3.5 h at room temperature. Excess triethylamine was added to quench the acid, the mixture was filtered, and the filtrate was concentrated. The crude residue was purified by flash chromatography (1:2 hexane–EtOAc) to afford **S8** (97.9 mg, 81%) as a syrup. *R*_f_ 0.30 (2:3 hexane–EtOAc); [α]_D_ +3.9 (*c* 0.50, CHCl_3_); ^1^H NMR (600 MHz; CDCl_3_): *δ* 7.40–7.29 (m, 15H, Ar), 5.26 (d, 1H, *J* = 3.0 Hz, Fuc-H-4), 5.13 (d, 1H, *J* = 1.9 Hz, Rha-H-1), 5.02 (d, 1H, *J* = 3.4 Hz, Fuc-H-1), 4.94 (d, 1H, *J* = 3.2 Hz, Gal-H-1), 4.82 (dd, 1H, *J* = 11.9 Hz, PhCH_2_), 4.81 (dd, 1H, *J* = 11.4 Hz, PhCH_2_), 4.74–4.66 (m, 4H, PhCH_2_), 4.22 (dd, 1H, *J* = 10.2, 3.5 Hz, Fuc-H-3), 4.15 (br s, 1H, Gal-H-4), 4.06–4.02 (m, 2H, Gal-H-5, Fuc-H-5), 3.95–3.91 (m, 2H, Gal-H-3, Gal-H-6a), 3.87 (dd, 1H, *J* = 10.0, 3.1 Hz, Gal-H-2), 3.84–3.77 (m, 4H, Fuc-H-2, Rha-H-5, Rha-H-2, Gal-H-6b), 3.72 (d, 1H, *J* = 7.4 Hz, Rha-H-3), 3.64 (dt, 1H, *J* = 9.4, 6.8 Hz, OCH_2_CH_2_(CH_2_)_5_CH_3_), 3.37 (dt, 1H, *J* = 9.4, 6.7 Hz, OCH_2_CH_2_(CH_2_)_5_CH_3_), 3.26 (app t, 1H, *J* = 8.7 Hz, Rha-H-4), 2.21 (s, 3H, COCH_3_), 1.62–1.57 (m, 2H, OCH_2_CH_2_(CH_2_)_5_CH_3_), 1.35–1.28 (m, 13H, Rha-H-6, OCH_2_CH_2_(CH_2_)_5_CH_3_), 1.12 (d, 3H, *J* = 6.5 Hz, Fuc-H-6), 0.91 (app t, 3H, *J* = 7.0 Hz, OCH_2_CH_2_(CH_2_)_5_CH_3_); ^13^C NMR (125 MHz CDCl_3_): *δ* 170.4 (C=O), 138.6 (Ar), 138.1 (Ar), 138.0 (Ar), 128.6 (2 × Ar), 128.4 (Ar), 128.3 (Ar), 128.0 (Ar), 127.9 (Ar), 127.8 (Ar), 127.7 (Ar), 101.7 (Rha-C-1), 100.9 (Gal-C-1), 98.1 (Fuc-C-1), 81.6 (Rha-C-4), 80.0 (Fuc-C-2), 77.4 (Gal-C-3), 76.0 (Gal-C-2), 74.0 (PhCH_2_), 73.6 (PhCH_2_), 73.5 (Fuc-C-4), 72.9 (PhCH_2_), 71.6 (Fuc-C-3), 70.7 (Rha-C-3), 70.6 (Rha-C-2), 69.6 (Gal-C-4), 69.3 (Gal-C-5), 68.0 (OCH_2_(CH_2_)_6_CH_3_), 67.5 (Rha-C-5), 64.5 (Fuc-C-5), 63.4 (Gal-C-6), 31.9 (OCH_2_(CH_2_)_6_CH_3_), 29.8 (OCH_2_(CH_2_)_6_CH_3_), 29.5 (OCH_2_(CH_2_)_6_CH_3_), 29.3 (OCH_2_(CH_2_)_6_CH_3_), 26.5 (OCH_2_(CH_2_)_6_CH_3_), 22.7 (OCH_2_(CH_2_)_6_CH_3_), 20.9 (COCH_3_), 18.3 (Rha-C-6), 16.0 (Fuc-C-6), 14.1 (OCH_2_(CH_2_)_6_CH_3_); HRMS (ESI) Calc. for [M + Na]^+^ C_49_H_68­_­NaO_15_: 919.4450; Found 919.4451.

**Octyl α-D-rhamnopyranosyl-(1→3)-[α-D-galactopyranosyl-(1→2)]-α-L-fucopyranoside (6):** To a stirred solution of **S8** (94.6 mg, 106 μmol) in CH_3_OH (3.0 mL) was added a solution of NaOCH_3_ in CH_3_OH (0.3 mL, 0.5 M). The reaction mixture was stirred overnight at room temperature, then neutralized by addition of Amberlite® IR-120 (H^+^) cation exchange resin, filtered and the filtrate was concentrated. The residue was then dissolved in THF–CH_3_OH (1:1, 3.0 mL) and 20% palladium hydroxide on carbon (10.0 mg) was added. After stirring for 4 h under an H_2_ atmosphere (1 atm), the reaction mixture was filtered through Celite and concentrated. The residue was dissolved in water and then lyophilized to afford **6** (61.6 mg, quant.) as a white solid. *R*_f_ 0.25 (3:1 CH_2_Cl_2_–CH_3_OH); [α]_D_ +21.6 (*c* 0.52, CH_3_OH); ^1^H NMR (600 MHz; CD_3_OD): *δ* 5.07 (d, 1H, *J* = 1.6 Hz, Rha-H-1), 4.98–4.97 (m, 2H, Gal-H-1, Fuc-H-1), 4.00 (dd, 1H, *J* = 3.4, 1.7 Hz), 3.97–3.89 (m, 4H), 3.87 (dd, 1H, *J* = 2.9, 1.1 Hz), 3.77–3.64 (m, 8H), 3.48 (dt, 1H, *J* = 9.7, 6.5 Hz), 3.38 (app t, 1H, *J* = 9.5 Hz), 1.63–1.58 (m, 2H), 1.34–1.30 (m, 10H), 1.24 (d, 3H, *J* = 6.2 Hz), 1.19 (d, 3H, *J* = 6.6 Hz), 0.90 (app t, 3H, *J* = 7.0 Hz); ^13^C NMR (125 MHz CD_3_OD): *δ* 104.0 (Rha-C-1), 103.0 (Gal-C-1), 99.7 (Fuc-C-1), 78.1, 76.9, 74.1, 73.7, 72.8, 72.2, 71.9, 71.4, 71.2, 70.4, 70.2, 68.8, 67.2, 62.8, 33.1, 30.8, 30.6, 30.5, 27.6, 23.7, 18.0, 16.6, 14.5; HRMS (ESI) Calc. for [M + Na]^+^ C_26_H_48_NaO_14_: 607.2936; Found 607.2934.

***Synthesis of 7***

**Octyl 2,3,6-tri-*O*-acetyl-3-*O*-allyl-β-D-glucopyranoside (S10):** To a stirred solution of **S9**^12^ 5.272 g, 13.57 mmol) and *n*-octanol (6.2 mL, 40 mmol) in dry CH_2_Cl_2_ (35.0 mL) was added boron trifluoride ethyl etherate (4.2 mL, 34 mmol) drop-wise at 0 ˚C, under an Ar atmosphere. The reaction mixture was stirred at 0 ˚C for 1 h then warmed to room temperature and stirred for 16 h and then poured into saturated NaHCO_3_ (aq). The aqueous layer was extracted with CH_2_Cl_2_ (60 mL × 3) and the combined organic layers washed with brine, dried over Na_2_SO_4_, filtered and the filtrate was then concentrated. The resulting residue was dissolved in pyridine (50.0 mL) and acetic anhydride (42.0 mL) was added at 0 ˚C. The reaction mixture was warmed to room temperature and stirred for 20 h and then concentrated. The residue was diluted with CH_2_Cl_2_ (150 mL) and washed with 10% HCl (aq), saturated NaHCO_3_ (aq), and brine. The organic layer was dried over Na_2_SO_4_, filtered and the filtrate was then concentrated. The crude residue was purified via flash chromatography (2.5:1 hexanes–EtOAc) to give **S10** (3.086 g, 50%) as a colourless syrup. *R*_f_ 0.57 (2:1 hexanes–EtOAc); [α]ᴅ –26.5 (*c* 1.1, CHCl_3_); ^1^H NMR (500 MHz; CDCl_3_): δ 5.79 (ddt, 1H, *J* = 17.2, 10.4, 5.6 Hz, OCH_2_CH=CH_2_), 5.22 (app dq, 1H, *J* = 17.2, 1.6 Hz, OCH_2_CH=CH_2_), 5.15 (app dq, 1H, *J* = 10.4, 1.4 Hz, OCH_2_CH=CH_2_), 5.08 (app t, 1H, *J* = 9.6 Hz, H-4), 5.00 (dd, 1H, *J* = 9.6, 7.9 Hz, H-2), 4.41 (d, 1H, *J* = 7.9 Hz, H-1), 4.23 (dd, 1H, *J* = 12.2, 5.1 Hz. H-6a), 4.14 (dd, 1H, *J* = 12.2, 2.7 Hz, H-6b), 4.12–4.04 (m, 2H, OCH_2_CH=CH_2_), 3.87 (dt, 1H, *J* = 9.6, 6.3 Hz, OCH_2_(CH_2_)_6_CH_3_), 3.61 (app t, 1H, *J* = 9.4 Hz, H-3), 3.63–3.56 (m, 1H, H-5), 3.46 (dt, 1H, *J* = 9.6, 6.8 Hz, OCH_2_(CH_2_)_6_CH_3_), 2.11 (s, 3H, COCH_3_), 2.10–2.07 (m, 6H, COCH_3_), 1.63–1.49 (m, 2H, OCH_2_CH_2_(CH_2_)_5_CH_3_), 1.36–1.26 (m, 10H, OCH_2_CH_2_(CH_2_)_5_CH_3_), 0.90 (t, 3H, *J* = 6.9 Hz, O(CH_2_)_7_CH_3_); ^13^C NMR (126 MHz; CDCl_3_): δ 170.8 (COCH_3_), 169.3 (COCH_3_), 169.1 (COCH_3_), 134.3 (OCH_2_CH=CH_2_), 116.9 (OCH_2_CH=CH_2_), 101.1 (C-1), 79.8 (C-3), 72.6 (OCH_2_CH=CH_2_), 72.5 (C-2), 72.0 (C-5), 70.0 (OCH_2_(CH_2_)_6_CH_3_), 69.7 (C-4), 62.4 (C-6), 31.8 (OCH_2_(CH_2_)_6_CH_3_), 29.4 (OCH_2_(CH_2_)_6_CH_3_), 29.3 (OCH_2_(CH_2_)_6_CH_3_), 29.2(5) (OCH_2_(CH_2_)_6_CH_3_), 25.8 (OCH_2_(CH_2_)_6_CH_3_), 22.6 (OCH_2_(CH_2_)_6_CH_3_), 20.9 (COCH_3_), 20.8(5) (COCH_3_), 20.8 (COCH_3_), 14.1 (O(CH_2_)_7_CH_3_); HRMS (ESI) Calc. for [M + Na]^+^ C_23_H_38_NaO_9_: 481.2408; Found 481.2408.

**Octyl 3-*O*-allyl-β-D-glucopyranoside (S11):** To a stirred solution of **S10** (3.017 g, 6.579 mmol) in CH_3_OH (20.0 mL) was added NaOCH_3_ in CH_3_OH (20.0 mL, 0.9 M). The reaction mixture was stirred at room temperature for 17 h. Amberlite® IR-120 (H^+^) cation exchange resin was added, the mixture filtered and then the filtrate was concentrated. The crude residue was purified via flash chromatography (18:1 CH_2_Cl_2_–CH_3_OH) to give **S11** (1.915 g, 88%) as a white solid. *R*_f_ 0.67 (9:1 CH_2_Cl_2_–CH_3_OH); [α]ᴅ –21.5 (*c* 2.8, CH_3_OH); ^1^H NMR (500 MHz; CD_3_OD): δ 5.99 (ddt, 1H, *J* = 17.3, 10.4, 5.8 Hz, OCH_2_CH=CH_2_), 5.26 (app dq, 1H, *J* = 17.3, 1.7 Hz, OCH_­2_CH=CH_2_), 5.10 (app dt, 1H, *J* = 10.4, 1.5 Hz, OCH_2_CH=CH_2_), 4.40–4.28 (m, 2H, OCH_2_CH=CH_2_), 4.23 (d, 1H, *J* = 7.4 Hz, H-1), 3.92‒3.80 (m, 2H, OCH_2_(CH_2_)_6_CH_3_, H-6a), 3.65 (dd, 1H, *J* = 11.9, 5.7 Hz, H-6b), 3.52 (dt, 1H, *J* = 9.5, 6.8 Hz, OCH_2_(CH_2_)_6_CH_3_), 3.39–3.32 (m, 1H, H-4), 3.28–3.18 (m, 3H, H-2, H-5, H-3), 1.65–1.55 (m, 2H, OCH_2_CH_2_(CH_2_)_5_CH_3_), 1.42–1.23 (m, 10H, OCH_2_CH_2_(CH_2_)_5_CH_3_), 0.89 (t, 3H, *J* = 6.9 Hz, O(CH_2_)_7_CH_3_); ^13^C NMR (126 MHz; CD_3_OD): δ 137.1 (OCH_2_CH=CH_2_), 116.7 (OCH_2_CH=CH_2_), 104.4 (C-1), 86.0 (C-3), 77.8, 75.2 (C-5 & C-2), 75.1 (OCH_2_CH=CH_2_), 71.4 (C-4), 71.0 (OCH_2_(CH_2_)_6_CH_3_), 62.8 (C-6), 33.0 (OCH_2_(CH_2_)_6_CH_3_), 30.8 (OCH_2_(CH_2_)_6_CH_3_), 30.6 (OCH_2_(CH_2_)_6_CH_3_), 30.4 (OCH_2_(CH_2_)_6_CH_3_), 27.1 (OCH_2_(CH_2_)_6_CH_3_), 23.7 (OCH_2_(CH_2_)_6_CH_3_), 14.4 (O(CH_2_)_7_CH_3_); HRMS (ESI) Calc. for [M + Na]^+^ C_17_H_32_NaO_6_: 355.2091; Found 355.2091.

**Octyl 3-*O*-allyl-4,6-*O*-benzylidene-β-D-glucopyranoside (S12):** To a stirred solution of **S11** (1.890 g, 5.686 mmol) in dry CH_3_CN (25.0 mL) was added benzaldehyde dimethyl acetal (2.56 mL, 17.1 mmol) and camphorsulfonic acid (397.7 mg, 1.712 mmol) at room temperature, under an Ar atmosphere. The reaction mixture was stirred at room temperature for 23 h before triethylamine was added and the solvent evaporated. The crude residue was purified via flash chromatography (6:1 hexanes–EtOAc) to give **S12** (2.066 g, 86%) as a white solid. *R*_f_ 0.55 (4:1 hexanes–EtOAc); [α]ᴅ –38.6 (*c* 0.3, CHCl_3_); ^1^H NMR (500 MHz; CDCl_3_): δ 7.54–7.48 (m, 2H, Ar), 7.44–7.35 (m, 3H, Ar), 5.99 (ddt, 1H, *J* = 17.3, 10.4, 5.8 Hz, OCH_­2_­CH=CH_2_), 5.57 (s, 1H, ArCH), 5.33 (app dq, 1H, *J* = 17.2, 1.7 Hz, OCH_2_CH=CH_2_), 5.21 (app dq, 1H, *J* = 10.3, 1.4 Hz, OCH_2_CH=CH_2_), 4.46 (app ddt, 1H, *J* = 12.8, 5.6, 1.5 Hz, OCH_2_CH=CH_2_), 4.42 (d, 1H, *J* = 7.6 Hz, H-1), 4.37 (dd, 1H, *J* = 10.5, 5.0 Hz, H-6a), 4.31 (app ddt, 1H, *J* = 12.8, 6.0, 1.4 Hz, OCH_2_CH=CH_2_), 3.91 (dt, 1H, *J* = 9.5, 6.9 Hz, OCH_2_(CH_2_)_6_CH_3_), 3.82 (app t, 1H, *J* = 10.3 Hz, H-6b), 3.69–3.52 (m, 4H, H-4, H-3, OCH_2_(CH_2_)_6_CH_3_, H-2), 3.46 (ddd, 1H, *J* = 10.1, 9.0, 5.0 Hz, H-5), 2.51 (d, 1H, *J* = 2.3 Hz, 2-OH), 1.73–1.61 (m, 2H, OCH_2_CH_2_(CH_2_)_5_CH_3_), 1.41–1.25 (m, 10H, OCH_2_CH_2_(CH_2_)_5_CH_3_), 0.91 (t, 3H, *J* = 6.8 Hz, O(CH_2_)_7_CH_3_); ^13^C NMR (126 MHz; CDCl_3_): δ 137.3 (Ar), 134.4 (OCH_2_CH=CH_2_), 129.0 (Ar), 128.3 (Ar), 126.0 (Ar), 117.3 (OCH_2_CH=CH_2_), 103.3 (C-1), 101.3 (ArCH), 81.5 (C-4), 80.0 (C-3), 74.2 (C-2), 73.6 (OCH_2_CH=CH_2_), 70.6 (OCH_2_(CH_2_)_6_CH_3_), 68.8 (C-6), 66.5 (C-5), 31.8 (OCH_2_(CH_2_)_6_CH_3_), 29.6 (OCH_2_(CH_2_)_6_CH_3_), 29.4 (OCH_2_(CH_2_)_6_CH_3_), 29.2 (OCH_2_(CH_2_)_6_CH_3_), 25.9 (OCH_2_(CH_2_)_6_CH_3_), 22.7 (OCH_2_(CH_2_)_6_CH_3_), 14.1 (O(CH_2_)_7_CH_3_); HRMS (ESI) Calc. for [M + Na]^+^ C_24_H_36_NaO_6_: 443.2404; Found 443.2401.

**Octyl 3-*O*-allyl-2-*O*-benzyl-4,6-*O*-benzylidene-β-D-glucopyranoside (S13):** To a stirred solution of **S12** (2.032 g, 4.831 mmol) in dry DMF was added sodium hydride (389.1 mg, 9.727 mmol, 60% dispersion in mineral oil) in one portion at 0 ˚C, under an Ar atmosphere. The mixture was stirred at 0 ˚C for 30 min and then benzyl bromide (1.2 mL, 10 mmol) was added drop-wise. The reaction mixture was slowly warmed to room temperature and stirred for 19 h. The mixture was chilled to 0 ˚C and CH_3_OH was added. The solution was diluted with CH_2_Cl_2_ (100 mL) and washed with saturated NaHCO_3_ (aq) and water. The aqueous layers were extracted with CH_2_Cl_2_ (70 mL × 3) and the combined organic layers were dried over Na_2_SO_4_, filtered and then the filtrate was concentrated. The crude residue was purified via flash chromatography (12:1 hexanes–EtOAc) to give **S13** (2.244 g, 91%) as a white solid. *R*_f_ 0.58 (5:1 hexanes–EtOAc); [α]ᴅ –31.1 (*c* 2.5, CHCl_3_); ^1^H NMR (500 MHz; CDCl_3_): δ 7.55–7.49 (m, 2H, Ar), 7.45–7.29 (m, 8H, Ar), 5.98 (ddt, 1H, *J* = 17.3, 10.4, 5.7 Hz, OCH_­2_­CH=CH_2_), 5.57 (s, 1H, ArCH), 5.32 (app dq, 1H, *J* = 17.2, 1.7 Hz, OCH_­2_­CH=CH_2_), 5.19 (app dq, 1H, *J* = 10.4, 1.4 Hz, OCH_­2_­CH=CH_2_), 4.92 (d, 1H, *J* = 10.9 Hz, ArCH_2_), 4.80 (d, 1H, *J* = 10.9 Hz, ArCH_2_), 4.52 (d, 1H, *J* = 7.7 Hz, H-1), 4.42 (app ddt, 1H, *J* = 12.6, 5.7, 1.5 Hz, OCH_2_CH=CH­_2_), 4.37 (dd, 1H, *J* = 10.5, 5.0 Hz, H-6a), 4.30 (app ddt, 1H, *J* = 12.6, 5.8, 1.5 Hz, OCH_2_CH=CH­_2_), 3.95 (dt, 1H, *J* = 9.4, 6.5 Hz, OCH_2_(CH_2_)_6_CH_3_), 3.81 (app t, 1H, *J* = 10.3 Hz, H-6b), 3.70–3.62 (m, 2H, H-3, H-4), 3.59 (dt, 1H, *J* = 9.5, 6.9 Hz, OCH_2_(CH_2_)_6_CH_3_), 3.48–3.37 (m, 2H, H-2, H-5), 1.75–1.61 (m, 2H, OCH_2_CH_2_(CH_2_)_5_CH_3_), 1.50–1.25 (m, 10H, OCH_2_CH_2_(CH_2_)_5_CH_3_), 0.91 (t, 3H, *J* = 6.9 Hz, O(CH_2_)_7_CH_3_); ^13^C NMR (126 MHz; CDCl_3_): δ 138.5 (Ar), 137.4 (Ar), 135.2 (OCH_2_CH=CH_2_), 128.9 (Ar), 128.3 (Ar), 128.3 (Ar), 128.2 (Ar), 127.7 (Ar), 126.0 (Ar), 116.8 (OCH_2_CH=CH_2_), 104.2 (C-1), 101.1 (ArCH), 82.1 (C-2), 81.5, 80.7 (C-3 & C-4), 75.4 (ArCH_2_), 74.1 (OCH_2_CH=CH_2_), 70.7 (OCH_2_(CH_2_)_6_CH_3_), 68.9 (C-6), 66.1 (C-5), 31.9 (OCH_2_(CH_2_)_6_CH_3_), 29.8 (OCH_2_(CH_2_)_6_CH_3_), 29.4 (OCH_2_(CH_2_)_6_CH_3_), 29.3 (OCH_2_(CH_2_)_6_CH_3_), 26.2 (OCH_2_(CH_2_)_6_CH_3_), 22.7 (OCH_2_(CH_2_)_6_CH_3_), 14.1 (O(CH_2_)_7_CH_3_); HRMS (ESI) Calc. for [M + Na]^+^ C_31_H_42_NaO_6_: 533.2874; Found 533.2873.

**Octyl 3-*O*-allyl-2,6-*O*-benzyl-β-D-glucopyranoside (S14):** To a stirred solution of **2.29** (1.312 g, 2.569 mmol) in dry CH_2_Cl_2_ (10.0 mL) was added triethylsilane (1.6 mL, 20 mmol) and trifluoroacetic acid (2.4 mL, 15 mmol) successively at 0 ˚C, under an Ar atmosphere. The reaction mixture was stirred at 0 ˚C for 3 h and then poured into saturated NaHCO_3_ (aq). The aqueous layer was extracted with CH_2_Cl_2_ (70 mL × 3) and the combined organic layers dried over Na_2_SO_4_, filtered and then the filtrate was concentrated. The crude residue was purified via flash chromatography (4:1 hexanes–EtOAc) to give **S14** (1.095 g, 83%) as a colourless syrup. *R*_f_ 0.26 (4:1 hexanes–EtOAc); [α]ᴅ –23.2 (*c* 2.4, CHCl_3_); ^1^H NMR (500 MHz; CDCl_3_): δ 7.40–7.30 (m, 10H, Ar), 5.97 (ddt, 1H, *J* = 17.2, 10.3, 5.8 Hz, OCH_2_CH=CH_2_), 5.30 (app dq, 1H, *J* = 17.2, 1.5 Hz, OCH_2_CH=CH_2_), 5.20 (app dq, 1H, *J* = 10.4, 1.3 Hz, OCH_2_CH=CH_2_), 4.95 (d, 1H, *J* = 11.0 Hz, ArCH_2_), 4.72 (d, 1H, *J* = 11.0 Hz, ArCH_2_), 4.64, 4.61 (ABq, 2H, *J*_AB_ = 12.1 Hz, ArCH_2_), 4.45–4.40 (m, 2H, OCH_2_CH=CH_2_, H-1), 4.27 (app ddt, 1H, *J* = 12.6, 6.1, 1.3 Hz, OCH_2_CH=CH_2_), 3.96 (dt, 1H, *J* = 9.4, 6.5 Hz, OCH_2_(CH_2_)_6_CH_3_), 3.82 (dd, 1H, *J* = 10.4, 4.0 Hz, H-6a), 3.75 (dd, 1H, *J* = 10.4, 5.4 Hz, H-6b), 3.61–3.56 (m, 1H, H-4), 3.54 (dt, 1H, *J* = 9.4, 6.7 Hz, OCH_2_(CH_2_)_6_CH_3_), 3.48 (ddd, 1H, *J* = 9.5, 5.4, 4.1 Hz, H-5), 3.39–3.35 (m, 2H, H-3, H-2), 2.73 (br s, 1H, 4-OH), 1.70–1.64 (m, 2H, OCH_2_CH_2_(CH_2_)_5_CH_3_), 1.45–1.29 (m, 10H, OCH_2_CH_2_(CH_2_)_5_CH_3_), 0.90 (t, 3H, *J* = 7.0 Hz, O(CH_2_)_7_CH_3_); ^13^C NMR (126 MHz; CDCl_3_): δ 138.5 (Ar), 138.0 (Ar), 135.2 (OCH_2_CH=CH_2_), 128.4 (Ar), 128.3 (Ar), 128.1 (Ar), 127.7(4) (Ar), 127.7(2) (Ar), 127.6 (Ar), 117.1 (OCH_2_CH=CH_2_), 103.7 (C-1), 83.8, 81.7 (C-2 & C-3), 74.7 (ArCH_2_), 74.2 (OCH_2_CH=CH_2_), 73.9 (C-5), 73.7 (ArCH_2_), 71.8 (C-4), 70.5 (C-6), 70.2 (OCH_2_(CH_2_)_6_CH_3_), 31.9 (OCH_2_(CH_2_)_6_CH_3_), 29.8 (OCH_2_(CH_2_)_6_CH_3_), 29.4 (OCH_2_(CH_2_)_6_CH_3_), 29.3 (OCH_2_(CH_2_)_6_CH_3_), 26.2 (OCH_2_(CH_2_)_6_CH_3_), 22.7 (OCH_2_(CH_2_)_6_CH_3_), 14.1 (O(CH_2_)_7_CH_3_); HRMS (ESI) Calc. for [M + Na]^+^ C_31_H_44_NaO_6_: 535.3030; Found 535.3030.

**2,3,4-tri-*O*-acetyl-1-thio-β-D-xylopyranose 1-(*N*-phenyl)-2,2,2-trifluoroacetimidate (S15):** To a stirred solution of **S16**^13^ (2.159 g, 5.645 mmol) in acetone–H_2_O (9:1, 45.0 mL acetone, 5.0 mL H_2_O) was added *N*-bromosuccinimide (4.013 g, 22.55 mmol). The reaction mixture was stirred at room temperature for 1.5 h and then poured to a saturated solution of Na_2_S_2_O_3_ (40 mL). The aqueous layer was extracted with EtOAc (80 mL × 3) and the combined organic layers were washed with saturated NaHCO_3_ (aq), water, and brine; dried over Na_2_SO_4_, filtered and then the filtrate was concentrated. The crude residue was passed through a silica column (2:1→1:1 hexanes–EtOAc). The resulting residue was dissolved dry CH_2_Cl_2_ (30.0 mL) and 2,2,2-trifluoro-*N*-phenylacetimidoyl chloride (1.80 mL, 11.4 mmol) and cesium carbonate (5.010 g, 15.38 mmol) were added under an Ar atmosphere. The reaction mixture was stirred at room temperature for 16 h and then filtered and the filtrate was concentrated. The crude residue was purified via flash chromatography (3:1 hexanes–EtOAc) to give **S15** (1.815 g, 72%) as an off white solid. The product was confirmed by HRMS (ESI). *R*_f_ 0.32 (3:1 hexanes–EtOAc). HRMS (ESI) Calc. for [M + Na]^+^ C_19_H_20_F_3_NNaO_8_: 470.1033; Found 470.1028.

**Octyl β-D-xylopyranosyl-(1→4)-3-*O*-allyl-2,6-di-*O*-benzyl-β-D-glucopyranoside (S17):** To a stirred solution of acceptor **S14** (1.106 g, 2.158 mmol) and donor **S15** (1.355 g, 3.028 mmol) in dry CH_2_Cl_2_ (24.0 mL) was added oven-dried molecular sieves (2.4 g, 4Å, powder) under an Ar atmosphere. After stirring at room temperature for 30 min, the solution was chilled to –30 ˚C and trifluoromethanesulfonic acid (0.04 mL, 0.5 mmol) was added drop-wise. The resulting solution was slowly warmed to –25 ˚C and stirred for a total of 25 min before triethylamine was added. The solution was filtered through Celite and the filtrate was concentrated. The crude residue was passed through a silica column (2:1 hexanes–EtOAc). The resulting product was dissolved in CH_3_OH (30.0 mL) and NaOCH_3_ in CH_3_OH (5.0 mL, 1.0 M) was added. The reaction mixture was stirred at room temperature for 13 h. Amberlite® IR-120 (H^+^) cation exchange resin was added, the mixture filtered and then the filtrate was concentrated. The crude residue was purified via flash chromatography (12:1 CH_2_Cl_2_–CH_3_OH) to give **S17** (1.117 g, 80%) as a white solid. *R*_f_ 0.58 (9:1 CH_2_Cl_2_–CH_3_OH); [α]ᴅ +12.1 (*c* 1.9, CHCl_3_); ^1^H NMR (500 MHz; CDCl_3_): δ 7.41 – 7.27 (m, 10H, Ar), 5.96 (ddt, 1H, *J* = 17.3, 10.3, 5.7 Hz, OCH_2_CH=CH_2_), 5.26 (app dq, 1H, *J* = 17.2, 1.7 Hz, OCH_2_CH=CH_2_), 5.16 (app dq, 1H, *J* = 10.4, 1.4 Hz, OCH_2_CH=CH_2_), 4.91 (d, 1H, *J* = 10.9 Hz, ArCH_2_), 4.74‒4.68 (m, 2H, ArCH_2_), 4.61 (d, 1H, *J* = 12.1 Hz, ArCH_2_), 4.55 (d, 1H, *J* = 7.4 Hz, Xyl-H-1), 4.40 – 4.29 (m, 2H, OCH_2_CH=CH_2_), 4.35 (d, 1H, *J* = 7.8 Hz, Glc-H-1), 4.07 (s, 1H, OH), 3.99 – 3.90 (m, 3H, Xyl-H-5a, Glc-H-6a, OCH_2_(CH_2_)_6_CH_3_), 3.87 (app t, 1H, *J* = 9.3 Hz, Glc-H-4), 3.77 (dd, 1H, *J* = 11.7, 2.5 Hz, Glc-H-6b), 3.65 (ddd, 1H, *J* = 10.0, 8.6, 5.3 Hz, Xyl-H-4), 3.52 (dt, 1H, *J* = 9.5, 6.8 Hz, OCH_2_(CH_2_)_6_CH_3_), 3.48 (app t, 1H, *J* = 9.0 Hz, Glc-H-3), 3.45 – 3.40 (m, 2H, Xyl-H-3, Glc-H-5), 3.38 (dd, 1H, *J* = 9.1, 7.8 Hz, Glc-H-2), 3.27 (dd, 1’

H, *J* = 9.0, 7.4 Hz, Xyl-H-2), 3.21 (dd, 1H, *J* = 11.6, 10.0 Hz, Xyl-H-5b), 2.87 (s, 1H, OH), 1.73 – 1.59 (m, 2H, OCH_2_CH_2_(CH_2_)_5_CH_3_), 1.47 – 1.26 (m, 10H, OCH_2_CH_2_(CH_2_)_5_CH_3_), 0.90 (t, 3H, *J* = 6.9 Hz, O(CH_2_)_7_CH_3_); ^13^C NMR (126 MHz; CDCl_3_): δ 138.4 (Ar), 137.4 (Ar), 135.2 (OCH_2_CH=CH_2_), 128.5 (Ar), 128.4 (Ar), 128.2 (Ar), 128.0 (Ar), 127.9 (Ar), 127.7 (Ar), 116.7 (OCH_2_CH=CH_2_), 103.9 (Xyl-C-1), 103.7 (Glc-C-1), 83.1 (Glc-C-3), 82.2 (Glc-C-2), 77.6 (Glc-C-4), 76.4 (Xyl-C-3), 75.0 (ArCH_2_), 74.5 (OCH_2_CH=CH_2_), 74.3, 74.1 (Xyl-C-2 & Glc-C-5), 73.8 (ArCH_2_), 70.2 (OCH_2_(CH_2_)_6_CH_3_), 69.6 (Xyl-C-4), 68.9 (Glc-C-6), 65.8 (Xyl-C-5), 31.9 (OCH_2_(CH_2_)_6_CH_3_), 29.8 (OCH_2_(CH_2_)_6_CH_3_), 29.4 (OCH_2_(CH_2_)_6_CH_3_), 29.3 (OCH_2_(CH_2_)_6_CH_3_), 26.2 (OCH_2_(CH_2_)_6_CH_3_), 22.7 (OCH_2_(CH_2_)_6_CH_3_),, 14.1 (O(CH_2_)_7_CH_3_); HRMS (ESI) Calc. for [M + Na]^+^ C_36_H_52_NaO_10_: 667.3453; Found 667.3451.

**Octyl 2,3,4-*O*-benzyl-β-D-xylopyranosyl-(1→4)-3-*O*-allyl-2,6-di-*O*-benzyl-β-D-glucopyranoside (S18):** To a stirred solution of **S17** (1.117 g, 1.732 mmol) in dry DMF was added sodium hydride (0.250 g, 6.25 mmol, 60% dispersion in mineral oil) in one portion at 0 ˚C, under an Ar atmosphere. The mixture was stirred at 0 ˚C for 1 h then benzyl bromide (0.72 mL, 8.9 mmol) was added drop-wise. The reaction mixture was slowly warmed to room temperature and stirred for 19.5 h. The mixture was chilled to 0 ˚C, CH_3_OH was added and then poured into a saturated NaHCO_3_ (aq). The aqueous layer was extracted with Et_2_O (120 mL × 3) and the combined organic layers were washed with brine, dried over Na_2_SO_4_, filtered and then the filtrate was concentrated. The crude residue was purified via flash chromatography (6:1 hexanes–EtOAc) to give **S18** (1.104 g, 70%) as a colourless syrup. *R*_f_ 0.53 (4:1 hexanes–EtOAc); [α]ᴅ +11.0 (*c* 1.7, CHCl_3_); ^1^H NMR (700 MHz; CDCl_3_): δ 7.43–7.26 (m, 25H, Ar), 5.99 (ddt, 1H, *J* = 17.4, 10.4, 6.0 Hz, OCH_2_CH=CH_2_), 5.25 (app dq, 1H, *J* = 17.3, 1.7 Hz, OCH_2_CH=CH_2_), 5.15 (app dt, 1H, *J* = 10.3, 1.6 Hz, OCH_2_CH=CH_2_), 4.91 (d, 1H, *J* = 11.0 Hz, ArCH_2_), 4.91, 4.87 (ABq, 2H, *J*_AB_ = 11.0 Hz, ArCH_2_), 4.81, 4.79 (ABq, 2H, *J*_AB_ = 11.2 Hz, ArCH_2_),4.77‒4.72 (m, 2H, ArCH_2_), 4.65 (d, 1H, *J* = 11.7 Hz, ArCH_2_), 4.58 (d, 1H, *J* = 12.1 Hz, ArCH_2_), 4.44 (d, 1H, *J* = 12.1 Hz, ArCH_2_), 4.43 (d, 1H, *J* = 7.8 Hz, Xyl-H-1), 4.39 (app ddt, 1H, *J* = 11.8, 6.0, 1.4 Hz, OCH_2_CH=CH_2_), 4.36 (d, 1H, *J* = 7.7 Hz, Glc-H-1), 4.28 (app ddt, 1H, *J* = 11.9, 6.0, 1.4 Hz, OCH_2_CH=CH_2_), 3.99–3.92 (m, 2H, OCH_2_(CH_2_)_6_CH_3_, Xyl-H-5a), 3.87 (dd, 1H, *J* = 9.8, 8.8 Hz, Glc-H-4), 3.80 (dd, 1H, *J* = 10.9, 4.4 Hz, Glc-H-6a), 3.73 (dd, 1H, *J* = 10.9, 1.9 Hz, Glc-H-6b), 3.61 (ddd, 1H, *J* = 10.2, 8.8, 5.3 Hz, Xyl-H-4), 3.56–3.50 (m, 2H, OCH_2_(CH_2_)_6_CH_3_, Xyl-H-3), 3.43 (app t, 1H, *J* = 9.0 Hz, Glc-H-3), 3.40–3.35 (m, 2H, Glc-H-2, Glc-H-5), 3.31 (dd, 1H, *J* = 9.2, 7.7 Hz, Xyl-H-2), 3.10 (dd, 1H, *J* = 11.8, 10.2 Hz, Xyl-H-5b), 1.72–1.62 (m, 2H, OCH_2_CH_2_(CH_2_)_5_CH_3_), 1.48–1.27 (m, 10H, OCH_2_CH_2_(CH_2_)_5_CH_3_), 0.92 (t, 3H, *J* = 7.0 Hz, O(CH_2_)_7_CH_3_); ^13^C NMR (176 MHz; CDCl_3_): δ 138.8 (Ar), 138.7 (Ar), 138.5 (Ar), 138.3(4) (Ar), 138.3 (Ar), 135.7 (OCH_2_CH=CH_2_), 128.5 (Ar), 128.4 (Ar), 128.3(3) (Ar), 128.3 (Ar), 128.2(8) (Ar), 128.2 (Ar), 127.9 (Ar), 127.8 (Ar), 127.7(9) (Ar), 127.7(6) (Ar), 127.7 (Ar), 127.6 (Ar), 127.5(9) (Ar), 127.5(7) (Ar), 127.5 (Ar), 116.7 (OCH_2_CH=CH_2_), 103.6 (Glc-C-1), 103.2 (Xyl-C-1), 84.1 (Xyl-C-3), 82.5 (Glc-C-3), 82.4 (Xyl-C-2), 81.7 (Glc-C-2), 78.3 (Xyl-C-4), 76.9 (Glc-C-4), 75.6 (ArCH_2_), 75.1(3) (Glc-C-5), 75.1 (ArCH_2_), 75.0(8) (ArCH_2_), 74.6 (OCH_2_CH=CH_2_), 73.3 (ArCH_2_), 73.2 (ArCH_2_), 70.1 (OCH­_2_(CH_2_)_6_CH_3_), 68.3 (Glc-C-6), 63.9 (Xyl-C-5), 31.9 (OCH_2_(CH_2_)_6_CH_3_), 29.8 (OCH_2_(CH_2_)_6_CH_3_), 29.5 (OCH_2_(CH_2_)_6_CH_3_), 29.3 (OCH_2_(CH_2_)_6_CH_3_), 26.2 (OCH_2_(CH_2_)_6_CH_3_), 22.7 (OCH_2_(CH_2_)_6_CH_3_), 14.2 (O(CH_2_)_7_CH_3_); HRMS (ESI) Calc. for [M + Na]^+^ C_57_H_70_NaO_10_: 937.4861; Found 937.4864.

**Octyl 2,3,4-*O*-benzyl-β-ᴅ-xylopyranosyl-(1→4)-2,6-di-*O*-benzyl-β-D-glucopyranoside (S19):** A solution of **S18** (1.104 g, 1.206 mmol) in dry THF (10.0 mL) was degassed via vacuum and (1,5-Cyclooctadiene)bis(methyldiphenylphosphine) iridium(I) hexafluorophosphate (61.8 mg, 73.1 µmol) was added under an Ar atmosphere. The resulting mixture was stirred at 0 ˚C for 20 min before the catalyst was activated with hydrogen (stirring for 2 min under a hydrogen atmosphere). Excess hydrogen was removed by three cycles of vacuum purging with Ar. The reaction mixture was stirred at room temperature for 3 h under an Ar atmosphere and then concentrated. The residue was dissolved in acetone**–**water (10:1, 11.0 mL) and HgO (0.368 g, 1.70 mmol) and HgCl_2_ (0.398 g, 1.47 mmol) were added. The reaction mixture was stirred at room temperature for 2.5 h and then concentrated. The residue was diluted with EtOAc (100 mL) and was washed with 10% KI (aq), saturated Na_2_S_2_O_3_ (aq), and water. The aqueous layers were extracted with EtOAc (100 mL) and the combined organic layers were dried over Na_2_SO_4_, filtered and then the filtrate was concentrated. The crude residue was purified via flash chromatography (5:1 hexanes**–**EtOAc) to give **S19** (0.960 g, 91%) as a syrup. *R*_f_ 0.57 (3:1 hexanes**–**EtOAc); [α]ᴅ +12.5 (*c* 0.7, CHCl_3_); ^1^H NMR (700 MHz; CDCl_3_): δ 7.44**–**7.40 (m, 2H, Ar), 7.38**–**7.23 (m, 23H, Ar), 4.91 (d, 1H, *J* = 11.4 Hz, ArCH_2_), 4.90, 4.87 (ABq, 2H, *J*_AB_ = 11.0 Hz, ArCH_2_), 4.82 (d, 1H, *J* = 11.4 Hz, ArCH_2_), 4.79, 4.78 (ABq, 2H, *J*_AB_ = 11.1 Hz, ArCH_2_), 4.73 (d, 1H, *J* = 11.7 Hz, ArCH_2_), 4.61 (d, 1H, *J* = 11.7 Hz, ArCH_2_), 4.44 (d, 1H, *J* = 12.1 Hz, ArCH_2_), 4.39 (d, 1H, *J* = 7.8 Hz, Xyl-H-1), 4.33 (d, 1H, *J* = 12.1 Hz, ArCH_2_), 4.26 (d, 1H, *J* = 7.8 Hz, Glc-H-1), 3.96**–**3.89 (m, 3H, OCH_2_(CH_2_)_6_CH_3_, Xyl-H-5a, Glc-3-OH), 3.71‒3.64 (m, 3H, Glc-H-6a, Xyl-H-3, Glc-H-6b), 3.61 (ddd, 1H, *J* = 10.4, 8.9, 5.4 Hz, Xyl-H-4), 3.57 (dd, 1H, *J* = 9.8, 8.7 Hz, Glc-H-4), 3.54**–**3.49 (m, 2H, Glc-H-3, OCH_2_(CH_2_)_6_CH_3_), 3.46 (ddd, 1H, *J* = 9.8, 4.6, 1.8 Hz, Glc-H-5), 3.35‒3.31 (m, 2H, Glc-H-2, Xyl-H-2), 3.16 (dd, 1H, *J* = 11.7, 10.4 Hz, Xyl-H-5b), 1.70**–**1.60 (m, 2H, OCH_2_CH_2_(CH_2_)_5_CH_3_), 1.45**–**1.23 (m, 10H, OCH_2_CH_2_(CH_2_)_5_CH_3_), 0.89 (t, 3H, *J* = 7.0 Hz, O(CH_2_)_7_CH_3_); ^13^C NMR (176 MHz; CDCl_3_): δ 138.8 (Ar), 138.5 (Ar), 138.3 (Ar), 138.2 (Ar), 138.0 (Ar), 128.5 (Ar), 128.4 (Ar), 128.3(5) (Ar), 128.3 (Ar), 128.2 (Ar), 128.0 (Ar), 127.9(3) (Ar), 127.9 (Ar), 127.8 (Ar), 127.7(4) (Ar), 127.7(3) (Ar), 127.7 (Ar), 127.6(6) (Ar), 127.5(3) (Ar), 127.5 (Ar), 103.8 (Glc-C-1), 103.1 (Xyl-C-1), 83.9 (Glc-C-3), 81.5, 81.4 (Glc-C-2 & Xyl-C-2), 80.2 (Glc-C-4), 77.7 (Xyl-C-4), 75.7 (ArCH_2_), 75.3 (ArCH_2_), 74.8 (Xyl-C-3), 74.6 (ArCH_2_), 74.3 (Glc-C-5), 73.5 (ArCH_2_), 73.2 (ArCH_2_), 70.2 (OCH_2_(CH_2_)_6_CH_3_), 68.3 (Glc-C-6), 64.0 (Xyl-C-5), 31.9 (OCH_2_(CH_2_)_6_CH_3_), 29.8 (OCH_2_(CH_2_)_6_CH_3_), 29.4 (OCH_2_(CH_2_)_6_CH_3_), 29.3 (OCH_2_(CH_2_)_6_CH_3_), 26.2 (OCH_2_(CH_2_)_6_CH_3_), 22.7 (OCH_2_(CH_2_)_6_CH_3_), 14.1 (O(CH_2_)_7_CH_3_); HRMS (ESI) Calc. for [M + Na]^+^ C_54_H_66_NaO_10_: 897.4548; Found 897.4552.

**Octyl 2,3,4-tri-*O*-benzyl-β-D-xylopyranosyl-(1→4)-[4-*O*-acetyl-3-*O*-allyl-2-*O*-(4-methoxybenzyl)-α-L-fucopyranosyl-(1→3)]-2,6-di-*O*-benzyl-β-D-glucopyranoside (S21):** To a stirred solution of acceptor **S19** (193.7 mg, 221.3 µmol) and donor **S20**^14^ (206.5 mg, 436.9 µmol) in dry Et_2_O (8.0 mL) was added oven-dried molecular sieves (0.8 g, 4Å, powder) under an Ar atmosphere. After stirring at room temperature for 45 min, methyl trifluoromethanesulfonate (0.10 mL, 914 µmol) was added drop-wise at room temperature. The reaction mixture was stirred at room temperature for 19 h before triethylamine was added. The solution was filtered through Celite and the filtrate was concentrated. The crude residue was purified via flash chromatography (5:1 hexanes–EtOAc) to give **S21** (238.5 mg, 88%) as a colourless syrup. *R*_f_ 0.63 (2.5:1 hexanes–EtOAc); [α]ᴅ –4.8 (*c* 2.1, CHCl_3_); ^1^H NMR (700 MHz; CDCl_3_): δ 7.36–7.20 (m, 25H, Ar), 7.08–7.04 (m, 2H, Ar), 6.67–6.62 (m, 2H, Ar), 5.89 (ddt, 1H, *J* = 17.2, 10.5, 5.3 Hz, OCH_2_CH=CH_2_), 5.63 (d, 1H, *J* = 3.7 Hz, Fuc-H-1), 5.28 (app dq, 1H, *J* = 17.3, 1.8 Hz, OCH_2_CH=CH_2_), 5.15‒5.10 (m, 2H, Fuc-H-4, OCH_2_CH=CH_2_), 4.99 (d, 1H, *J* = 11.6 Hz, ArCH_2_), 4.87 (app q, 1H, *J* = 6.7 Hz, Fuc-H-5), 4.84, 4.82 (ABq, 2H, *J*_AB_ = 11.0 Hz, ArCH_2_), 4.76 (d, 1H, *J* = 11.1 Hz, ArCH_2_), 4.73 (d, 1H, *J* = 11.7 Hz, ArCH_2_), 4.67 (d, 1H, *J* = 11.5 Hz, ArCH_2_), 4.67 (d, 1H, *J* = 11.2 Hz, ArCH_2_), 4.63 (d, 1H, *J* = 12.0 Hz, ArCH_2_), 4.60‒4.54 (m, 3H, ArCH_2_), 4.41 (d, 1H, *J* = 12.0 Hz, ArCH_2_), 4.39 (d, 1H, *J* = 8.0 Hz, Xyl-H-1), 4.35 (d, 1H, *J* = 7.8 Hz, Glc-H-1), 4.08 (app ddt, 1H, *J* = 10.8, 5.4, 1.9 Hz, OCH_2_CH=CH_2_), 4.05‒3.98 (m, 2H, Glc-H-4, Fuc-H-3), 3.96–3.85 (m, 4H, Glc-H6a, Glc-H-3, OCH_2_(CH_2_)_6_CH_3­_), 3.79 (dd, 1H, *J* = 11.7, 5.6 Hz, Xyl-H-5a), 3.72 (s, 3H, ArOCH_3_), 3.67 (dd, 1H, *J* = 10.1, 3.7 Hz, Fuc-H-2), 3.64 (dd, 1H, *J* = 11.1, 1.8 Hz, Glc-H-6b), 3.51 (dd, 1H, *J* = 9.1, 7.9 Hz, Glc-H-2), 3.46 (ddd, 1H, *J* = 10.6, 9.0, 5.5 Hz, Xyl-H-4), 3.45‒3.36 (m, 2H, OCH_2_(CH_2_)_6_CH_3_, Xyl-H-3), 3.32 (ddd, 1H, *J* = 9.8, 3.4, 1.8 Hz, Glc-H-5), 3.06 (app t, 1H, *J* = 8.6 Hz, Xyl-H-2), 2.95 (app t, 1H, *J* = 11.2 Hz, Xyl-H-5b), 2.11 (s, 3H, COCH_3_), 1.60–1.50 (m, 2H, OCH_2_CH_2_(CH_2_)_5_CH_3_), 1.33–1.14 (m, 10H, OCH_2_CH_2_(CH_2_)_5_CH_3_), 1.06 (d, 3H, *J* = 6.6 Hz, Fuc-H-6), 0.85 (t, 3H, *J* = 7.3 Hz, O(CH_2_)_7_CH_3_); ^13^C NMR (176 MHz; CDCl_3_): δ 170.8 (COCH_3_), 158.9 (Ar), 138.9 (Ar), 138.5 (Ar), 138.2 (Ar), 138.1(7) (Ar), 138.1(6) (Ar) 135.2 (OCH_2_CH=CH_2_), 130.6 (Ar), 129.6 (Ar), 128.5 (Ar), 128.4 (Ar), 128.3(4) (Ar), 128.3 (Ar), 128.2 (Ar), 128.0 (Ar), 127.9 (Ar), 127.8 (Ar), 127.6(4) (Ar), 127.6(3) (Ar), 127.6 (Ar), 127.1 (Ar), 126.7 (Ar), 116.1 (OCH_2_CH=CH_2_), 113.5 (Ar), 103.7 (Glc-C-1), 103.2 (Xyl-C-1), 97.3 (Fuc-C-1), 84.0 (Xyl-C-3), 83.0 (Glc-C-2), 82.5 (Xyl-C-2), 78.4 (Xyl-C-4), 76.2 (Fuc-C-3), 75.8 (ArCH_2_), 75.2 (Glc-C-5), 75.1(7) (ArCH_2_), 74.8, 74.6 (Glc-C-3 & Glc-C-4), 73.9 (Fuc-C-2), 73.6 (ArCH_2_), 73.5 (ArCH_2_), 73.3 (ArCH_2_), 72.8 (ArCH_2_), 71.6 (Fuc-C-4), 70.4 (OCH_2_CH=CH_2_), 70.2 (OCH_2_(CH_2_)_6_CH_3_), 67.7 (Glc-C-6), 64.1 (Xyl-C-5), 64.0 (Fuc-C-5), 55.2 (ArOCH_3_), 31.8 (OCH_2_(CH_2_)_6_CH_3_), 29.6 (OCH_2_(CH_2_)_6_CH_3_), 29.4 (OCH_2_(CH_2_)_6_CH_3_), 29.2 (OCH_2_(CH_2_)_6_CH_3_), 26.1 (OCH_2_(CH_2_)_6_CH_3_), 22.6 (OCH_2_(CH_2_)_6_CH_3_), 20.9 (COCH_3_), 16.1 (Fuc-C-6), 14.1 (O(CH_2_)_7_CH_3_); HRMS (ESI) Calc. for [M + Na]^+^ C_73_H_90_NaO_16_: 1245.6121; Found 1245.6120.

**Octyl 2,3,4-tri-*O*-benzyl-β-D-xylopyranosyl-(1→4)-[4-*O*-acetyl-2-*O*-(4-methoxybenzyl)-α-L-fucopyranosyl-(1→3)]-2,6-di-*O*-benzyl-β-D-glucopyranoside (S22):** A solution of **S21** (148.0 mg, 121.0 µmol) in dry THF (4.0 mL) was degassed via vacuum and (1,5-Cyclooctadiene)bis(methyldiphenylphosphine)iridium(I)Hexafluoro-phosphate (6.2 mg, 7.3 µmol) was added under an Ar atmosphere. The resulting mixture was stirred at 0 ˚C for 20 min before the catalyst was activated with hydrogen (stirring for 2 min under a hydrogen atmosphere). Excess hydrogen was removed by three cycles of vacuum purging with Ar. The reaction mixture was stirred at room temperature for 2.5 h under an Ar atmosphere and then concentrated. The residue was dissolved in acetone**–**water (10:1, 4.0 mL) and HgO (44.5 mg, 170 µmol) and HgCl_2_ (40.6 mg, 150 µmol) were added. The reaction mixture was stirred at room temperature for 2 h and then concentrated. The residue was diluted with EtOAc (80 mL) and was washed with 10% KI (aq), saturated Na_2_S_2_O_3_ (aq), and water. The aqueous layers were extracted with EtOAc (100 mL) and the combined organic layers were dried over Na_2_SO_4_, filtered and then the filtrate was concentrated. The crude residue was purified via flash chromatography (3:1 hexanes**–**EtOAc) to give **S22** (127.7 mg, 89%) as a syrup. *R*_f_ 0.30 (3:1 hexanes**–**EtOAc); [α]ᴅ **–**39.9 (*c* 2.0, CHCl_3_); ^1^H NMR (700 MHz; CDCl_3_): δ 7.37**–**7.22 (m, 25H, Ar), 7.04**–**7.00 (m, 2H, Ar), 6.79**–**6.74 (m, 2H, Ar), 5.74 (d, 1H, *J* = 3.5 Hz, Fuc-H-1), 5.16 (d, 1H, *J* = 11.9 Hz, ArCH_2_), 5.08 (dd, 1H, *J* = 3.6, 1.4 Hz, Fuc-H-4), 4.90 (app q, 1H, *J* = 6.3 Hz, Fuc-H-5), 4.83, 4.80 (ABq, 2H, *J*_AB_ = 11.0 Hz, ArCH_2_) , 4.76 (d, 1H, *J* = 11.1 Hz, ArCH_2_), 4.73 (d, 1H, *J* = 11.5 Hz, ArCH_2_), 4.67 (d, 1H, *J* = 11.1 Hz, ArCH_2_), 4.62 (d, 1H, *J* = 11.9 Hz, ArCH_2_), 4.60 (d, 1H, *J* = 11.9 Hz, ArCH_2_), 4.59 (d, 1H, *J* = 11.5 Hz, ArCH_2_), 4.55 (d, 1H, *J* = 11.8 Hz, ArCH_2_), 4.42‒4.26 (m, 3H, ArCH_2_, Glc-H-1, Xyl-H-1), 4.22 (app dt, 1H, *J* = 10.2, 3.0 Hz, Fuc-H-3), 4.13 (d, 1H, *J* = 11.8 Hz, ArCH_2_), 4.01 (app t, 1H, *J* = 9.5 Hz, Glc-H-4), 3.94 (app t, 1H, *J* = 9.2 Hz, Glc-H-3), 3.93‒3.87 (m, 2H, Glc-H-6a, OCH_2_(CH_2_)_6_CH_3_), 3.76 (s, 3H, ArOCH_3_), 3.78**–**3.72 (m, 1H, Xyl-H-5a), 3.64 (dd, 1H, *J* = 10.9, 1.9 Hz, Glc-H-6b), 3.55‒3.51 (m, 2H, Fuc-H-2, Glc-H-2), 3.49**–**3.42 (m, 2H, Xyl-H-4, OCH_2_(CH_2_)_6_CH_3_), 3.39‒3.33 (m, 2H, Xyl-H-3, Glc-H-5), 3.06 (app t, 1H, *J* = 8.6 Hz, Xyl-H-2), 2.93 (app t, 1H, *J* = 11.2 Hz, Xyl-H-5b), 2.08 (s, 3H, COCH_3_), 1.92 (d, 1H, *J* = 2.7 Hz, Fuc-3-OH), 1.59**–**1.51 (m, 2H, OCH_2_CH_2_(CH_2_)_5_CH_3_), 1.33**–**1.14 (m, 10H, OCH_2_CH_2_(CH_2_)_5_CH_3_), 1.08 (d, 3H, *J* = 6.6 Hz, Fuc-H-6), 0.84 (t, 3H, *J* = 7.2 Hz, O(CH_2_)_7_CH_3_); ^13^C NMR (176 MHz; CDCl_3_): δ 171.0 (COCH_3_), 159.3 (Ar), 138.9 (Ar), 138.5 (Ar), 138.3 (Ar), 138.2 (Ar), 138.1 (Ar), 129.9 (Ar), 129.5 (Ar), 128.5 (Ar), 128.4 (Ar), 128.3(3) (Ar), 128.3 (Ar), 128.2(9) (Ar), 128.0 (Ar), 127.9 (Ar), 127.8 (Ar), 127.7 (Ar), 127.6(2) (Ar), 127.6 (Ar), 127.1 (Ar), 126.4 (Ar), 113.8 (Ar), 103.6, 103.2 (Glc-C-1 & Xyl-C-1), 96.2 (Fuc-C-1), 83.9 (Xyl-C-3), 83.1 (Glc-C-2), 82.4 (Xyl-C-2), 78.5 (Xyl-C-4), 75.7 (ArCH_2_), 75.3, 75.2 (Fuc-C-2 & Glc-C-5), 75.1(7) (ArCH_2_), 74.9 (Glc-C-3, Glc-C-4), 73.6 (ArCH_2_), 73.5(9) (Fuc-C-4), 73.5(5) (ArCH_2_), 73.3 (ArCH_2_), 71.4 (ArCH_2_), 70.1 (OCH_2_(CH_2_)_6_CH_3_), 67.6 (Glc-C-6), 67.2 (Fuc-C-3), 64.2 (Fuc-C-5), 64.1 (Xyl-C-5), 55.2 (ArOCH_3_), 31.8 (OCH_2_(CH_2_)_6_CH_3_), 29.6 (OCH_2_(CH_2_)_6_CH_3_), 29.3 (OCH_2_(CH_2_)_6_CH_3_), 29.2 (OCH_2_(CH_2_)_6_CH_3_), 26.1 (OCH_2_(CH_2_)_6_CH_3_), 22.6 (OCH_2_(CH_2_)_6_CH_3_), 20.9 (COCH_3_), 16.1 (Fuc-C-6), 14.1 (O(CH_2_)_7_CH_3_); HRMS (ESI) Calc. for [M + Na]^+^ C_70_H_86_NaO_16_: 1205.5808; Found 1205.5808.

***p*-Tolyl 2,3,4-*O*-acetyl-1-thio-α-D-rhamnopyranoside (S23):** To a stirred solution of **S25** (750.7 mg, 2.777 mmol) in pyridine (6.0 mL) was added acetic anhydride (3.0 mL) at 0 ˚C. The reaction mixture was warmed to room temperature, stirred for 21 h and then concentrated. The residue was dissolved in EtOAc (20 mL) and washed with 1M HCl (aq), saturated NaHCO_3_ (aq), and water. The organic layer was dried over Na_2_SO_4_, filtered and then the filtrate wasconcentrated. The crude residue was purified via flash chromatography (3:1 hexanes**–**EtOAc) to give **S25** (956.1 mg, 87%) as a white solid. *R*_f_ 0.75 (1:1 hexanes**–**EtOAc); [α]ᴅ +104.7 (*c* 2.5, CHCl_3_); ^1^H NMR (500 MHz; CDCl_3_): δ 7.41**–**7.35 (m, 2H, Ar), 7.17**–**7.12 (m, 2H, Ar), 5.51 (dd, 1H, *J* = 3.4, 1.7 Hz, H-2), 5.35 (d, 1H, *J* = 1.6 Hz, H-1), 5.32 (dd, 1H, *J* = 10.0, 3.4 Hz, H-3), 5.16 (app t, 1H, *J* = 9.9 Hz, H-4), 4.39 (dq, 1H, *J* = 9.7, 6.2 Hz, H-5), 2.35 (s, 3H, ArCH_3_), 2.15 (s, 3H, COCH_3_), 2.10 (s, 3H, COCH_3_), 2.03 (s, 3H, COCH_3_), 1.26 (d, 3H, *J* = 6.2 Hz, H-6); ^13^C NMR (126 MHz; CDCl_3_): δ 170.0 (COCH_3_), 170.0 (COCH_3_), 169.9 (COCH_3_), 138.2 (Ar), 132.5 (Ar), 130.0 (Ar), 129.4 (Ar), 86.1 (C-1), 71.3, 71.2 (C-2 & C-4), 69.4 (C-3), 67.7 (C-5), 21.1 (ArCH_3_), 20.9 (COCH_3_), 20.8 (COCH_3_), 20.7 (COCH_3_), 17.4 (C-6); HRMS (ESI) Calc. for [M + Na]^+^ C_19_H_24_NaO_7_S: 419.1135; Found 419.1137.

***p*-Tolyl 1-thio-α-D-rhamnopyranoside (S25):** To a stirred solution of **S24**^15^ (201.6 mg, 0.7041 mmol) in dry pyridine (8.0 mL) was added a solution of 4-toluenesulfonyl chloride (162.7 mg, 0.8534 mmol) in dry pyridine (5.0 mL) drop-wise under an Ar atmosphere at 0 ˚C. The reaction mixture was warmed to room temperature and stirred for 7 h then the solvent evaporated. The residue was dissolved in chloroform (20.0 mL) and washed with brine, dried over Na_2_SO_4_, filtered and then the filtrate was concentrated. The resulting residue was dissolved in dry THF (7.0 mL) and was slowly added drop-wise at 0 ˚C to a solution of LiAlH_4_ (80.5 mg, 2.12 mmol) in dry THF (8.0 mL) under an Ar atmosphere. The reaction mixture was heated at reflux for 3.5 h and then chilled to 0 ˚C before slowly adding water, followed by 2N H_2_SO_4_ (10.0 mL). The aqueous layer was extracted with EtOAc (20 mL × 3) and the combined organic layers were dried over Na_2_SO_4_, filtered and then the filtrate was concentrated. The crude residue was purified via flash chromatography (3:1 EtOAc–hexanes) to give **S25** (105.6 mg, 56%) as a white solid. *R*_f_ 0.71 (6:1 CH_2_Cl_2_–CH_3_OH); ^1^H NMR (500 MHz; CDCl_3_): δ 7.36–7.31 (m, 2H, Ar), 7.11–7.05 (m, 2H, Ar), 5.43 (d, 1H, *J* = 1.4 Hz, H-1), 4.23 (dd, 1H, *J* = 3.3, 1.5 Hz, H-2), 4.19 (dq, 1H, *J* = 9.7, 6.7 Hz, H-5), 3.83 (dd, 1H, *J* = 9.6, 3.3 Hz, H-3), 3.58 (app t, 1H, *J* = 9.5 Hz, H-4), 2.32 (s, 3H, COCH_3_), 1.34 (d, 3H, *J* = 6.2 Hz, H-6); ^13^C NMR (126 MHz; CDCl_3_): δ 137.6 (Ar), 132.1 (Ar), 130.2 (Ar), 129.9 (Ar), 88.3 (C-1), 73.2 (C-4), 72.5 (C-2), 72.1 (C-3), 69.3 (C-5), 21.1 (ArCH_3_), 17.5 (C-6); HRMS (ESI) Calc. for [M + Na]^+^ C_13_H_18_NaO_4_S: 293.0818; Found 293.0816.

**Octyl 2,3,4-tri-*O*-acetyl-α-D-rhamnopyranosyl-(1→3)-4-*O*-acetyl-α-L-fucopyranosyl-(1→3)-[2,3,4-tri-*O*-benzyl-β-D-xylopyranosyl-(1→4)]-2,6-di-*O*-benzyl-β-D-glucopyranoside (S26):** To a stirred solution of acceptor **S22** (68.8 mg, 58.1 µmol) and donor **S23** (46.4 mg, 117 µmol) in dry CH_2_Cl_2_ (5.0 mL) was added oven-dried molecular sieves (0.5 g, 4Å, powder) under an Ar atmosphere. After stirring at room temperature for 50 min, *N*-iodosuccinimide (26.2 mg, 116 µmol) and trifluoromethanesulfonic acid (1.5µL, 17 µmol) were added successively at room temperature. The reaction mixture was stirred at room temperature for 50 min before triethylamine was added. The solution was diluted with CH_2_Cl_2_ (15 mL) and filtered through Celite. The filtrate was washed with saturated Na_2_S_2_O_3_ (aq) and saturated NaHCO_3_ (aq). The combined aqueous layers were extracted with CH_2_Cl_2_ (3 × 15 mL), dried over Na_2_SO_4_, filtered, and the filtrate was concentrated. The crude residue was passed through a silica column (2.5:1 hexanes–EtOAc). The resulting product was dissolved in dry CH_2_Cl_2_ (10.0 mL) and then trifluoroacetic acid (0.10 mL) was added drop-wise at 0 ˚C, under Ar atmosphere. The reaction mixture was slowly warmed to room temperature and stirred for 5 h and then was chilled to 0 ˚C and triethylamine added. The solution was concentrated and the resulting residue was purified via flash chromatography (3:1 hexanes–EtOAc) to give **S26** (49.5 mg, 64%) as a colourless syrup. *R*_f_ 0.57 (3:1 hexanes–EtOAc); [α]ᴅ –21.3 (*c* 1.0, CHCl_3_); ^1^H NMR (700 MHz; CDCl_3_): δ 7.36–7.22 (m, 25H, Ar), 5.53 (d, 1H, *J* = 4.0 Hz, Fuc-H-1), 5.35 (dd, 1H, *J* = 3.4, 1.8 Hz, Rha-H-2), 5.17 (dd, 1H, *J* = 10.2, 3.4 Hz, Rha-H-3), 5.13 (d, 1H, *J* = 1.8 Hz, Rha-H-1), 5.08 (dd, 1H, *J* = 3.6, 1.3 Hz, Fuc-H-4), 5.04 (app t, 1H, *J* = 10.0 Hz, Rha-H-4), 4.92 (d, 1H, *J* = 10.4 Hz, ArCH_2_), 4.80 (ABq, 2H, *J*_AB_ = 11.5 Hz, ArCH_2_), 4.78‒473 (m, 2H, Fuc-H-5, ArCH_2_), 4.71 (d, 1H, *J* = 11.2 Hz, ArCH_2_), 4.66 (d, 1H, *J* = 11.1 Hz, ArCH_2_), 4.63 (d, 1H, *J* = 12.0 Hz, ArCH_2_), 4.61 (d, 1H, *J* = 10.4 Hz, ArCH_2_), 4.58 (d, 1H, *J* = 11.2 Hz, ArCH_2_), 4.42 (d, 1H, *J* = 12.0 Hz, ArCH_2_), 4.38 (d, 1H, *J* = 8.0 Hz, Xyl-H-1), 4.35 (d, 1H, *J* = 7.7 Hz, Glc-H-1), 4.06 (dd, 1H, *J* = 9.9, 3.5 Hz, Fuc-H-3), 4.00 (dq, 1H, *J* = 9.8, 6.2 Hz, Rha-H-5), 3.94 (dd, 1H, *J* = 11.7, 5.6 Hz, Xyl-H-5a), 3.92–3.85 (m, 4H, OCH_2_(CH_2_)_6_CH_3_, Glc-H-6a, Fuc-H-2, Glc-H-4), 3.81 (app t, 1H, *J* = 9.3 Hz, Glc-H-3), 3.62 (dd, 1H, *J* = 11.0, 1.9 Hz, Glc-H-6b), 3.51 (ddd, 1H, *J* = 10.6, 8.8, 5.6 Hz, Xyl-H-4), 3.47 (dt, 1H, *J* = 9.5, 6.9 Hz, OCH_2_(CH_2_)_6_CH_3_), 3.42‒3.36 (m, 2H, Glc-H-2, Xyl-H-3), 3.28 (ddd, 1H, *J* = 9.6, 3.2, 1.8 Hz, Glc-H-5), 3.08 (dd, 1H, *J* = 9.1, 8.0 Hz, Xyl-H-2), 3.01 (app t, 1H, *J* = 11.4 Hz, Xyl-H-5b), 2.19 (s, 3H, COCH_3_), 2.11 (s, 3H, COCH_3_), 2.04 (s, 3H, COCH_3_), 1.96 (s, 3H, COCH_3_), 1.67–1.59 (m, 2H, OCH_2_CH_2_(CH_2_)_5_CH_3_), 1.40–1.16 (m, 10H, OCH_2_CH_2_(CH_2_)_5_CH_3_), 1.19 (d, 3H, *J* = 6.3 Hz, Rha-H-6), 1.04 (d, 3H, *J* = 6.6 Hz, Fuc-H-6), 0.86 (t, 3H, *J* = 7.1 Hz, O(CH_2_)_7_CH_3_); ^13^C NMR (176 MHz; CDCl_3_): δ 171.2 (COCH_3_), 170.2 (COCH_3_), 170.0 (COCH_3_), 169.9(5) (COCH_3_), 138.4 (Ar), 138.2 (Ar), 138.1(5) (Ar), 138.0 (Ar), 137.9 (Ar), 128.5 (Ar), 128.4 (Ar), 128.3(8) (Ar), 128.3(4) (Ar), 128.3 (Ar), 127.9 (Ar), 127.8(9) (Ar), 127.8 (Ar), 127.7(5) (Ar), 127.7 (Ar), 127.6(4) (Ar), 127.6(2) (Ar), 127.4 (Ar), 103.5 (Glc-C-1), 103.2 (Xyl-C-1), 99.2 (Rha-C-1, *J*_C-H_ = 177.4 Hz), 98.1 (Fuc-C-1), 84.0 (Xyl-C-3), 82.6 (Glc-C-2), 82.3 (Xyl-C-2), 78.5 (Xyl-C-4), 75.7 (ArCH_2_), 75.6 (Glc-C-3), 75.4 (Fuc-C-3), 75.1 (ArCH_2_), 75.0, 74.8 (Glc-C-4 & Glc-C-5), 74.5 (ArCH_2_), 73.4 (ArCH_2_ × 2, Fuc-C-4), 70.8 (Rha-C-4), 70.2 (OCH_2_(CH_2_)_6_CH_3_), 69.9 (Rha-C-2), 69.2 (Fuc-C-2), 69.0 (Rha-C-3), 67.6 (Glc-C-6), 66.8 (Rha-C-5), 64.6 (Fuc-C-5), 64.0 (Xyl-C-5), 31.8 (OCH_2_(CH_2_)_6_CH_3_), 29.7 (OCH_2_(CH_2_)_6_CH_3_), 29.4 (OCH_2_(CH_2_)_6_CH_3_), 29.2 (OCH_2_(CH_2_)_6_CH_3_), 26.2 (OCH_2_(CH_2_)_6_CH_3_), 22.6 (OCH_2_(CH_2_)_6_CH_3_), 20.9 (COCH_3_), 20.8(8) (COCH_3_), 20.8 (COCH_3_), 20.7 (COCH_3_), 17.8 (Rha-C-6), 16.0 (Fuc-C-6), 14.1 (O(CH_2_)_7_CH_3_); HRMS (ESI) Calc. for [M + Na]^+^ C_74_H_98_NaO_22_: 1357.6129; Found 1357.6142.

**Octyl 2,3,4-tri-*O*-acetyl-α-D-rhamnopyranosyl-(1→3)-[2,3-di-*O*-benzyl-4,6-*O*-di-*tert*-butylsilylene-α-D-galactopyranosyl-(1→2)]-4-*O*-acetyl-α-L-fucopyranosyl-(1→3)-[2,3,4-tri-*O*-benzyl-β-D-xylopyranosyl-(1→4)]-2,6-di-*O*-benzyl-β-D-glucopyranoside (S27):** To a stirred solution of acceptor **S26** (49.0 mg, 36.7 µmol) and donor **S4**^11^ (44.1 mg, 72.6 µmol) in dry Et_2_O (5.0 mL) was added oven-dried molecular sieves (0.7 g, 4Å, powder) under an Ar atmosphere. After stirring at room temperature for 40 min, methyl trifluoromethanesulfonate (0.05 mL, 442 µmol) was added drop-wise at room temperature. The reaction mixture was stirred at room temperature for 52 h before triethylamine was added. The solution was filtered through Celite and the filtrate was concentrated. The crude residue was purified via flash chromatography (3:1 hexanes–EtOAc) to give **S27** (54.8 mg, 82%) as a colourless film. *R*_f_ 0.34 (3:1 hexanes–EtOAc); [α]ᴅ –1.42 (*c* 1.3, CHCl_3_); ^1^H NMR (700 MHz; CDCl_3_): δ 7.39–7.16 (m, 33H, Ar), 7.05–7.01 (m, 2H, Ar), 5.48 (d, 1H, *J* = 3.5 Hz, Fuc-H-1), 5.31 (dd, 1H, *J* = 3.1, 1.9 Hz, Rha-H-2), 5.17 (d, 1H, *J* = 3.6 Hz, Gal-H-1), 5.12–5.09 (m, 2H, Fuc-H-4, Rha-H-3), 5.02 (d, 1H, *J* = 1.9 Hz, Rha-H-1), 4.97 (d, 1H, *J* = 12.5 Hz, ArCH_2_), 4.94‒4.90 (m, 2H, ArCH_2_, Rha-H-4), 4.87 (app q, 1H, *J* = 6.8 Hz, Fuc-H-5), 4.83, 4.81 (ABq, 2H, *J*_AB_ = 10.9 Hz, ArCH_2_), 4.76 (d, 1H, *J* = 11.1 Hz, ArCH_2_), 4.71‒4.53 (m, 8H, ArCH_2_, Fuc-H-3), 4.49‒4.42 (m, 3H, ArCH_2_, Xyl-H-1), 4.35 (d, 1H, *J* = 7.6 Hz, Glc-H-1), 4.23 (d, 1H, *J* = 12.6 Hz, ArCH_2_), 4.15–4.05 (m, 3H, Gal-H-4, Fuc-H-2, Xyl-H-5a), 4.02 (dd, 1H, *J* = 10.0, 3.4 Hz, Gal-H-2), 3.99 (app t, 1H, *J* = 9.3 Hz, Glc-H-4), 3.94–3.84 (m, 4H, Glc-H-6a, Glc-H-3, OCH_2_(CH_2_)_6_CH_3_, Rha-H-5), 3.83 (dd, 1H, *J* = 10.1, 3.0 Hz, Gal-H-3), 3.69–3.63 (m, 3H, Gal-H-6a, Glc-H-6b, Xyl-H-4), 3.44 (app t, 2H, *J* = 9.0 Hz, Xyl-H-3), 3.39–3.32 (m, 3H, OCH_2_(CH_2_)_6_CH_3_, Glc-H-5, Glc-H-2), 3.20 (app s, 1H, Gal-H-5), 3.16–3.09 (m, 3H, Xyl-H-5b, Gal-H-6b, Xyl-H-2), 2.25 (s, 3H, COCH_3_), 2.00 (s, 3H, COCH_3_), 1.86 (s, 3H, COCH_3_), 1.63 (s, 3H, COCH_3_), 1.56–1.49 (m, 2H, OCH_2_CH_2_(CH_2_)_5_CH_3_), 1.28–1.06 (m, 10H, OCH_2_CH_2_(CH_2_)_5_CH_3_), 1.16 (d, 3H, *J* = 6.2 Hz, Rha-H-6), 1.08 (d, 3H, *J* = 6.7 Hz, Fuc-H-6), 0.98 (s, 9H, C(CH_3_)_3_), 0.97 (s, 9H, C(CH_3_)_3_), 0.83 (t, 3H, *J* = 7.3 Hz, O(CH_2_)_7_CH_3_); ^13^C NMR (176 MHz; CDCl_3_): δ 171.3 (COCH­_3_), 170.1 (COCH­_3_), 169.6 (COCH­_3_), 169.4 (COCH­_3_), 139.7 (Ar), 139.0 (Ar), 138.6 (Ar), 138.5 (Ar), 138.3 (Ar), 138.2 (Ar), 138.1 (Ar), 128.5 (Ar), 128.4(8) (Ar), 128.4 (Ar), 128.3(5) (Ar), 128.1 (Ar), 128.0 (Ar), 127.9(5) (Ar), 127.9 (Ar), 127.8 (Ar), 127.7(3) (Ar), 127.7 (Ar), 127.6(5) (Ar), 127.6 (Ar), 127.4(2) (Ar), 127.4 (Ar), 127.0 (Ar), 126.9 (Ar), 126.8 (Ar), 125.2 (Ar), 103.5, 103.4 (Glc-C-1 & Xyl-C-1), 100.4 (Rha-C-1), 98.4 (Gal-C-1), 96.7 (Fuc-C-1), 84.0 (Xyl-C-3), 82.4, 82.3 (Glc-C-2 & Xyl-C-2), 78.7 (Xyl-C-4), 77.4 (Gal-C-3), 76.9 (Fuc-C-3), 75.7 (ArCH_2_), 75.2(3) (ArCH_2_), 75.2 (Glc-C-4), 75.0 (Glc-C-5), 74.7 (Fuc-C-4), 73.9 (Gal-C-2), 73.7 (Glc-C-3), 73.4 (ArCH_2_), 73.3 (ArCH_2_), 72.3 (ArCH_2_), 72.2 (ArCH_2_), 71.3, 71.1 (Gal-C-4 & Fuc-C-2), 70.8 (ArCH_2_), 70.7 (Rha-C-4), 69.9 (OCH_2_(CH_2_)_6_CH_3_), 69.5, 69.4(7) (Rha-C-2 & Rha-C-3), 67.7 (Glc-C-6), 67.5 (Gal-C-5), 67.0 (Rha-C-5), 66.5 (Gal-C-6), 64.6 (Fuc-C-5), 64.0 (Xyl-C-5), 31.8 (OCH_2_(CH_2_)_6_CH_3_), 29.4 (OCH_2_(CH_2_)_6_CH_3_), 29.2 (OCH_2_(CH_2_)_6_CH_3_), 28.0 (C(CH_3_)_3_), 27.8 (C(CH_3_)_3_), 27.63 (C(CH_3_)_3_), 27.3 (C(CH_3_)_3_), 26.1 (OCH_2_(CH_2_)_6_CH_3_), 23.2 (OCH_2_(CH_2_)_6_CH_3_), 22.6 (OCH_2_(CH_2_)_6_CH_3_), 21.2 (COCH_3_), 20.9 (COCH_3_), 20.6 (COCH_3_), 20.5(6) (C(CH_3_)_3_), 20.4 (COCH_3_), 19.7 (C(CH_3_)_3_), 17.9 (Rha-C-6), 16.2 (Fuc-C-6), 14.1 (CH_2_)_7_CH_3_); HRMS (ESI) Calc. for [M + Na]^+^ C_102_H_132_NaO_27_Si: 1839.8617; Found 1839.8635.

**Octyl 2,3,4-tri-*O*-acetyl-α-D-rhamnopyranosyl-(1→3)-[2,3-di-*O*-benzyl-α-D-galactopyranosyl-(1→2)]-4-*O*-acetyl-α-L-fucopyranosyl-(1→3)-[2,3,4-tri-*O*-benzyl-β-D-xylopyranosyl-(1→4)]-2,6-di-*O*-benzyl-β-D-glucopyranoside (S28)**: To a stirred solution of **S27** (56.2 mg, 30.9 µmol) in THF–pyridine (1:1, 3.2 mL THF, 3.2 mL pyridine) under an Ar atmosphere was added HF∙pyridine (0.8 mL, hydrogen fluoride ∼70%, pyridine ∼30%) at 0 ˚C. The reaction mixture was slowly warmed to room temperature and stirred for a total of 20 h. The reaction mixture was diluted with EtOAc (15 mL) and poured into a saturated solution of NaHCO_3_ (aq). The aqueous layer was extracted with EtOAc (10 mL × 3) and the combined organic layers were dried over Na_2_SO_4_, filtered and then the filtrate was concentrated. The crude residue was purified via flash chromatography (3:2 EtOAc–hexanes) to give **S28** (40.2 mg, 78%) as a colourless film. *R*_f_ 0.36 (3:2 EtOAc–hexanes); [α]ᴅ –8.6 (*c* 2.6, CHCl_3_); ^1^H NMR (700 MHz; CDCl_3_): δ 7.37–7.18 (m, 33H, Ar), 7.17–7.13 (m, 2H, Ar), 5.54 (d, 1H, *J* = 3.5 Hz, Fuc-H-1), 5.31 (dd, 1H, *J* = 3.2, 1.8 Hz, Rha-H-2), 5.21 (d, 1H, *J* = 2.7 Hz, Gal-H-1), 5.13‒5.09 (m, 2H, Fuc-H-4, Rha-H-3), 5.01 (d, 1H, *J* = 1.8 Hz, Rha-H-1), 4.97‒4.92 (m, 2H, ArCH_2_, Rha-H-4), 4.86–4.80 (m, 3H, ArCH_2_, Fuc-H-5), 4.80 (d, 1H, *J* = 12.6 Hz, ArCH_2_), 4.77 (d, 1H, *J* = 11.1 Hz, ArCH_2_), 4.70 (d, 1H, *J* = 11.3 Hz, ArCH_2_), 4.69 (d, 1H, *J* = 11.2 Hz, ArCH_2_), 4.65 (d, 1H, *J* = 12.0 Hz, ArCH_2_), 4.62 (d, 1H, *J* = 11.7 Hz, ArCH_2_), 4.59 (d, 1H, *J* = 11.3 Hz, ArCH_2_), 4.57–4.52 (m, 3H, ArCH_2_, Fuc-H-3), 4.50‒4.46 (m, 2H, ArCH_2_, Xyl-H-1), 4.40 (d, 1H, *J* = 7.6 Hz, Glc-H-1), 4.34 (d, 1H, *J* = 12.4 Hz, ArCH_2_), 4.14 (dd, 1H, *J* = 10.1, 3.6 Hz, Fuc-H-2), 4.08 (dd, 1H, *J* = 11.5, 5.6 Hz, Xyl-H-5a), 4.02 (app t, 1H, *J* = 9.1 Hz, Glc-H-4), 3.96 (app t, 1H, *J* = 8.6 Hz, Glc-H-3), 3.94–3.80 (m, 5H, Glc-H-6a, Rha-H-5, OCH_2_(CH_2_)_6_CH_3_, Gal-H-2, Gal-H-3), 3.76 (app br s, 1H, Gal-H-4), 3.69–3.63 (m, 2H, Xyl-H-4, Glc-H-6b), 3.47–3.42 (m, 3H, Gal-H-5, Xyl-H-3, Glc-H-2), 3.42–3.35 (m, 2H, OCH_2_(CH_2_)_6_CH_3_, Glc-H-5), 3.34–3.29 (m, 2H, Gal-H-6a, Gal-H-6b), 3.17‒3.11 (m, 2H, Xyl-H-2, Xyl-H-5b), 2.21 (s, 3H, COCH_3_), 2.01 (s, 3H, COCH_3_), 1.87 (s, 3H, COCH_3_), 1.69 (s, 3H, COCH_3_), 1.58–1.52 (m, 2H, OCH_2_CH_2_(CH_2_)_5_CH_3_), 1.30–1.13 (m, 10H, OCH_2_CH_2_(CH_2_)_5_CH_3_), 1.18 (d, 3H, *J* = 6.2 Hz, Rha-H-6), 1.08 (d, 3H, *J* = 6.6 Hz, Fuc-H-6), 0.84 (t, 3H, *J* = 7.3 Hz, O(CH_2_)_7_CH_3_); ^13^C NMR (176 MHz; CDCl_3_): δ 171.3 (COCH_3_), 170.1 (COCH_3_), 169.9 (COCH_3_), 169.6 (COCH_3_), 139.3 (Ar), 138.6 (Ar), 138.5 (Ar), 138.3 (Ar), 138.2(7) (Ar), 138.0 (Ar), 128.6 (Ar), 128.5 (Ar), 128.4(8) (Ar), 128.4 (Ar), 128.3(7) (Ar), 128.3 (Ar), 128.2 (Ar), 128.1 (Ar), 128.0(4) (Ar), 128.0 (Ar), 127.9 (Ar), 127.8 (Ar), 127.7 (Ar), 127.6 (Ar), 127.4 (Ar), 127.2 (Ar), 127.1 (Ar), 126.8 (Ar), 125.7(8) (Ar), 103.4 (Xyl-C-1), 103.2 (Glc-C-1), 100.3 (Rha-C-1), 97.6 (Gal-C-1), 96.3 (Fuc-C-1), 84.0 (Xyl-C-3), 82.4 (Xyl-C-2), 82.2 (Glc-C-2), 78.7 (Xyl-C-4), 76.9 (Gal-C-3), 76.7 (Fuc-C-3), 75.6 (ArCH_2_), 75.3 (Glc-C-4, Gal-C-2), 75.2 (ArCH_2_), 75.0 (Glc-C-5), 74.6 (Fuc-C-4), 73.9 (Glc-C-3), 73.4 (ArCH_2_), 73.3 (ArCH_2_), 72.6 (ArCH_2_), 72.4 (ArCH_2_), 72.3 (ArCH_2_), 71.8 (Fuc-C-2), 70.6 Rha-C-4), 69.9 (OCH_2_(CH_2_)_6_CH_3_), 69.6, 69.5, 69.4, 69.2 (Gal-C-5 & Rha-C-2 & Rha-C-3 & Gal-C-4), 67.8 (Glc-C-6), 67.0 (Rha-C-5), 64.6 (Fuc-C-5), 64.1 (Xyl-C-5), 62.6 (Gal-C-6), 31.8 (OCH_2_(CH_2_)_6_CH_3_), 29.7 (OCH_2_(CH_2_)_6_CH_3_), 29.4 (OCH_2_(CH_2_)_6_CH_3_), 29.2 (OCH_2_(CH_2_)_6_CH_3_), 26.1 (OCH_2_(CH_2_)_6_CH_3_), 22.6 (OCH_2_(CH_2_)_6_CH_3_), 21.0 (COCH_3_), 20.9 (COCH_3_), 20.7 (COCH_3_), 20.5 (COCH_3_), 17.9 (Rha-C-6), 16.2 (Fuc-C-6), 14.1(O(CH_2_)_7_CH_3_); HRMS (ESI) Calc. for [M + Na]^+^ C_94_H_116_NaO_27_: 1699.7596; Found 1699.7607.

**Octyl α-ᴅ-rhamnopyranosyl-(1→3)-[α-D-galactopyranosyl-(1→2)]-α-D-fucopyranosyl-(1→3)-[β-D-xylopyranosyl-(1→4)]-β-D-glucopyranoside (7)**: To a stirred solution of **S28** (61.8 mg, 36.8 µmol) in CH_3_OH (6.0 mL) was added NaOH (48.1 mg, 1.20 mmol). The reaction mixture was stirred at room temperature for 24 h. Amberlite® IR-120 (H^+^) cation exchange resin was added, the mixture filtered and then the filtrate was concentrated. The crude residue was purified via flash chromatography (12:1 CH_2_Cl_2_–CH_3_OH). The product was dissolved in THF–H_2_O (1:1, 2.5 mL THF, 2.5 mL H_2_O) and 20% Pd(OH)_2_/C (13.2 mg) was added under an Ar atmosphere. The reaction mixture was stirred at room temperature, under a H_2_ atmosphere, for 23 h and was then filtered and concentrated. The crude residue was purified via Iatrobead chromatography (CH_2_Cl_2_:CH_3_OH→CH_3_OH, 10% stepwise gradient) to give **7** as a white film (4.5 mg, 14%). *R*_f_ 0.54 (4:1:1:1 EtOAc–CH_3_OH–H_2_O–AcOH); [α]ᴅ –6.1 (*c* 0.2, CH_3_OH); ^1^H NMR (700 MHz; D_2_O): δ 5.63 (d, 1H, *J* = 3.9 Hz, Fuc-H-1), 5.22 (d, 1H, *J* = 4.0 Hz, Gal-H-1), 4.99 (d, 1H, *J* = 1.6 Hz, Rha-H-1), 4.77–4.71 (m, 1H, Fuc-H-5), 4.46 (d, 1H, *J* = 8.0 Hz, Glc-H-1), 4.43 (d, 1H, *J* = 7.9 Hz, Xyl-H-1), 4.22 (dd, 1H, *J* = 10.5, 3.2 Hz, Fuc-H-3), 4.10 (dd, 1H, *J* = 3.5, 1.6 Hz, Rha-H-2), 4.07–4.02 (m, 3H, Fuc-H-2, Gal-H-4, Gal-H-5), 4.01–3.95 (m, 2H, Glc-H-6a, Xyl-H-5a), 3.94–3.76 (m, 8H, OCH_2_(CH_2_)_6_CH_3_, Glc-H-3, Gal-H-3, Rha-H-3, Gal-H-2, Glc-H-6b, Rha-H-5, Fuc-H-4), 3.74–3.66 (m, 4H, Gal-H-6a, Gal-H-6b, Glc-H-4, OCH_2_(CH_2_)_6_CH_3_), 3.62–3.55 (m, 2H, Glc-H-5, Xyl-H-4), 3.49 (app t, 1H, *J* = 9.7 Hz, Rha-H-4), 3.47‒3.44 (m, 2H, Xyl-H-3, Glc-H-2), 3.29 (app t, 1H, *J* = 11.1 Hz, Xyl-H-5b), 3.15 (dd, 1H, *J* = 9.4, 7.9 Hz, Xyl-H-2), 1.63 (app pent, 2H, *J* = 6.9 Hz, OCH_2_CH_2_(CH_2_)_5_CH_3_), 1.40–1.27 (m, 10H, OCH_2_CH_2_(CH_2_)_5_CH_3_), 1.30 (d, 3H, *J* = 6.3 Hz, Rha-H-6), 1.20 (d, 3H, *J* = 6.7 Hz, Fuc-H-6), 0.87 (t, 3H, *J* = 6.8 Hz, O(CH_2_)_7_CH_3_); ^13^C NMR (176 MHz; D_2_O): δ 104.1 (Rha-C-1), 103.5 (Xyl-C-1), 103.2 (Glc-C-1), 100.3 (Gal-C-1, *J*_C-H_ = 176.8 Hz), 98.1 (Fuc-C-1), 79.2 (Fuc-C-3), 76.5 (Xyl-C-3), 76.0, 75.9 (Glc-C-2 & Glc-C-5), 75.7 (Glc-C-3), 74.8 (Glc-C-4), 74.6 (Xyl-C-2), 73.3 (Fuc-C-4), 72.9 (Rha-C-4), 72.0 (Gal-C-5), 71.8 (OCH_2_(CH_2_)_6_CH_3_), 71.5 (Fuc-C-2), 71.1 (Rha-C-3), 71.0 (Rha-C-2), 70.6, 70.5 (Xyl-C-4 & Gal-C-3), 69.9 (Gal-C-4), 69.8 (Rha-C-5), 69.3 (Gal-C-2), 67.2 (Fuc-C-5), 65.7 (Xyl-C-5), 61.8 (Gal-C-6), 60.4 (Glc-C-6), 31.9 (OCH_2_(CH_2_)_6_CH_3_), 29.6 (OCH_2_(CH_2_)_6_CH_3_), 29.3 (OCH_2_(CH_2_)_6_CH_3_), 29.2 (OCH_2_(CH_2_)_6_CH_3_), 25.9 (OCH_2_(CH_2_)_6_CH_3_), 22.9 (OCH_2_(CH_2_)_6_CH_3_), 17.5 (Rha-C-6), 15.7 (Fuc-C-6), 14.2

***Synthesis of 9***

**Octyl 2,3,4-tri-*O*-benzyl-β-D-xylopyranosyl-(1→4)-[3-*O*-allyl-2-*O*-(4-methoxybenzyl)-α-L-fucopyranosyl-(1→3)]-2,6-di-*O*-benzyl-β-D-glucopyranoside (S29):** To a stirred solution of **S21** (254.5 mg, 208.0 µmol) in CH_3_OH (4.0 mL) was added NaOH (105.2 mg, 2.630 mmol). The reaction mixture was stirred at room temperature for 28 h. Amberlite® IR-120 (H^+^) cation exchange resin was added, the mixture filtered and then the filtrate was concentrated. The crude residue was purified via flash chromatography (3:1 hexanes–EtOAc) to give **S29** (210.9 mg, 86%) as a colourless syrup. *R*_f_ 0.40 (3:1 hexanes–EtOAc); [α]ᴅ –13.7 (*c* 7.8, CHCl_3_); ^1^H NMR (700 MHz; CDCl_3_): δ 7.37–7.21 (m, 25H, Ar), 7.06–7.01 (m, 2H, Ar), 6.69–6.64 (m, 2H, Ar), 5.94 (ddt, 1H, *J* = 17.2, 10.6, 5.4 Hz, OCH_2_CH=CH_2_), 5.67 (d, 1H, *J* = 3.7 Hz, Fuc-H-1), 5.29 (app dq, 1H, *J* = 17.2, 1.7 Hz, OCH_2_CH=CH_2_), 5.16 (app dq, 1H, *J* = 10.5, 1.5 Hz, OCH_2_CH=CH_2_), 5.06 (d, 1H, *J* = 11.5 Hz, ArCH_2_), 4.87–4.81 (m, 2H, ArCH_2_), 4.80 (d, 1H, *J* = 11.1 Hz, ArCH_2_), 4.77 (app q, 1H, *J* = 3.7 Hz, Fuc-H-5), 4.72 (d, 1H, *J* = 11.8 Hz, ArCH_2_), 4.69 (d, 1H, *J* = 11.1 Hz, ArCH_2_), 4.67‒4.64 (m, 2H, ArCH_2_), 4.58 (d, 1H, *J* = 11.8 Hz, ArCH_2_), 4.54 (d, 1H, *J* = 11.9 Hz, ArCH_2_), 4.45 (d, 1H, *J* = 11.9 Hz, ArCH_2_), 4.43‒4.39 (m, 2H, ArCH_2_, Xyl-H-1), 4.37 (d, 1H, *J* = 7.8 Hz, Glc-H-1), 4.15–4.12 (m, 1H, OCH_2_CH=CH_2_), 4.08 (app ddt, 1H, *J* = 12.8, 5.7, 1.5 Hz, OCH_2_CH=CH_2_), 4.04 (app t, 1H, *J* = 9.6 Hz, Glc-H-4), 3.97 (app t, 1H, *J* = 9.3 Hz, Glc-H-3), 3.94–3.87 (m, 3H, Fuc-H-3, Glc-H-6a, OCH_2_(CH_2_)_6_CH_3_), 3.77 (dd, 1H, *J* = 4.9, 11.7 Hz, Xyl-H-5a), 3.72 (s, 3H, ArOCH_3_), 3.68 (dd, 1H, *J* = 9.9, 3.7 Hz, Fuc-H-2), 3.67–3.64 (m, 2H, Fuc-H-4, Glc-H-6b), 3.54 (dd, 1H, *J* = 9.2, 7.8 Hz, Glc-H-2), 3.46–3.37 (m, 3H, OCH_2_(CH_2_)_6_CH_3_, Xyl-H-3, Xyl-H-4), 3.34 (ddd, 1H, *J* = 9.8, 3.4, 1.8 Hz, Glc-H-5), 3.09 (app t, 1H, *J* = 8.3 Hz, Xyl-H-2), 2.97 (dd, 1H, *J* = 11.8, 10.0 Hz, Xyl-H-5b), 1.62–1.53 (m, 2H, OCH_2_CH_2_(CH_2_)_5_CH_3_), 1.24 (d, 3H, *J* = 6.7 Hz, Fuc-H-6), 1.35–1.16 (m, 10H, OCH_2_CH_2_(CH_2_)_5_CH_3_), 0.86 (t, 3H, *J* = 7.2 Hz, O(CH_2_)_7_CH_3_); ^13^C NMR (176 MHz; CDCl_3_): δ 159.0 (Ar), 138.8 (Ar), 138.5(Ar), 138.3 (Ar), 138.2(3) (Ar), 138.2(2) (Ar), 135.2 (OCH_2_CH=CH_2_), 130.5 (Ar), 129.5 (Ar), 128.5 (Ar), 128.42 (Ar × 2), 128.4 (Ar), 128.3 (Ar), 128.0 (Ar), 127.9 (Ar), 127.8(9) (Ar), 127.7 (Ar), 127.6(6) (Ar), 127.6(4) (Ar), 127.6 (Ar), 127.1 (Ar), 126.6 (Ar), 116.4 (OCH_2_CH=CH_2_), 113.6 (Ar), 103.7 (Glc-C-1), 103.2 (Xyl-C-1), 96.8 (Fuc-C-1), 84.1 (Xyl-C-3), 83.1 (Glc-C-2), 82.7 (Xyl-C-2), 78.3 (Xyl-C-4), 77.8 (Fuc-C-3), 75.8 (ArCH_2_), 75.3 (Glc-C-5), 75.2 (ArCH_2_), 74.7 (Glc-C-4), 74.3 (Glc-C-3), 74.1 (Fuc-C-2), 73.6 (ArCH_2_), 73.4 (ArCH_2_), 73.3 (ArCH_2_), 72.4 (ArCH_2_), 71.1 (OCH_2_CH=CH_2_), 70.7 (Fuc-C-4), 70.2 (OCH_2_(CH_2_)_6_CH_3_), 67.8 (Glc-C-6), 64.5 (Fuc-C-5), 64.0 (Xyl-C-5), 55.2 (ArOCH_3_), 31.8 (OCH_2_(CH_2_)_6_CH_3_), 29.7 (OCH_2_(CH_2_)_6_CH_3_), 29.4 (OCH_2_(CH_2_)_6_CH_3_), 29.2 (OCH_2_(CH_2_)_6_CH_3_), 26.1 (OCH_2_(CH_2_)_6_CH_3_), 22.7 (OCH_2_(CH_2_)_6_CH_3_), 16.2 (Fuc-C-6), 14.1 (O(CH_2_)_7_CH_3_); HRMS (ESI) Calc. for [M + Na]^+^ C_71_H_88_NaO_15_: 1203.6015; Found 1203.6015.

**Octyl 2,3,4-tri-*O*-acetyl-β-D-xylopyranosyl-(1→4)-3-*O*-allyl-2-*O*-(4-methoxybenzyl)-α-L-fucopyranosyl-(1→3)-[2,3,4-tri-*O*-benzyl-β-D-xylopyranosyl-(1→4)]-2,6-di-*O*-benzyl-β-D-glucopyranoside (S30):** To a stirred solution of acceptor **S29** (146.0 mg, 123.6 µmol) and donor **S15** (83.8 mg, 187 µmol) in dry CH_2_Cl_2_ (4.0 mL) was added oven-dried molecular sieves (0.4 g, 4Å, powder) under an Ar atmosphere. After stirring at room temperature for 30 min, the solution was chilled to –30 ˚C and trifluoromethanesulfonic acid (0.110 µL, 1.25 µmol) was added drop-wise. The resulting solution was stirred for 1 h at –30 ˚C before triethylamine was added. The solution was filtered through Celite and the filtrate was concentrated. The crude residue was purified via flash chromatography (3:1:1 hexanes–EtOAc–CH_2_Cl_2_) to give **S30** (145.9 mg, 82%) as a colourless syrup. *R*_f_ 0.56 (2.5:1:1 hexanes–EtOAc–CH_2_Cl_2_); [α]ᴅ –34.1 (*c* 2.5, CHCl_3_); ^1^H NMR (700 MHz; CDCl_3_): δ 7.38–7.21 (m, 25H, Ar), 7.21–7.18 (m, 2H, Ar), 7.09–7.01 (m, 2H, Ar), 6.65–6.61 (m, 2H, Ar), 5.94 (dddd, 1H, *J* = 17.3, 10.5, 5.6, 4.9 Hz, OCH_2_CH=CH_2_), 5.55 (d, 1H, *J* = 3.7 Hz, Fuc-H-1), 5.31 (app dq, 1H, *J* = 17.3, 1.8 Hz, OCH_2_CH=CH_2_), 5.13 (app dq, 1H, *J* = 10.5, 1.5 Hz, OCH_2_CH=CH_2_), 5.01 (app t, 1H, *J* = 6.0 Hz, Xyl’-H-3), 4.98 (d, 1H, *J* = 11.5 Hz, ArCH_2_), 4.95 (dd, 1H, *J* = 6.1, 4.4 Hz, Xyl’-H-2), 4.84–4.80 (m, 3H, ArCH_2_, Xyl’-H-4), 4.78 (d, 1H, *J* = 11.1 Hz, ArCH_2_), 4.69 (d, 1H, *J* = 11.8 Hz, ArCH_2_), 4.67–4.62 (m, 5H, ArCH_2_, Fuc-H-5, Xyl’-H-1), 4.59–4.54 (m, 3H, ArCH_2_), 4.41‒4.37 (m, 2H, ArCH_2_, Xyl-H-1), 4.36‒4.32 (m, 1H, Xyl’-H-5a), 4.33 (d, 1H, *J* = 7.9 Hz, Glc-H-1), 4.11 (app ddt, 1H, *J* = 12.4, 5.6, 1.4 Hz, OCH_2_CH=CH_2_), 4.02 (app t, 1H, *J* = 9.6 Hz, Glc-H-4), 3.97 (app ddt, 1H, *J* = 12.4, 4.9, 1.6 Hz, OCH_2_CH=CH_2_), 3.93 (dd, 1H, *J* = 10.3, 2.8 Hz, Fuc-H-3), 3.91–3.84 (m, 3H, Glc-H-3, OCH_2_(CH_2_)_6_CH_3_, Glc-H-6a), 3.80 (dd, 1H, *J* = 11.5, 4.5 Hz, Xyl-H-5a), 3.75 (dd, 1H, *J* = 10.2, 3.7 Hz, Fuc-H-2), 3.69 (s, 3H, ArOCH_3_), 3.66–3.61 (m, 2H, Fuc-H-4, Glc-H-6b), 3.49 (dd, 1H, *J* = 9.2, 7.8 Hz, Glc-H-2), 3.44–3.37 (m, 4H, OCH_2_(CH_2_)_6_CH_3_, Xyl’-H-5b, Xyl-H-3, Xyl-H-4), 3.30 (ddd, 1H, *J* = 9.8, 3.4, 1.9 Hz, Glc-H-5), 3.05 (dd, 1H, *J* = 9.0, 7.9 Hz, Xyl-H-2), 2.94 (dd, 1H, *J* = 11.8, 10.3 Hz, Xyl-H-5b), 2.08 (s, 3H, COCH_3_), 2.06 (s, 3H, COCH_3_), 1.85 (s, 3H, COCH_3_), 1.59–1.50 (m, 2H, , OCH_2_CH_2_(CH_2_)_5_CH_3_), 1.33–1.15 (m, 10H, OCH_2_CH_2_(CH_2_)_5_CH_3_), 1.13 (d, 3H, *J* = 6.6 Hz, Fuc-H-6), 0.85 (t, 3H, *J* = 7.2 Hz, O(CH_2_)_7_CH_3_); ^13^C NMR (176 MHz; CDCl_3_): δ 169.9 (COCH_3_), 169.8 (COCH_3_), 169.4 (COCH_3_), 158.9 (Ar), 138.8 (Ar), 138.4 (Ar), 138.2 (Ar), 138.1(7) (Ar), 138.1(6) (Ar), 135.4 (OCH_2_CH=CH_2_), 130.7 (Ar), 129.4 (Ar), 128.5 (Ar), 128.4 (Ar), 128.3(8) (Ar), 128.3(5) (Ar), 128.2 (Ar), 128.1 (Ar), 127.9 (Ar), 127.8 (Ar), 127.7(6) (Ar), 127.7 (Ar), 127.6(3) (Ar), 127.6(1) (Ar), 127.5(8) (Ar), 127.5(6) (Ar), 127.5(1) (Ar), 127.1 (Ar), 126.7 (Ar), 116.09 (OCH_2_CH=CH_2_), 113.5 (Ar), 103.7 (Glc-C-1), 103.1 (Xyl-C-1), 100.0 (Xyl’-C-1, *J*_C-H_ = 166.9 Hz), 97.1 (Fuc-C-1), 84.0 (Xyl-C-3), 83.0 (Glc-C-2), 82.6 (Xyl-C-2), 79.2 (Fuc-C-4), 78.3 (Xyl-C-4), 77.8 (Fuc-C-3), 75.9 (ArCH_2_), 75.2 (Glc-C-5), 75.1 (ArCH_2_), 74.6 (Glc-C-4), 74.3 (Glc-C-3), 73.9 (Fuc-C-2), 73.6 (ArCH_2_), 73.3 (ArCH_2_), 73.2 (ArCH_2_), 72.7 (ArCH_2_), 70.8 (OCH_2_CH=CH_2_), 70.2 (OCH_2_(CH_2_)_6_CH_3_), 69.2 (Xyl’-C-3), 69.1 (Xyl’-C-2), 68.2 (Xyl’-C-4), 67.8 (Glc-C-6), 65.2 (Fuc-C-5), 63.9 (Xyl-C-5), 60.6 (Xyl’-C-5), 55.1 (ArOCH_3_), 31.8 (OCH_2_(CH_2_)_6_CH_3_), 29.6 (OCH_2_(CH_2_)_6_CH_3_), 29.4 (OCH_2_(CH_2_)_6_CH_3_), 29.2 (OCH_2_(CH_2_)_6_CH_3_), 26.1 (OCH_2_(CH_2_)_6_CH_3_), 22.6 (OCH_2_(CH_2_)_6_CH_3_), 20.9 (COCH_3_), 20.8(5) (COCH_3_), 20.5 (COCH_3_), 16.4 (Fuc-C-6), 14.1 (O(CH_2_)_7_CH_3_); HRMS (ESI) Calc. for [M + Na]^+^ C_82_H_102_NaO_22_: 1461.6755; Found 1461.6765.

**Octyl 2,3,4-tri-*O*-acetyl-β-D-xylopyranosyl-(1→4)-2-*O*-(4-methoxybenzyl)-α-L-fucopyranosyl-(1→3)-[2,3,4-tri-*O*-benzyl-β-D-xylopyranosyl-(1→4)]-2,6-di-*O*-benzyl-β-D-glucopyranoside (S31):** A solution of **S30** (84.1 mg, 58.4 µmol) in dry THF (3.0 mL) was degassed via vacuum and (1,5-Cyclooctadiene)bis(methyldiphenyl-phosphine)iridium(I) hexafluorophosphate (7.9 mg, 9.3 µmol) was added under an Ar atmosphere. The resulting mixture was stirred at 0 ˚C for 15 min before the catalyst was activated with hydrogen (stirring for 2 min under a hydrogen atmosphere). Excess hydrogen was removed by three cycles of vacuum purging with Ar. The reaction mixture was stirred at room temperature for 22 h under an Ar atmosphere and then concentrated. The residue was dissolved in acetone**–**water (10:1, 2.5 mL) and HgO (21.6 mg, 82.6 µmol) and HgCl_2_ (19.2 mg, 70.7 µmol) were added. The reaction mixture was stirred at room temperature for 2.5 h and then concentrated. The residue was diluted with EtOAc (40 mL) and was washed with 10% KI (aq), saturated Na_2_S_2_O_3_ (aq), and water. The aqueous layers were extracted with EtOAc (80 mL) and the combined organic layers were dried over Na_2_SO_4_, filtered and then the filtrate was concentrated. The crude residue was purified via flash chromatography (2:1 hexanes**–**EtOAc→1:1 hexanes**–**EtOAc) to give **S31** (64.5 mg, 79%) as a syrup. *R*_f_ 0.60 (1:1 hexanes**–**EtOAc); [α]ᴅ **–**55.4 (*c* 1.1, CHCl_3_); ^1^H NMR (700 MHz; CDCl_3_): δ 7.37**–**7.23 (m, 23H, Ar), 7.23**–**7.19 (m, 2H, Ar), 7.12**–**7.08 (m, 2H, Ar), 6.75**–**6.70 (m, 2H, Ar), 5.60 (d, 1H, *J* = 3.6 Hz, Fuc-H-1), 5.15 (app t, 1H, *J* = 9.1 Hz, Xyl’-H-3), 5.00 (d, 1H, *J* = 11.5 Hz, ArCH_2_), 4.99‒4.92 (m, 2H, Xyl’-H-2, Xyl’-H-4), 4.82**–**4.75 (m, 4H, ArCH_2_, Fuc-H-5), 4.67 (d, 1H, *J* = 11.5 Hz, ArCH_2_), 4.67 (d, 1H, *J* = 11.2 Hz, ArCH_2_), 4.64**–**4.60 (m, 3H, ArCH_2_), 4.56 (d, 1H, *J* = 11.8 Hz, ArCH_2_), 4.46 (d, 1H, *J* = 7.4 Hz, Xyl’-H-1), 4.41**–**4.37 (m, 3H, ArCH_2_, Xyl-H-1), 4.34 (d, 1H, *J* = 7.7 Hz, Glc-H-1), 4.12 (app td, 1H, *J* = 9.9, 3.1 Hz, Fuc-H-3), 4.05 (dd, 1H, *J* = 11.7, 5.4 Hz, Xyl’-H-5a), 4.01 (app t, 1H, *J* = 9.6 Hz, Glc-H-4), 3.93 (dd, 1H, *J* = 11.7, 5.2 Hz, Xyl-H-5a), 3.90**–**3.83 (m, 3H, Glc-H-3, OCH_2_(CH_2_)_6_CH_3_, Glc-H-6a), 3.74 (s, 3H, ArOCH_3_), 3.63 (dd, 1H, *J* = 11.0, 1.8 Hz, Glc-H-6b), 3.56 (dd, 1H, *J* = 3.2, 1.3 Hz, Fuc-H-4), 3.52**–**3.46 (m, 2H, Fuc-H-2, Glc-H-2), 3.44**–**3.39 (m, 2H, OCH_2_(CH_2_)_6_CH_3_, Xyl-H-4), 3.38 (app t, 1H, *J* = 8.8 Hz, Xyl-H-3), 3.31 (ddd, *J* = 9.8, 3.4, 1.9 Hz, 1H, Glc-H-5), 3.28 (dd, 1H, *J* = 11.8, 9.5 Hz, Xyl’-H-5b), 3.05 (app t, 1H, *J* = 8.4 Hz, Xyl-H-2), 3.00**–**2.93 (m, 2H, Xyl-H-5b, Fuc-3-OH), 2.03 (s, 3H, COCH_3_), 2.03 (s, 3H, COCH_3_), 2.01 (s, 3H, COCH_3_), 1.57**–**1.49 (m, 2H, OCH_2_CH_2_(CH_2_)_5_CH_3_), 1.31**–**1.13 (m, 10H, OCH_2_CH_2_(CH_2_)_5_CH_3_), 1.10 (d, 3H, *J* = 6.7 Hz, Fuc-H-6), 0.84 (t, 3H, *J* = 7.2 Hz, O(CH_2_)_7_CH_3_); ^13^C NMR (176 MHz; CDCl_3_): δ 170.2 (COCH_3_), 169.7 (COCH_3_), 169.3 (COCH_3_), 159.0 (Ar), 138.9 (Ar), 138.4 (Ar), 138.3 (Ar), 138.2 (Ar), 138.1 (Ar), 130.5 (Ar), 129.7 (Ar), 128.5 (Ar), 128.4 (Ar), 128.3(9) (Ar), 128.3 (Ar), 128.2 (Ar), 128.1 (Ar), 127.8 (Ar), 127.7(6) (Ar), 127.6(4) (Ar), 127.6 (Ar), 127.5 (Ar), 127.1 (Ar), 126.6(6) (Ar), 113.5 (Ar), 103.7 (Glc-C-1), 103.2 (Xyl-C-1), 102.3 (Xyl’-C-1), 97.0 (Fuc-C-1), 84.8 (Fuc-C-4), 84.0 (Xyl-C-3), 83.0 (Glc-C-2), 82.5 (Xyl-C-2), 78.5 (Xyl-C-4), 76.0 (ArCH_2_), 75.7 (Fuc-C-2), 75.2 (Glc-C-5), 75.1 (ArCH_2_), 74.8 (Glc-C-4), 74.7 (Glc-C-3), 73.6(7) (ArCH_2_), 73.3 (ArCH_2_), 73.2 (ArCH_2_), 72.4 (ArCH_2_), 72.0 (Xyl’-C-3), 71.5 (Xyl’-C-2), 70.1 (OCH_2_(CH_2_)_6_CH_3_), 68.5(9), 68.5(6) (Xyl’-C-4 & Fuc-C-3), 67.7 (Glc-C-6), 64.9 (Fuc-C-5), 64.0 (Xyl-C-5), 62.4 (Xyl’-C-5), 55.2 (ArOCH_3_), 31.8 (OCH_2_(CH_2_)_6_CH_3_), 29.6 (OCH_2_(CH_2_)_6_CH_3_), 29.4 (OCH_2_(CH_2_)_6_CH_3_), 29.2 (OCH_2_(CH_2_)_6_CH_3_), 26.1 (OCH_2_(CH_2_)_6_CH_3_), 22.6 (OCH_2_(CH_2_)_6_CH_3_), 20.8 (COCH_3_), 20.7 (COCH_3_), 20.6(5) (COCH_3_), 16.2 (Fuc-C-6), 14.1 (O(CH_2_)_7_CH_3_); HRMS (ESI) Calc. for [M + Na]^+^ C_79_H_98_NaO_22_: 1421.6442; Found 1421.6439.

**Octyl 2,3,4-tri-*O*-acetyl-β-D-xylopyranosyl-(1→4)-[2,3,4-tri-*O*-acetyl-α-D-rhamnopyranosyl-(1→3)]-α-L-fucopyranosyl-(1→3)-[2,3,4-tri-*O*-benzyl-β-D-xylopyranosyl-(1→4)]-2,6-di-*O*-benzyl-β-D-glucopyranoside (S32):** To a stirred solution of acceptor **S31** (66.4 mg, 47.4 µmol) and donor **S23** (37.6 mg, 94.8 µmol) in dry CH_2_Cl_2_ (3.0 mL) was added oven-dried molecular sieves (0.3 g, 4Å, powder) under an Ar atmosphere. After stirring at room temperature for 30 min, the solution was chilled to –30 ˚C and *N*-iodosuccinimide (21.4 mg, 95.1 µmol) and trifluoromethanesulfonic acid (1.24 µL, 14.1 µmol) were added successively. The resulting solution was stirred at –30 ˚C before for 50 min before triethylamine was added. The solution was filtered through Celite and the filtrate was concentrated. The crude residue was passed through a silica column (3:2 hexanes–EtOAc). The resulting product was dissolved in dry CH_2_Cl_2_ (5.0 mL) and then trifluoroacetic acid (0.05 mL) was added drop-wise at 0 ˚C, under an Ar atmosphere. The reaction mixture was slowly warmed to room temperature and stirred for 5 h and was then chilled to 0 ˚C and triethylamine added. The solution was concentrated and the resulting residue was purified via flash chromatography (2:1 hexanes–EtOAc→3:2 hexanes–EtOAc) to give **S32** (52.1 mg, 71%) as a colourless film. *R*_f_ 0.44 (3:2 EtOAc–hexanes); [α]ᴅ –40.25 (*c* 2.2, CHCl_3_); ^1^H NMR (700 MHz; CDCl_3_): δ 7.36–7.22 (m, 25H, Ar), 5.46 (d, 1H, *J* = 4.0 Hz, Fuc-H-1), 5.44 (dd, 1H, *J* = 3.5, 1.8 Hz, Rha-H-2), 5.38 (dd, 1H, *J* = 10.1, 3.4 Hz, Rha-H-3), 5.15 (d, 1H, *J* = 1.8 Hz, Rha-H-1), 5.08 (app t, 1H, *J* = 6.3 Hz, Xyl’-H-3), 5.05 (app t, 1H, *J* = 9.9 Hz, Rha-H-4), 4.96 (dd, 1H, *J* = 6.4, 4.6 Hz, Xyl’-H-2), 4.92–4.87 (m, 2H, ArCH_2_, Xyl’-H-4), 4.82‒4.75 (m, 3H, ArCH_2_), 4.70 (d, 1H, *J* = 11.3 Hz, ArCH_2_), 4.68–4.54 (m, 6H, ArCH_2_, Xyl’-H-1, Fuc-H-5), 4.46 (dd, 1H, *J* = 12.4, 3.9 Hz, Xyl’-H-5a), 4.42 (d, 1H, *J* = 12.0 Hz, ArCH_2_), 4.37 (d, 1H, *J* = 8.0 Hz, Xyl-H-1), 4.33 (d, 1H, *J* = 7.7 Hz, Glc-H-1), 4.17 (dq, 1H, *J* = 9.8, 6.3 Hz, Rha-H-5), 3.99‒3.94 (m, 2H, Fuc-H-3, Xyl-H-5a), 3.93–3.86 (m, 3H, OCH_2_(CH_2_)_6_CH_3_, Fuc-H-2, Glc-H-6a), 3.85 (app t, 1H, *J* = 9.5 Hz, Glc-H-4), 3.81 (app t, 1H, *J* = 9.3 Hz, Glc-H-3), 3.65–3.59 (m, 2H, Glc-H-6b, Xyl’-H-5b), 3.54 (app d, 1H, *J* = 2.9 Hz, Fuc-H-4), 3.52–3.44 (m, 2H, OCH_2_(CH_2_)_6_CH_3_, Xyl-H-4), 3.41‒3.37 (Glc-H-2, Xyl-H-3), 3.28 (ddd, 1H, *J* = 9.5, 3.3, 1.8 Hz, Glc-H-5), 3.07 (dd, 1H, *J* = 9.1, 8.1 Hz, Xyl-H-2), 3.00 (app t, 1H, *J* = 11.1 Hz, Xyl-H-5b), 2.13 (s, 3H, COCH_3_), 2.07 (s, 3H, COCH_3_), 2.05 (s, 3H, COCH_3_), 2.03 (s, 3H, COCH_3_), 1.97 (s, 3H, COCH_3_), 1.97 (s, 3H, COCH_3_), 1.68–1.56 (m, 2H, OCH_2_CH_2_(CH_2_)_5_CH_3_), 1.41–1.20 (m, 10H, OCH_2_CH_2_(CH_2_)_5_CH_3_), 1.17 (d, 3H, *J* = 6.3 Hz, Rha-H-6), 1.14 (d, 3H, *J* = 6.7 Hz, Fuc-H-6), 0.86 (t, 3H, *J* = 7.0 Hz, O(CH_2_)_7_CH_3_); ^13^C NMR (176 MHz; CDCl_3_): δ 170.1 (COCH_3_), 169.9 (COCH_3_ × 2), 169.8 (COCH_3_), 169.7 (COCH_3_), 169.3 (COCH_3_), 138.3 (Ar), 138.2 (Ar), 138.1 (Ar), 137.9 (Ar), 128.4(5) (Ar), 128.4(3) (Ar), 128.4(1) (Ar), 128.3(8) (Ar), 128.3(7) (Ar), 128.3(4) (Ar), 128.0 (Ar), 127.9 (Ar), 127.7(7) (Ar), 127.7(6) (Ar), 127.7(3) (Ar), 127.7 (Ar), 127.6 (Ar), 127.5(5) (Ar), 127.3 (Ar), 103.5 (Glc-C-1), 103.2 (Xyl-C-1), 100.1 (Xyl’-C-1), 99.4 (Rha-C-1, *J*_C-H_ = 175.5 Hz), 97.8 (Fuc-C-1), 84.1 (Xyl-C-3), 82.7 (Glc-C-2), 82.4 (Xyl-C-2), 81.1 (Fuc-C-4), 78.5 (Xyl-C-4), 76.9 (Fuc-C-3), 75.8 (ArCH_2_), 75.1 (ArCH_2_), 75.0(7), 74.9(6) (Glc-C-5 & Glc-C-3), 74.7 (Glc-C-4), 74.6 (ArCH_2_), 73.4 (ArCH_2_), 73.2 (ArCH_2_), 71.3 (Rha-C-4), 70.2 (OCH_2_(CH_2_)_6_CH_3_), 70.0 (Rha-C-2), 69.4 (Xyl’-C-2), 69.2 (Xyl’-C-3), 69.0 (Rha-C-3), 68.7 (Fuc-C-2), 68.4 (Xyl’-C-4), 67.6 (Glc-C-6), 66.4 (Rha-C-5), 66.0 (Fuc-C-5), 64.0 (Xyl-C-5), 60.5 (Xyl’-C-5), 31.8 (OCH_2_(CH_2_)_6_CH_3_), 29.7 (OCH_2_(CH_2_)_6_CH_3_), 29.4 (OCH_2_(CH_2_)_6_CH_3_), 29.2 (OCH_2_(CH_2_)_6_CH_3_), 26.2 (OCH_2_(CH_2_)_6_CH_3_), 22.6 (OCH_2_(CH_2_)_6_CH_3_), 20.9 (COCH_3_), 20.8(8) (COCH_3_), 20.8(2) (COCH_3_), 20.8(1) (COCH_3_), 20.7 (COCH_3_), 20.6(9) (COCH_3_), 17.9 (Rha-C-6), 16.4 (Fuc-C-6), 14.1 (O(CH_2_)_7_CH_3_); HRMS (ESI) Calc. for [M + Na]^+^ C_83_H_106_Na_2_O_28_: 798.3328; Found 798.3334.

**Octyl 2,3,4-tri-*O*-acetyl-β-D-xylopyranosyl-(1→4)-[2,3,4-tri-*O*-acetyl-α-D-rhamnopyranosyl-(1→3)]-[ 2,3-di-*O*-benzyl-4,6-*O*-di-*tert*-butylsilylene-α-D-galactopyranosyl-(1→2)]-α-L-fucopyranosyl-(1→3)-[2,3,4-tri-*O*-benzyl-β-D-xylopyranosyl-(1→4)]-2,6-di-*O*-benzyl-β-D-glucopyranoside (S33):**

To a stirred solution of acceptor **S32** (49.8 mg, 32.1 µmol) and donor **S4**^11^ (38.5 mg, 63.4 µmol) in dry Et_2_O (4.0 mL) was added oven-dried molecular sieves (0.4 g, 4Å, powder) under an Ar atmosphere. After stirring at room temperature for 30 min, methyl trifluoromethanesulfonate (0.03 mL, 265 µmol) was added drop-wise at room temperature. The reaction mixture was stirred at room temperature for 26 h before triethylamine was added. The solution was filtered through Celite and the filtrate was concentrated. The crude residue was purified via flash chromatography (2:1:1 hexanes–EtOAc–CH_2_Cl_2_) to give **S33** (44.7 mg, 69%) as a colourless film. *R*_f_ 0.56 (3:1 hexanes–EtOAc); [α]ᴅ –20.1 (*c* 3.7, CHCl_3_); ^1^H NMR (700 MHz; CDCl_3_): δ 7.40–7.18 (m, 33H, Ar), 7.12–7.08 (m, 2H, Ar), 5.44 (dd, 1H, *J* = 3.2, 1.8 Hz, Rha-H-2), 5.38‒5.36 (m, 2H, Fuc-H-1, Rha-H-3), 5.13 (app t, 1H, *J* = 6.7 Hz, Xyl’-H-3), 5.10–5.08 (m, 2H, Xyl’-H-2, Rha-H-1), 4.99–4.92 (m, 4H, Rha-H-4, Gal-H-1, Xyl’-H-4, ArCH_2_), 4.88 (d, 1H, *J* = 12.3 Hz, ArCH_2_), 4.82 (ABq, 2H, *J*_AB_ = 10.9 Hz, ArCH_2_), 4.78 (d, 1H, *J* = 11.2 Hz, ArCH_2_), 4.71–4.68 (m, 2H, ArCH_2_, Xyl’-H-1), 4.67 (d, 1H, *J* = 11.2 Hz, ArCH_2_), 4.64 (d, 1H, *J* = 12.2 Hz, ArCH_2_), 4.62 (d, 1H, *J* = 12.4 Hz, ArCH_2_), 4.61–4.57 (m, 3H, ArCH_2_), 4.55 (app q, 1H, *J* = 6.8 Hz, Fuc-H-5), 4.47 (d, 1H, *J* = 12.0 Hz, ArCH_2_), 4.43 (d, 1H, *J* = 8.0 Hz, Xyl-H-1), 4.42–4.38 (m, 2H, Xyl’-H-5a, Fuc-H-3), 4.37 (d, 1H, *J* = 7.4 Hz, Glc-H-1), 4.34‒4.26 (m, 2H, ArCH_2_, Rha-H-5), 4.21 (app d, 1H, *J* = 2.9 Hz, Gal-H-4), 4.08 (dd, 1H, *J* = 11.6, 5.7 Hz, Xyl-H-5a), 4.03 (dd, 1H, *J* = 10.2, 3.6 Hz, Fuc-H-2), 3.98 (dd, 1H, *J* = 10.0, 3.6 Hz, Gal-H-2), 3.96 (app t, 1H, *J* = 9.2 Hz, Glc-4), 3.93‒3.83 (m, 3H, Glc-H-3, Glc-H-6a, OCH_2_(CH_2_)_6_CH_3_), 3.81 (dd, 1H, *J* = 10.1, 2.9 Hz, Gal-H-3), 3.73 (dd, 1H, *J* = 13.0, 2.0 Hz, Gal-H-6a), 3.69 (m, 1H, Xyl-H-4), 3.65 (dd, 1H, *J* = 10.9, 2.1 Hz, Glc-H-6b), 3.62 (app d, 1H, *J* = 3.0 Hz, Fuc-H-4), 3.57 (dd, 1H, *J* = 12.1, 6.4 Hz, Xyl’-H-5b), 3.44 (app t, 1H, *J* = 8.9 Hz, Xyl-H-3), 3.40–3.31 (m, 5H, OCH_2_(CH_2_)_6_CH_3_, Glc-H-5, Glc-H-2, Gal-H-6b, Gal-H-5), 3.12‒3.08 (m, 2H, Xyl-H-5b, Xyl-H-2), 2.08 (s, 3H, COCH_3_), 2.06 (s, 3H, COCH_3_), 2.01 (s, 3H, COCH_3_), 2.01 (s, 3H, COCH_3_), 1.88 (s, 3H, COCH_3_), 1.67 (s, 3H, COCH_3_), 1.58–1.51 (m, 2H, OCH_2_CH_2_(CH_2_)_5_CH_3_), 1.21 (d, 3H, *J* = 6.8 Hz, Fuc-H-6), 1.28–1.16 (m, 10H, OCH_2_CH_2_(CH_2_)_5_CH_3_), 1.16 (d, 3H, *J* = 6.4 Hz, Rha-H-6), 0.97 (s, 9H, C(CH_3_)_3_), 0.94 (s, 9H, C(CH_3_)_3_), 0.84 (t, 3H, *J* = 7.3 Hz, O(CH_2_)_7_CH_3_); ^13^C NMR (176 MHz; CDCl_3_): δ 170.1 (COCH_3_), 170.0(8) (COCH_3_), 169.7 (COCH_3_), 169.6 (COCH_3_), 169.4 (COCH_3_), 169.1 (COCH_3_), 139.6 (Ar), 139.1 (Ar), 138.6 (Ar), 138.5(5) (Ar), 138.4 (Ar), 138.3 (Ar), 138.1 (Ar), 128.7 (Ar), 128.5 (Ar), 128.4(6) (Ar), 128.4(4) (Ar), 128.4(2) (Ar), 128.3(8) (Ar), 128.3(5) (Ar), 128.3(4), 128.3(3) (Ar), 128.2(2) (Ar), 128.2(1) (Ar), 128.1(5) (Ar), 128.0 (Ar), 127.9 (Ar), 127.8(9) (Ar), 127.8(5) (Ar), 127.7(3) (Ar), 127.7 (Ar), 127.6(5) (Ar), 127.6(2) (Ar), 127.6 (Ar), 127.5(7) (Ar), 127.5 (Ar), 127.3(4) (Ar), 127.3 (Ar), 127.2 (Ar), 127.0 (Ar), 126.9 (Ar), 125.8 (Ar), 103.3 (Xyl-C-1), 103.1 (Glc-C-1), 100.3 (Rha-C-1), 100.2 (Xyl’-C-1), 99.1 (Gal-C-1, *J*_C-H_ = 172.6 Hz), 95.9 (Fuc-C-1), 84.0 (Xyl-C-3), 82.5 (Xyl-C-2), 82.1 (Glc-C-2), 81.2 (Fuc-C-4), 78.6 (Xyl-C-4), 77.3 (Gal-C-3), 76.7 (Fuc-C-3), 75.7 (ArCH_2_), 75.1 (ArCH_2_), 75.0, 74.9 (Glc-C-5 & Glc-C-4), 73.7 (Gal-C-2), 73.4 (ArCH_2_), 73.3(6) (Glc-C-3), 73.0 (ArCH_2_), 72.9 (Fuc-C-2), 72.4 (ArCH_2_), 72.2 (ArCH_2_), 71.4 (Rha-C-4), 71.2 (Gal-C-4), 70.7 (ArCH_2_), 69.9, 69.8 (Xyl’-C-2 & Rha-C-2), 69.7(5) (OCH_2_(CH_2_)_6_CH_3_), 69.6 (Xyl’-C-3), 69.4 (Rha-C-3), 68.6 (Xyl’-C-4), 67.8 (Glc-C-6), 67.6 (Gal-C-5), 66.6 (Gal-C-6), 66.4 (Rha-C-5), 65.6 (Fuc-C-5), 64.0 (Xyl-C-5), 60.6 (Xyl’-C-5), 31.8 (OCH_2_(CH_2_)_6_CH_3_), 29.7 (OCH_2_(CH_2_)_6_CH_3_), 29.4 (OCH_2_(CH_2_)_6_CH_3_), 29.2 (OCH_2_(CH_2_)_6_CH_3_), 28.0 (C(CH_3_)_3_), 27.7 (C(CH_3_)_3_), 27.6 (C(CH_3_)_3_), 27.3 (C(CH_3_)_3_), 26.1 (OCH_2_(CH_2_)_6_CH_3_), 23.2 (C(CH_3_)_3_), 22.6 (OCH_2_(CH_2_)_6_CH_3_), 20.8(4) (COCH_3_), 20.8(3) (COCH_3_ × 2), 20.8 (COCH_3_), 20.6(4) (COCH_3_), 20.6 (C(CH_3_)_3_), 20.5 (COCH_3_), 18.1 (Rha-C-6), 16.6 (Fuc-C-6), 14.1 (O(CH_2_)_7_CH_3_); HRMS (ESI) Calc. for [M + Na]^+^ C_111_H_144_Na_2_O_33_Si: 1039.4572; Found 1039.4621.

**Octyl 2,3,4-tri-*O*-acetyl-β-D-xylopyranosyl-(1→4)-[2,3,4-tri-*O*-acetyl-α-D-rhamnopyranosyl-(1→3)]-[2,3-di-*O*-benzyl-α-D-galactopyranosyl-(1→2)]-α-L-fucopyranosyl-(1→3)-[2,3,4-tri-*O*-benzyl-β-D-xylopyranosyl-(1→4)]-2,6-di-*O*-benzyl-β-D-glucopyranoside (S34):** To a stirred solution of **S33** (62.6 mg, 30.8 µmol) in THF–pyridine (3.75:1, 3.75 mL THF, 1.00 mL pyridine) under an Ar atmosphere was added HF∙pyridine (0.20 mL, hydrogen fluoride ∼70%, pyridine ∼30%) at 0 ˚C. The reaction mixture was slowly warmed to room temperature and stirred for a total of 24.5 h. The reaction mixture was diluted with EtOAc (15 mL) and poured into a saturated solution of NaHCO_3_ (aq). The aqueous layer was extracted with EtOAc (10 mL × 3) and the combined organic layers were dried over Na_2_SO_4_, filtered and then the filtrate was concentrated. The crude residue was purified via flash chromatography (1:1 EtOAc–hexanes→19:1 EtOAc–CH_3_OH) to give **S34** (42.5 mg, 73%) as a colourless film. Due to time constraints, the compound was not fully characterized (missing mass spectrum and optical rotation). *R*_f_ 0.26 (1:2:1 EtOAc–hexanes–acetone); ^1^H NMR (700 MHz; CDCl_3_): δ 7.37–7.16 (m, 40H, Ar), 5.48 (d, 1H, *J* = 3.5 Hz, Fuc-H-1), 5.38 (dd, 1H, *J* = 3.2, 1.7 Hz, Rha-H-2), 5.35 (dd, 1H, *J* = 10.0, 3.2 Hz, Rha-H-3), 5.23‒5.19 (m, 2H, Xyl’-H-3, Gal-H-1), 5.13 (dd, 1H, *J* = 8.6, 6.3 Hz, Xyl’-H-2), 5.02 (d, 1H, *J* = 1.8 Hz, Rha-H-1), 4.98‒4.93 (m, 2H, Rha-H-4, Xyl’-H-4), 4.89 (d, 1H, *J* = 12.0 Hz, ArCH_2_), 4.85‒4.77 (m, 4H, ArCH­_2_), 4.69 (d, 1H, *J* = 11.3 Hz, ArCH_2_), 4.68 (d, 1H, *J* = 11.2 Hz, ArCH_2_), 4.66‒4.62 (m, 3H, ArCH­­_2_­, Xyl’-H-1), 4.62–4.56 (m, 3H, ArCH_2_, Fuc-H-5), 4.53 (d, 1H, *J* = 11.7 Hz, ArCH_2_), 4.47 (d, 1H, *J* = 12.0 Hz, ArCH_2_), 4.46 (d, 1H, *J* = 8.0 Hz, Xyl-H-1), 4.43 (dd, 1H, *J* = 10.3, 2.7 Hz, Fuc-H-3), 4.38 (d, 1H, *J* = 7.5 Hz, Glc-H-1), 4.38‒4.34 (m, 2H, ArCH_2_, Rha-H-5), 4.30 (dd, 1H, *J* = 11.8, 4.9 Hz, Xyl’-H-5a), 4.17 (dd, 1H, *J* = 10.3, 3.5 Hz, Fuc-H-2), 4.08 (dd, 1H, *J* = 11.5, 5.6 Hz, Xyl-H-5a), 4.01 (app t, 1H, *J* = 9.1 Hz, Glc-H-4), 3.95 (app t, 1H, *J* = 8.5 Hz, Glc-H-3), 3.90 (dd, 1H, *J* = 10.8, 3.4 Hz, Glc-H-6a), 3.87 (dt, 1H, *J* = 9.6, 6.5 Hz, OCH_2_(CH_2_)_6_CH_3_), 3.84 (dd, 1H, *J* = 9.7, 3.3 Hz, Gal-H-2), 3.81 (dd, 1H, *J* = 9.8, 3.0 Hz, Gal-H-3), 3.70–3.62 (m, 4H, Fuc-H-4, Xyl-H-4, Glc-H-6b, Gal-H-4), 3.50‒3.47 (m, 2H, Gal-H-6a, Gal-H-5), 3.47–3.43 (m, 2H, Glc-H-2, Xyl-H-3), 3.42–3.35 (m, 3H, Xyl’-H-5b, OCH_2_(CH_2_)_6_CH_3_, Glc-H-5), 3.17–3.08 (m, 3H, Gal-H-6b, Xyl-H-5b, Xyl-H-2), 2.09 (s, 3H, COCH_3_), 2.03 (s, 3H, COCH_3_), 2.02 (s, 3H, COCH_3_), 2.02 (s, 3H, COCH_3_), 1.89 (s, 3H, COCH_3_), 1.65 (s, 3H, COCH_3_), 1.59–1.52 (m, 2H, OCH_2_CH_2_(CH_2_)_5_CH_3_), 1.22 (d, 3H, *J* = 6.8 Hz, Fuc-H-6), 1.31–1.15 (m, 10H, OCH_2_CH_2_(CH_2_)_5_CH_3_), 1.14 (d, 3H, *J* = 6.3 Hz, Rha-H-6), 0.84 (t, 3H, *J* = 7.2 Hz, O(CH_2_)_7_CH_3_); ^13^C NMR (176 MHz; CDCl_3_): δ 170.2 (COCH_3_), 170.1 (COCH_3_), 170.0 (COCH_3_), 169.7 (COCH_3_), 169.6 (COCH_3_), 169.4 (COCH_3_), 139.4 (Ar), 138.5 (Ar), 138.3 (Ar), 138.2(7) (Ar), 138.0(7) (Ar), 138.0 (Ar), 128.5 (Ar), 128.4(7) (Ar), 128.4 (Ar), 128.3(6) (Ar), 128.3(5) (Ar), 128.2 (Ar), 128.0(2) (Ar), 128.0 (Ar), 127.9(8) (Ar), 127.9 (Ar), 127.7(4) (Ar), 127.7 (Ar), 127.6(9) (Ar), 127.6(5) (Ar), 127.6 (Ar), 127.3 (Ar), 127.0 (Ar), 126.9 (Ar), 126.2 (Ar), 103.3 (Xyl-C-1), 103.2 (Glc-C-1), 100.7 (Xyl’-C-1), 100.2 (Rha-C-1), 97.8 (Gal-C-1), 96.3 (Fuc-H-1), 84.0 (Xyl-C-3), 82.5 (Xyl-C-2), 82.0 (Glc-C-2), 80.1 (Fuc-C-4), 78.6 (Xyl-C-4), 77.1 (Gal-C-3), 76.9 (Fuc-C-3), 75.8 (ArCH_2_), 75.1 (ArCH_2_), 75.0(8) × 2, 74.9(8) (Glc-C-4 & Gal-C-2 & Glc-C-5), 73.6 (Glc-C-3), 73.4 (ArCH_2_), 73.0 (ArCH_2_), 72.6 (ArCH_2_), 72.3 (ArCH_2_), 72.2 (ArCH_2_), 71.3, 71.2 (Rha-C-4 & Fuc-C-2), 70.9 (Xyl’-C-2), 70.5 (Xyl’-C-3, Gal-C-5), 69.9 (OCH_2_(CH_2_)_6_CH_3_), 69.8 (Rha-C-2), 69.4, 69.3 (Xyl’-C-4 & Rha-C-3), 68.7 (Gal-C-4), 67.8 (Glc-C-6), 66.4 (Rha-C-5), 65.6 (Fuc-C-5), 64.0 (Xyl-C-5), 62.3 (Gal-C-6), 61.5 (Xyl’-C-5), 31.8 (OCH_2_(CH_2_)_6_CH_3_), 29.7 (OCH_2_(CH_2_)_6_CH_3_), 29.4 (OCH_2_(CH_2_)_6_CH_3_), 29.2 (OCH_2_(CH_2_)_6_CH_3_), 26.1 (OCH_2_(CH_2_)_6_CH_3_), 22.6 (OCH_2_(CH_2_)_6_CH_3_), 20.8 (COCH_3_), 20.7(6) (COCH_3_), 20.7(5) (COCH_3_), 20.7 (COCH_3_), 20.6 (COCH_3_), 20.4 (COCH_3_), 18.0 (Rha-C-6), 16.9 (Fuc-C-6), 14.1 (O(CH_2_)_7_CH_3_).

**Octyl β-D-xylopyranosyl-(1→4)-[α-D-rhamnopyranosyl-(1→3)]-[α-D-galactopyranosyl-(1→2)]-α-L-fucopyranosyl-(1→3)-[β-D-xylopyranosyl-(1→4)]-β-D-glucopyranoside (9):** To a stirred solution of **S34** (42.4 mg, 22.4 µmol) in CH_3_OH (6.0 mL) was added NaOH (56.6 mg, 1.42 mmol). The reaction mixture was stirred at room temperature for 17.5 h. Amberlite® IR-120 (H^+^) cation exchange resin was added, the mixture filtered and then the filtrate was concentrated. The crude residue was purified via flash chromatography (7:1 EtOAc–CH_3_OH). The residue was dissolved in THF–H_2_O (1:1, 2.0 mL THF, 2.0 mL H_2_O) and 20% Pd(OH)_2_/C (12.2 mg) was added under an Ar atmosphere. The reaction mixture was stirred at room temperature, under a H_2_ atmosphere, for 38 h and was then filtered and the filtrate concentrated. The residue was dissolved in pyridine (2.0 mL) and acetic anhydride (1.0 mL) was added at 0 ˚C. The reaction mixture was warmed to room temperature and stirred for 20 h and then concentrated. The crude residue was passed through a silica column (3:2 hexanes–EtOAc). The crude product was dissolved in CH_3_OH (6.0 mL) and NaOH (56.4 mg, 1.41 mmol) was added. The reaction mixture was stirred at room temperature for 18 h. Amberlite® IR-120 (H^+^) cation exchange resin was added, the mixture filtered and then the filtrate was concentrated. The crude product was purified via Iatrobead chromatography (CH_2_Cl_2_–CH_3_OH→CH_3_OH, 10% stepwise gradient) to give **9** as a white film (tentative 13.4 mg, 59%). *R*_f_ 0.26 (4:1:1:1 EtOAc–CH_3_OH–H_2_O–AcOH); [α]ᴅ –19.4 (*c* 0.5, CHCl_3_); ^1^H NMR (700 MHz; D_2_O): δ 5.64 (d, 1H *J* = 3.9 Hz, Fuc-H-1), 5.23 (d, 1H, *J* = 4.0 Hz, Gal-H-1), 5.05 (app s, 1H, Rha-H-1), 4.78–4.74 (m, 1H, Fuc-H-5), 4.47 (d, 1H, *J* = 7.7 Hz, Xyl’-H-1), 4.45 (d, 1H, *J* = 8.0 Hz, Glc-H-1), 4.42 (d, 1H, *J* = 7.9 Hz, Xyl-H-1), 4.22 (dd, 1H, *J* = 10.6, 2.8 Hz, Fuc-H-3), 4.15 (dd, 1H, *J* = 10.6, 3.9 Hz, Fuc-H-2), 4.11 (dt, 1H, *J* = 12.8, 6.3 Hz, Rha-H-5), 4.08–3.97 (m, 6H, Gal-H-5, Rha-H-2, Xyl’-H-5a, Gal-H-4, Xyl-H-5a, Glc-H-6a), 3.93–3.79 (m, 6H, OCH_2_(CH_2_)_6_CH_3_, Glc-H-3, Fuc-H-4, Gal-H-3, Glc-H-6b, Gal-H-2), 3.78–3.64 (m, 6H, Rha-H-3, Gal-H-6a, Gal-H-6b, OCH_2_(CH_2_)_6_CH_3_, Glc-H-4, Xyl’-H-4), 3.63–3.55 (m, 2H, Xyl-H-4, Glc-H-5), 3.50 (app t, 1H, *J* = 9.6 Hz, Rha-H-4), 3.48–3.43 (m, 3H, Xyl-H-3, Xyl’-H-3, Glc-H-2), 3.39 (app t, 1H, *J* = 8.6 Hz, Xyl’-H-2), 3.28 (app t, 1H, *J* = 11.1 Hz, Xyl-H-5b), 3.24 (app t, 1H, *J* = 11.2 Hz, Xyl’-H-5b), 3.15 (app t, 1H, *J* = 8.6 Hz, Xyl-H-2), 1.63 (app pent, *J* = 7.0 Hz, 2H, OCH_2_CH_2_(CH_2_)_5_CH_3_), 1.39–1.25 (m, 16H, OCH_2_CH_2_(CH_2_)_5_CH_3_, Fuc-H-6, Rha-H-6), 0.87 (t, 3H, *J* = 6.7 Hz, O(CH_2_)_7_CH_3_); ^13^C NMR (176 MHz; D_2_O): δ 105.1 (Xyl’C-1), 103.7, 103.6 (Rha-C-1 & Xyl-C-1), 103.2 (Glc-C-1), 99.8 (Gal-C-1), 98.1 (Fuc-C-1), 82.0 (Fuc-C-4), 76.7, 76.6, 76.5 (Fuc-C-3 & Xyl-C-3 & Xyl’-C-3), 76.0, 75.9, 75.8 (Glc-C-2 & Glc-C-3 & Glc-C-5), 74.9 (Glc-C-4), 74.5, 74.4 (Xyl-C-2 & Xyl’C-2), 72.8 (Rha-C-4), 72.1 (Gal-C-5), 71.8 (OCH_2_(CH_2_)_6_CH_3_), 71.6 (Fuc-C-2), 71.1, 71.0 (Rha-C-2 & Rha-C-3), 70.6, 70.5 (Xyl-C-4 & Gal-C-3), 70.2 (Xyl’-C-4), 70.0 (Gal-C-4), 69.7 (Rha-C-5), 69.2 (Gal-C-2), 68.0 (Fuc-C-5), 65.7 × 2 (Xyl-C-5, Xyl’-C-5), 61.9 (Gal-C-6), 60.4 (Glc-C-6), 31.9 (OCH_2_(CH_2_)_6_CH_3_), 29.6 (OCH_2_(CH_2_)_6_CH_3_), 29.3 (OCH_2_(CH_2_)_6_CH_3_), 29.2 (OCH_2_(CH_2_)_6_CH_3_), 25.9 (OCH_2_(CH_2_)_6_CH_3_), 22.9 (OCH_2_(CH_2_)_6_CH_3_), 17.5 (Rha-C-6), 15.7 (Fuc-C-6), 14.2 (O(CH_2_)_7_CH_3_).

***Synthesis of 10***

**Octyl 4-*O*-benzyl-2,3-*O*-isopropylidene-α-D-rhamnopyranosyl-(1→3)-4-*O*-acetyl-2-*O-tert-*butyldimethylsilyl-α-L-fucopyranoside (S35):** To a stirred solution of **S1**^1^ (161 mg, 272 μmol) in dry CH_2_Cl_2_ (5.0 mL) was added *tert*-butyldimethylsilyl trifluoromethanesulfonate (93.0 μL, 406 μmol) and 2,6-lutidine (63.0 μL, 541 μmol) at 0 °C. The reaction mixture was stirred for 2 h at room temperature. The crude residue was diluted with CH_2_Cl_2_ (50 mL), then washed with 1N HCl and saturated NaHCO_3_ (aq.). The organic layer was dried over Na_2_SO_4_, filtered and the filtrate was concentrated. The crude residue was purified by flash chromatography (8:1 hexane–EtOAc) to afford **S35** (186 mg, 97%) as a viscous oil. *R*_f_ 0.63 (4:1 hexane–EtOAc); [α]_D_ –40.7 (*c* 0.45, CHCl_3_); ^1^H NMR (700 MHz; CDCl_3_): *δ* 7.35–7.30 (m, 4H, Ar), 7.26–7.24 (m, 1H, Ar), 5.17 (s, 1H, Rha-H-1), 5.15 (dd, 1H, *J* = 3.6, 1.2 Hz, Fuc-H-4), 4.86 (d, 1H, *J* = 11.8 Hz, PhCH_2_), 4.71 (d, 1H, *J* = 3.7 Hz, Fuc-H-1), 4.63 (d, 1H, *J* = 11.8 Hz, PhCH_2_), 4.15–4.12 (m, 2H, Rha-H-2, Rha-H-3), 4.07–4.04 (m, 2H, Fuc-H-3, Fuc-H-5), 3.94 (dd, 1H, *J* = 9.9, 3.7 Hz, Fuc-H-2), 3.73 (dq, 1H, *J* = 9.8, 6.2 Hz, Rha-H-5), 3.63 (dt, 1H, *J* = 9.7, 6.6 Hz, OCH_2_CH_2_(CH_2_)_5_CH_3_), 3.36 (dt, 1H, *J* = 9.7, 6.7 Hz, OCH_2_CH_2_(CH_2_)_5_CH_3_), 3.15 (dd, 1H, *J* = 9.8, 6.6 Hz, Rha-H-4), 2.13 (s, 3H, COCH_3_), 1.58 (quintet, 2H, *J* = 7.2 Hz, OCH_2_CH_2_(CH_2_)_5_CH_3_), 1.45 (s, 3H, C(CH_3_)_2_), 1.36–1.25 (m, 16H, C(CH_3_)_2_, OCH_2_CH_2_(CH_2_)_5_CH_3_, Rha-H-6), 1.07 (d, 3H, *J* = 6.6 Hz, Fuc-H-6), 0.88–0.86 (m, 12H, SiC(CH_3_)_3_, OCH_2_CH_2_(CH_2_)_5_CH_3_), 0.07 (s, 3H, SiCH_3_), 0.07 (s, 3H, SiCH_3_); ^13^C NMR (125 MHz; CDCl_3_): *δ* 170.5 (C=O), 138.6 (Ar), 128.2 (Ar), 127.9 (Ar), 127.5 (Ar), 108.7 (C(CH_3_)_2_), 99.5 (2 × C, Fuc-H-1, Rha-H-1), 80.9 (Rha-C-4), 78.5 (Rha-C-3), 76.1 (Rha-C-2), 75.0 (Fuc-C-3), 73.8 (Fuc-C-4), 72.9 (PhCH_2_), 70.1 (Fuc-C-2), 68.6 (OCH_2_(CH_2_)_6_CH_3_), 65.1 (Rha-C-5), 64.9 (Fuc-C-5), 31.9 (OCH_2_(CH_2_)_6_CH_3_), 29.6 (OCH_2_(CH_2_)_6_CH_3_), 29.4 (OCH_2_(CH_2_)_6_CH_3_), 29.3 (OCH_2_(CH_2_)_6_CH_3_), 28.0 (C(CH_3_)_2_), 26.3 (OCH_2_(CH_2_)_6_CH_3_), 26.2 (C(CH_3_)_2_), 25.8 (SiC(CH_3_)_3_), 22.7 (OCH_2_(CH_2_)_6_CH_3_), 20.9 (COCH_3_), 18.0 (SiC(CH_3_)_3_), 17.6 (Rha-C-6), 16.2 (Fuc-C-6), 14.1 (OCH_2_(CH_2_)_6_CH_3_), –4.4 (SiCH_3_), –4.8 (SiCH_3_); HRMS (ESI) Calc. for [M + NH_4_]^+^ C_38_H_68_NO_10_Si: 726.4607; Found 726.4604.

**Octyl 4-*O*-benzyl-2,3-*O*-isopropylidene-α-D-rhamnopyranosyl-(1→3)-2-*O-tert-*butyldimethylsilyl-α-L-fucopyranoside (S36):** To a stirred solution of **S35** (182 mg, 257 μmol) in CH_3_OH (6.0 mL) was added a solution of NaOCH_3_ in CH_3_OH (0.6 mL, 0.5 M). The reaction mixture was stirred for 6 h at room temperature, then neutralized by addition of Amberlite® IR-120 (H^+^) cation exchange resin, filtered and the filtrate was concentrated to afford **S36** (168 mg, 98%) as a viscous oil. *R*_f_ 0.40 (4:1 hexane–EtOAc); [α]_D_ –30.8 (*c* 0.31, CHCl_3_); ^1^H NMR (600 MHz; CDCl_3_): *δ* 7.38–7.34 (m, 4H), 7.31–7.28 (m, 1H), 5.28 (s, 1H, Rha-C-1), 4.92 (d, 1H, *J* = 11.6 Hz, PhCH_2_), 4.71 (d, 1H, *J* = 2.5 Hz, Fuc-H-1), 4.64 (d, 1H, *J* = 11.6 Hz, PhCH_2_), 4.30–4.25 (m,2H, Rha-H-3, Rha-H-2), 4.02–3.97 (m, 3H, Fuc-H-5, Fuc-H-2, Fuc-H-3), 3.79–3.74 (m, 2H, Rha-H-5, Fuc-H-4), 3.67 (dt, 1H, *J* = 9.7, 6.6 Hz, OCH_2_CH_2_(CH_2_)_5_CH_3_), 3.40 (dt, 1H, *J* = 9.7, 6.7 Hz, OCH_2_CH_2_(CH_2_)_5_CH_3_), 3.24 (dd, 1H, *J* = 9.7, 7.0 Hz, Rha-H-4), 2.15 (br s, 1H, 4-OH), 1.61 (quintet, 2H, *J* = 7.1 Hz, OCH_2_CH_2_(CH_2_)_5_CH_3_), 1.51 (s, 3H, C(CH_3_)_2_), 1.39–1.26 (m, 19H, C(CH_3_)_2_, OCH_2_CH_2_(CH_2_)_5_CH_3_, Rha-H-6, Fuc-H-6), 0.91–0.89 (m, 12H, SiC(CH_3_)_3_, OCH_2_CH_2_(CH_2_)_5_CH_3_), 0.09 (s, 3H, SiCH_3_), 0.08 (s, 3H, SiCH_3_); ^13^C NMR (125 MHz CDCl_3_): *δ* 138.3 (Ar), 128.3 (Ar), 128.0 (Ar), 127.6 (Ar), 109.0 (C(CH_3_)_2_), 99.3 (Fuc-C-1), 99.1 (Rha-C-1), 80.9 (Rha-C-4), 78.3 (Rha-C-3), 77.9 (Fuc-C-3), 75.9 (Rha-C-2), 73.0 (PhCH_2_), 72.4 (Fuc-C-4), 69.3 (Fuc-C-2), 68.4 (OCH_2_(CH_2_)_6_CH_3_), 65.3 (Rha-C-5), 65.1 (Fuc-C-5), 31.9 (OCH_2_(CH_2_)_6_CH_3_), 29.6 (OCH_2_(CH_2_)_6_CH_3_), 29.4 (OCH_2_(CH_2_)_6_CH_3_), 29.3 (OCH_2_(CH_2_)_6_CH_3_), 27.9 (C(CH_3_)_2_), 26.3 (OCH_2_(CH_2_)_6_CH_3_), 26.1 (C(CH_3_)_2_), 25.7 (SiC(CH_3_)_3_), 22.7 (OCH_2_(CH_2_)_6_CH_3_), 18.0 (SiC(CH_3_)_3_), 17.9 (Rha-C-6), 16.1 (Fuc-C-6), 14.1 (OCH_2_(CH_2_)_6_CH_3_), –4.4 (SiCH_3_), –4.7 (SiCH_3_); HRMS (ESI) Calc. for [M + Na]^+^ C_36_H_62_NaO_9_Si: 689.4055; Found 689.4056.

**Octyl 2,3,4-tri-*O*-acetyl-β-D-xylopyranosyl-(1→4)-[4-*O*-benzyl-2,3-*O*-isopropylidene-α-D-rhamnopyranosyl-(1→3)]-2-*O-tert-*butyldimethylsilyl-α-L-fucopyranoside (S37):** To a stirred solution of acceptor **S36** (136 mg, 204 μmol) and thioglycoside **S16** ^13^ (156 mg, 409 μmol) in dry CH_2_Cl_2_ (5.0 mL) was added molecular sieves (500 mg, 4Å, powder). After stirring for 30 min at room temperature, the reaction mixture was cooled to 0 °C, and then *N*-iodosuccinimide (110 mg, 491 μmol) and silver trifluoromethanesulfonate (10.5 mg, 40.9 μmol) were added successively. The resulting solution was stirred for 1 h at room temperature under an Ar atmosphere. Excess triethylamine was added to quench the acid, the mixture was filtered and the filtrate was washed with saturated Na_2_S_2_O_3_ (aq.) and saturated NaHCO_3_ (aq.), the aqueous layer was extracted with CH_2_Cl_2_ (30 mL × 3), dried over Na_2_SO_4_, filtered and the filtrate was concentrated. The crude residue was purified by flash chromatography (4:1 hexane–EtOAc) to afford **S37** (141 mg, 75%) as a white solid. *R*_f_ 0.24 (4:1 hexane–EtOAc); [α]_D_ –62.1 (*c* 0.47, CHCl_3_); ^1^H NMR (600 MHz; CDCl_3_): *δ* 7.35–7.30 (m, 4H, Ar), 7.27–7.24 (m, 1H, Ar), 5.25 (s, 1H, Rha-H-1), 5.06 (t, 1H, *J* = 6.4 Hz, Xyl-H-3), 4.98 (dd, 1H, *J* = 6.4, 4.7 Hz, Xyl-H-2), 4.93–4.88 (m, 2H, Xyl-H-4, PhCH_2_), 4.65 (d, 1H, *J* = 3.0 Hz, Fuc-H-1), 4.62–4.60 (m, 2H, PhCH_2_, Xyl-H-1), 4.33 (dd, 1H, *J* = 12.3, 4.0 Hz, Xyl-H-5a), 4.21–4.17 (m, 2H, Rha-H-2, Rha-H-3), 4.02–3.98 (m, 2H, Fuc-H-3, Fuc-H-2), 3.96–3.92 (m, 2H, Rha-H-5, Fuc-H-5), 3.63–3.59 (m, 2H, OCH_2_CH_2_(CH_2_)_5_CH_3_, Fuc-H-4), 3.39 (dd, 1H, *J* = 12.3, 5.9 Hz, Xyl-H-5b), 3.34 (dt, 1H, *J* = 9.6, 6.7 Hz, OCH_2_CH_2_(CH_2_)_5_CH_3_), 3.18 (dd, 1H, *J* = 9.8, 7.0 Hz, Rha-H-4), 2.07 (s, 3H, COCH_3_), 2.06 (s, 3H, COCH_3_), 2.02 (s, 3H, COCH_3_), 1.59–1.54 (m, 2H, OCH_2_CH_2_(CH_2_)_5_CH_3_), 1.48 (s, 3H, C(CH_3_)_2_), 1.34–1.23 (m, 16H, C(CH_3_)_2_, OCH_2_CH_2_(CH_2_)_5_CH_3_, Rha-H-6), 1.19 (d, 3H, *J* = 6.6 Hz, Fuc-H-6), 0.89–0.87 (m, 12H, SiC(CH_3_)_3_, OCH_2_CH_2_(CH_2_)_5_CH_3_), 0.08 (s, 3H, SiCH_3_), 0.06 (s, 3H, SiCH_3_); ^13^C NMR (125 MHz CDCl_3_): *δ* 169.8 (C=O), 169.7 (C=O), 169.4 (C=O), 138.7 (Ar), 128.1 (Ar), 127.9 (Ar), 127.4 (Ar), 108.9 (C(CH_3_)_2_), 100.3 (Xyl-C-1), 99.4 (Fuc-C-1), 98.9 (Rha-C-1), 81.3 (Fuc-C-4), 81.2 (Rha-C-4), 78.7 (Rha-C-3), 75.9 (Rha-C-2), 74.8 (Fuc-C-3), 72.7 (PhCH_2_), 70.0 (Fuc-C-2), 69.8 (Xyl-C-3), 69.6 (Xyl-C-2), 68.4 (OCH_2_(CH_2_)_6_CH_3_), 68.1 (Xyl-C-4), 66.0 (Fuc-C-5), 64.6 (Rha-C-5), 60.5 (Xyl-C-5), 31.9 (OCH_2_(CH_2_)_6_CH_3_), 29.6 (OCH_2_(CH_2_)_6_CH_3_), 29.4 (OCH_2_(CH_2_)_6_CH_3_), 29.3 (OCH_2_(CH_2_)_6_CH_3_), 28.0 (C(CH_3_)_2_), 26.3 (OCH_2_(CH_2_)_6_CH_3_), 26.2 (C(CH_3_)_2_), 25.8 (SiC(CH_3_)_3_), 22.7 (OCH_2_(CH_2_)_6_CH_3_), 20.9 (COCH_3_), 20.8 (2 × COCH_3_), 17.9 (SiC(CH_3_)_3_), 17.8 (Rha-C-6), 16.3 (Fuc-C-6), 14.1 (OCH_2_(CH_2_)_6_CH_3_), -4.4 (SiCH_3_), -4.8 (SiCH_3_); HRMS (ESI) Calc. for [M + Na]^+^ C_47_H_76_NaO_16_Si: 947.4795; Found 947.4802.

**Octyl 2,3,4-tri-*O*-acetyl-β-D-xylopyranosyl-(1→4)-[4-*O*-benzyl-2,3-*O*-isopropylidene-α-D-rhamnopyranosyl-(1→3)]-α-L-fucopyranoside (S38):** To a stirred solution of **S37** (141 mg 153 μmol) in THF–pyridine (12 mL, 1:1) was added HF∙pyridine (2.0 mL, pyridine ∼30%, hydrogen fluoride ∼70%) at 0 °C under an Ar atmosphere. The reaction mixture was stirred overnight at room temperature, before being poured into saturated NaHCO_3_ (aq.). The aqueous layer was extracted with EtOAc (30 mL × 3), dried over Na_2_SO_4_, filtered and the filtrate was concentrated. The crude residue was purified by flash chromatography (3:2 hexane–EtOAc) to afford **S38** (110 mg, 89%) as a viscous oil. *R*_f_ 0.20 (2:1 hexane–EtOAc); [α]_D_ –81.8 (*c* 0.11, CHCl_3_); ^1^H NMR (600 MHz; CDCl_3_): *δ* 7.37–7.32 (m, 4H, Ar), 7.29–7.26 (m, 1H, Ar), 5.35 (s, 1H, Rha-H-1), 5.09 (t, 1H, *J* = 6.8 Hz, Xyl-H-3), 5.01 (dd, 1H, *J* = 6.9, 5.1 Hz, Xyl-H-2), 4.93–4.90 (m, 2H, PhCH_2_, Xyl-H-4), 4.82 (d, 1H, *J* = 4.0 Hz, Fuc-H-1), 4.63 (d, 1H, *J* = 11.8 Hz, PhCH_2_), 4.60 (d, 1H, *J* = 5.0 Hz, Xyl-H-1), 4.36–4.32 (m, 2H, Xyl-H-5a, Rha-H-2), 4.24 (t, 1H, *J* = 6.4 Hz, Rha-H-3), 3.98–3.90 (m, 3H, Rha-H-5, Fuc-H-2, Fuc-H-5), 3.86 (dd, 1H, *J* = 10.1, 2.9 Hz, Fuc-H-3), 3.69 (dt, 1H, *J* = 9.8, 6.8 Hz, OCH_2_CH_2_(CH_2_)_5_CH_3_), 3.63 (d, 1H, *J* = 2.8 Hz, Fuc-H-4), 3.45 (dt, 1H, *J* = 9.8, 6.6 Hz, OCH_2_CH_2_(CH_2_)_5_CH_3_), 3.39 (dd, 1H, *J* = 12.3, 6.4 Hz, Xyl-H-5b), 3.22 (dd, 1H, *J* = 9.8, 7.1 Hz, Rha-H-4), 2.08 (s, 3H, COCH_3_), 2.07 (s, 3H, COCH_3_), 2.03 (s, 3H, COCH_3_), 1.94 (d, 1H, *J* = 10.3 Hz, 2-OH), 1.63–1.58 (m, 2H, OCH_2_CH_2_(CH_2_)_5_CH_3_), 1.51 (s, 3H, C(CH_3_)_2_), 1.40 (s, 3H, C(CH_3_)_2_), 1.35–1.26 (m, 13H, OCH_2_CH_2_(CH_2_)_5_CH_3_, Rha-H-6), 1.24 (d, 3H, *J* = 6.6 Hz, Fuc-H-6), 0.90 (t, 3H, *J* = 7.0 Hz, OCH_2_CH_2_(CH_2_)_5_CH_3_); ^13^C NMR (125 MHz CDCl_3_): *δ* 169.9 (C=O), 169.7 (C=O), 169.3 (C=O), 138.6 (Ar), 128.2 (Ar), 127.9 (Ar), 127.4 (Ar), 109.1 (C(CH_3_)_2_), 100.7 (Xyl-C-1), 99.0 (Rha-C-1), 98.5 (Fuc-C-1), 81.1 (Rha-C-4), 80.8 (Fuc-C-4), 78.6 (Rha-C-3), 75.9 (Rha-C-2), 75.8 (Fuc-C-3), 72.7 (PhCH_2_), 69.9 (Xyl-C-3), 69.7 (Xyl-C-2), 69.2 (Fuc-C-2), 68.4 (OCH_2_(CH_2_)_6_CH_3_), 68.2 (Xyl-C-4), 66.5 (Fuc-C-5), 64.8 (Rha-C-5), 60.9 (Xyl-C-5), 31.8 (OCH_2_(CH_2_)_6_CH_3_), 29.5 (OCH_2_(CH_2_)_6_CH_3_), 29.4 (OCH_2_(CH_2_)_6_CH_3_), 29.2 (OCH_2_(CH_2_)_6_CH_3_), 28.0 (C(CH_3_)_2_), 26.4 (C(CH_3_)_2_), 26.2 (OCH_2_(CH_2_)_6_CH_3_), 22.6 (OCH_2_(CH_2_)_6_CH_3_), 20.8 (3 × COCH_3_), 17.9 (Rha-C-6), 16.4 (Fuc-C-6), 14.1 (OCH_2_(CH_2_)_6_CH_3_); HRMS (ESI) Calc. for [M + Na]^+^ C_41_H_62_NaO_16_: 833.3930; Found 833.3934.

**Octyl 2,3,4-tri-*O*-acetyl-β-D-xylopyranosyl-(1→4)-[4-*O*-benzyl-α-D-rhamnopyranosyl-(1→3)]-α-L-fucopyranoside (S39):** To a stirred solution of **S38** (110 mg, 136 μmol) in CH_3_CN–CH_3_OH (6.0 mL, 10:1) was added *p*-toluenesulfonic acid monohydrate (77.5 mg, 408 μmol) at room temperature. The reaction mixture was stirred for 2 h at room temperature. Excess triethylamine was added to quench the acid, the mixture was filtered and the filtrate was concentrated. The crude residue was purified by flash chromatography (15:1 CH_2_Cl_2_–CH_3_OH) to afford **S39** (97.6 mg, 93%) as a syrup. *R*_f_ 0.33 (15:1 CH_2_Cl_2_–CH_3_OH); [α]_D_ –60.7 (*c* 0.26, CHCl_3_); ^1^H NMR (600 MHz; CDCl_3_): *δ* 7.38–7.34 (m, 4H, Ar), 7.32–7.29 (m, 1H, Ar), 5.19 (d, 1H, *J* = 1.6 Hz, Rha-H-1), 5.11 (t, 1H, *J* = 7.7 Hz, Xyl-H-3), 5.03 (dd, 1H, *J* = 7.8, 6.0 Hz, Xyl-H-2), 4.93 (td, 1H, *J* = 7.6, 4.7 Hz, Xyl-H-4), 4.80 (d, 1H, *J* = 11.5 Hz, PhCH_2_), 4.80 (d, 1H, *J* = 4.0 Hz, Fuc-H-1), 4.73 (d, 1H, *J* = 11.5 Hz, PhCH_2_), 4.56 (d, 1H, *J* = 5.9 Hz, Xyl-H-1), 4.29 (dd, 1H, *J* = 12.1, 4.6 Hz, Xyl-H-5a), 4.12–4.11 (m, 1H, Rha-H-2), 4.03–4.01 (m, 1H, Rha-H-3), 3.97–3.87 (m, 3H, Rha-H-5, Fuc-H-5, Fuc-H-2), 3.82 (dd, 1H, *J* = 10.1, 3.0 Hz), 3.70–3.66 (m, 2H, OCH_2_CH_2_(CH_2_)_5_CH_3_, Fuc-H-4), 3.44 (dt, 1H, *J* = 9.8, 6.6 Hz, OCH_2_CH_2_(CH_2_)_5_CH_3_), 3.37 (t, 1H, *J* = 9.1 Hz, Rha-H-4), 3.32 (dd, 1H, *J* = 12.1, 7.7 Hz, Xyl-H-5b), 2.33–2.33 (m, 2H, Rha-2-OH, Rha-3-OH), 2.08 (s, 3H, COCH_3_), 2.05 (s, 3H, COCH_3_), 2.01 (s, 3H, COCH_3_), 1.93 (d, 1H, *J* = 10.5 Hz, Fuc-2-OH), 1.63–1.59 (m, 2H, OCH_2_CH_2_(CH_2_)_5_CH_3_), 1.36-1.27 (m, 13H, Rha-H-6, OCH_2_CH_2_(CH_2_)_5_CH_3_), 1.23 (d, 3H, *J* = 6.6 Hz, Fuc-H-6), 0.90 (t, 3H, *J* = 7.0 Hz, OCH_2_CH_2_(CH_2_)_5_CH_3_); ^13^C NMR (125 MHz CDCl_3_): *δ* 170.1 (C=O), 169.6 (C=O), 169.3 (C=O), 138.6 (Ar), 128.5 (Ar), 127.8 (Ar), 127.7 (Ar), 101.2 (Xyl-C-1), 101.0 (Rha-C-1), 98.5 (Fuc-C-1), 81.8 (Rha-C-4), 80.5 (Fuc-C-4), 75.4 (Fuc-C-3), 74.9 (PhCH_2_), 71.3 (Rha-C-3), 71.1 (Rha-C-2), 70.8 (Xyl-C-3), 70.5 (Xyl-C-2), 69.3 (Fuc-C-2), 68.4 (Xyl-C-4, OCH_2_(CH_2_)_6_CH_3_), 67.6 (Rha-C-5), 66.4 (Fuc-C-5), 61.4 (Xyl-C-5), 31.8 (OCH_2_(CH_2_)_6_CH_3_), 29.5 (OCH_2_(CH_2_)_6_CH_3_), 29.4 (OCH_2_(CH_2_)_6_CH_3_), 29.2 (OCH_2_(CH_2_)_6_CH_3_), 26.2 (OCH_2_(CH_2_)_6_CH_3_), 22.6 (OCH_2_(CH_2_)_6_CH_3_), 20.8 (3 × COCH_3_), 18.2 (Rha-C-6), 16.3 (Fuc-C-6), 14.1 (OCH_2_(CH_2_)_6_CH_3_); HRMS (ESI) Calc. for [M + Na]^+^ C_38_H_58_NaO_16_: 793.3617; Found 793.3618.

**Octyl β-D-xylopyranosyl-(1→4)-[4-*O*-benzyl-α-D-rhamnopyranosyl-(1→3)]-α-L-fucopyranoside (S40):** To a stirred solution of **S39** (97.6 mg, 127 μmol) in CH_3_OH (5.0 mL) was added a solution of NaOCH_3_ in CH_3_OH (0.5 mL, 0.5 M). The reaction mixture was stirred for 2 h at room temperature, then neutralized by addition of Amberlite® IR-120 (H^+^) cation exchange resin, filtered and the filtrate was concentrated. The crude residue was purified by flash chromatography (9:1⟶5:1 CH_2_Cl_2_–CH_3_OH) to afford **S40** (72.0 mg, 88%) as a white amorphous solid. *R*_f_ 0.13 (9:1 CH_2_Cl_2_–CH_3_OH); [α]_D_ –44.1 (*c* 0.17, CH_3_OH); ^1^H NMR (600 MHz; CD_3_OD): *δ* 7.37–7.36 (m, 2H), 7.31–7.29 (m, 2H), 7.24–7.21 (m, 1H), 5.02 (d, 1H, *J* = 1.5 Hz, Rha-H-1), 4.94 (d, 1H, *J* = 11.5 Hz), 4.75 (s, 1H, Fuc-H-1), 4.63 (d, 1H, *J* = 11.5 Hz), 4.24 (d, 1H, *J* = 7.1 Hz, Xyl-H-1), 4.05–3.98 (m, 4H), 3.94 (dd, 1H, *J* = 3.3, 1.7 Hz), 3.87–3.86 (m, 2H), 3.80 (br s, 1H), 3.67–3.63 (m, 1H), 3.49–3.44 (m, 2H), 3.35 (t, 1H, *J* = 9.4 Hz), 3.29–3.24 (m, 2H), 3.06 (dd, 1H, *J* = 11.5, 10.4 Hz), 1.68–1.59 (m, 2H), 1.40–1.27 (m, 13H), 1.20 (d, 3H, *J* = 6.3 Hz), 0.89 (t, 3H, *J* = 7.0 Hz); ^13^C NMR (125 MHz; CD_3_OD): *δ* 140.5, 129.2, 128.8, 128.4, 106.3 (Xyl-C-1), 103.1 (Rha-C-1), 100.7 (Fuc-C-1), 82.9, 81.0, 77.9, 76.1, 75.4 (2 × C), 72.7, 72.6, 71.3, 70.2, 69.6, 69.2, 68.3, 66.7, 33.0, 30.6, 30.5, 30.4, 27.3, 23.7, 18.3, 16.5, 14.4; HRMS (ESI) Calc. for [M + Na]^+^ C_32_H_52_NaO_13_: 667.3300; Found 667.3296.

**Octyl β-D-xylopyranosyl-(1→4)-[α-D-rhamnopyranosyl-(1→3)]-α-L-fucopyranoside (10):** To a stirred solution of **S40** (72.0 mg, 112 μmol) in dry THF (5.0 mL) was added 20% palladium hydroxide on carbon (8.0 mg). After stirring overnight under an H_2_ atmosphere (1 atm), the reaction mixture was filtered through Celite and concentrated. The residue was dissolved in water and then lyophilized to afford **10** (60.3 mg, 97%) as a white solid. *R*_f_ 0.49 (2:1 CH_2_Cl_2_–CH_3_OH); [α]_D_ –55.9 (*c* 0.33, CH_3_OH); ^1^H NMR (600 MHz; CD_3_OD): *δ* 5.03 (d, 1H, *J* = 1.3 Hz, Rha-H-1), 4.75 (s, 1H, Fuc-H-1), 4.26 (d, 1H, *J* = 7.2 Hz, Xyl-H-1), 4.05 (dd, 1H, *J* = 11.6, 5.4 Hz), 4.01 (q, 1H, *J* = 6.7 Hz), 3.96–3.91 (m, 2H), 3.88–3.87 (m, 2H), 3.82 (s, 1H), 3.80 (dd, 1H, *J* = 9.5, 3.4 Hz), 3.66 (dt, 1H, *J* = 9.7, 7.0 Hz), 3.51–3.46 (m, 2H), 3.39 (t, 1H, *J* = 9.4 Hz), 3.29–3.25 (m, 2H), 3.10 (dd, 1H, *J* = 11.4, 10.4 Hz), 1.68–1.59 (m, 2H), 1.41–1.29 (m, 13H), 1.23 (d, 3H, *J* = 6.3 Hz), 0.90 (t, 3H, *J* = 7.0 Hz); ^13^C NMR (125 MHz; CD_3_OD): *δ* 106.2 (Xyl-C-1), 103.3 (Rha-C-1), 100.7 (Fuc-C-1), 81.0, 77.9, 75.5, 75.4, 74.0, 72.2, 72.0, 71.3, 70.2, 70.1, 69.6, 68.4, 66.7, 33.0, 30.6, 30.5, 30.4, 27.3, 23.7, 18.0, 16.6, 14.4; HRMS (ESI) Calc. for [M + Na]^+^ C_25_H_46_NaO_13_: 577.2831; Found 577.2827.

REFERENCE:

1. Speciale I, Laugieri ME, Noel E, Lin S, Lowary TL, Molinaro A, et al. Chlorovirus PBCV‐1 protein A064R has three of the transferase activities necessary to synthesize its capsid protein N‐linked glycans. Proc Natl Acad Sci. 2020;117(46):28735–28742. <https://doi.org/10.1073/pnas.2016626117>
2. Kabsch W. XDS. Acta Crystallogr D Biol Crystallogr. 2010;66(2):125–132. <https://doi.org/10.1107/S0907444909047337>
3. Case DA, Aktulga HM, Belfon K, Cerutti DS, Cisneros GA, Cruzeiro VWD, et al. AmberTools. J Chem Inf Model. 2023;63(20):6183–6191. <https://doi.org/10.1021/acs.jcim.3c01153>
4. Maier JA, Martinez C, Kasavajhala K, Wickstrom L, Hauser KE, Simmerling C. ff14SB: improving the accuracy of protein side chain and backbone parameters from ff99SB. J Chem Theory Comput. 2015;11(8):3696–3713. <https://doi.org/10.1021/acs.jctc.5b00255>
5. Wang J, Wolf RM, Caldwell JW, Kollman PA, Case DA. Development and testing of a General Amber force field. J Comput Chem. 2004;25(9):1157–1174. <https://doi.org/10.1002/jcc.20035>
6. Kirschner KN, Yongye AB, Tschampel SM, González‐Outeiriño J, Daniels CR, Foley BL, et al. GLYCAM06: a generalizable biomolecular force field. Carbohydrates. J Comput Chem. 2008;29(4):622–655. <https://doi.org/10.1002/jcc.20820>
7. Jorgensen WL, Chandrasekhar J, Madura JD, Impey RW, Klein ML. Comparison of simple potential functions for simulating liquid water. J Chem Phys. 1983;79(2):926–935. <https://doi.org/10.1063/1.445869>
8. Andersen HC. Molecular dynamics simulations at constant pressure and/or temperature. J Chem Phys. 1980;72(4):2384–2393. <https://doi.org/10.1063/1.439486>
9. Miyamoto S, Kollman PA. Settle: an analytical version of the SHAKE and RATTLE algorithm for rigid water models. J Comput Chem. 1992;13(8):952–962. <https://doi.org/10.1002/jcc.540130805>
10. Darden T, York D, Pedersen L. Particle mesh Ewald: an *N*·log(*N*) method for Ewald sums in large systems. J Chem Phys. 1993;98(12):10089–10092. <https://doi.org/10.1063/1.464397>
11. Shiozaki M, Tashiro T, Koshino H, Shigeura T, Watarai H, Taniguchi M, et al. Synthesis and biological activity of hydroxylated analogues of KRN7000 (α‐galactosylceramide). Carbohydr Res. 2013;370:46–66. <https://doi.org/10.1016/j.carres.2013.01.010>
12. Takeo K, Nakaji T, Shinmitsu K. Synthesis of lycotetraose. Carbohydr Res. 1984;133(2):275–287. <https://doi.org/10.1016/0008-6215(84)85204-0>
13. Lin S, Lowary TL. Synthesis of a highly branched nonasaccharide chlorella virus *N*‐glycan using a “counterclockwise” assembly approach. Org Lett. 2020;22(19):7645–7649. <https://doi.org/10.1021/acs.orglett.0c02839>
14. Lin S, Lowary TL. Synthesis of the highly branched Hexasaccharide Core of chlorella virus *N*‐linked glycans. Chem A Eur J. 2018;24(64):16992–16996. <https://doi.org/10.1002/chem.201804795>
15. Watt JA, Williams SJ. Rapid, iterative assembly of octyl α‐1,6‐oligomannosides and their 6‐deoxy equivalents. Org Biomol Chem. 2005;3(10):1982–1992. <https://doi.org/10.1039/b503919c>
